# Assessing and improving research readiness in PCORnet®

**DOI:** 10.1017/cts.2025.10207

**Published:** 2025-12-17

**Authors:** Keith Marsolo, Laura Goettinger Qualls, Darcy Louzao, Thomas A. Phillips, Adrian F. Hernandez, Lesley Curtis

**Affiliations:** 1 Duke Clinical Research Institute, Duke University School of Medicine, Durham, NC, USA; 2 Department of Population Health Sciences, Duke University School of Medicine, Durham, NC, USA; 3 Department of Medicine, Duke University School of Medicine, Durham, NC, USA

**Keywords:** Data quality, electronic health records, common data models, distributed research networks, data harmonization

## Abstract

**Purpose:**

We describe the steps taken to assess and improve the research readiness of data within PCORnet®, specifically focusing on the results of the PCORnet data curation process between Cycle 7 (October 2019) and Cycle 16 (October 2024).

**Material and methods:**

We describe the process for extending the PCORnet® CDM and for creating data checks.

**Results:**

We highlight growth in the number of records available across PCORnet between data curation Cycles 7 and 16 (e.g., diagnoses increasing from ∼3.7B to ∼6.9B and laboratory results from ∼7.7B to ∼15.1B among legacy DataMarts), present the current list of data checks and describe performance of the network. We highlight examples of data checks with relatively stable performance (e.g., future dates), those where performance has improved (e.g., RxNorm mapping), and others performance is more variable (e.g., persistence of records).

**Conclusion:**

Studies are a crucial source of information on the design of new data checks. The attention of PCORnet partners is focused primarily on those metrics that are generally modifiable. A transparent data curation process is an essential component of PCORnet, allowing network partners to learn from one another, while also informing the decisions of study investigators on which sites to include in their projects. The quality issues that exist within PCORnet stem from the way that data are captured within healthcare generally. We have been able to make to make great strides on improving data quality and research readiness. Many of the techniques piloted within PCORnet will be broadly applicable to other efforts.

## Background and significance

The 21^st^ Century Cures Act [1], passed by the United States federal government in December 2016, requires that the Food and Drug Administration (FDA) allow the use of real-world evidence (RWE) derived from real-world data (RWD) as part of its regulatory decision-making. Key to this process is ensuring that the underlying data are “fit-for-purpose.” The FDA has published broad guidance on the topic [2], defining fitness in terms of relevance and reliability [3], but there is not yet a consensus on the exact steps needed to demonstrate it. One approach is to follow a two-stage process [4], with the first stage taking raw data and applying a series of transformations and quality checks in order to make them “research ready,” and then applying a second series of transformations and quality checks to ensure that the dataset is “fit-for-purpose” for the specific question at hand. This is the method that the PCORnet® network utilizes as it works to assess and improve data quality across the network.

PCORnet operates as a distributed research network (DRN) [5–7], with funding from the Patient-Centered Outcomes Research Institute (PCORI). Network partners that participate in the network harmonize their data to the PCORnet® Common Data Model (PCORnet® CDM) and respond to queries distributed by the Coordinating Center for PCORnet® (Coordinating Center) via a secure query portal. The types of queries utilized within the infrastructure supported by PCORnet funding includes prep-to-research (PTR) feasibility queries and queries for data curation. For these queries, the underlying datasets stay local, with only query results in the form of aggregate counts or summary statistics being returned.

Within the PCORnet network, data are expected to meet a baseline or foundational level of data quality through a process called data curation [8], which allows for the rapid execution of standardized queries and more efficient initiation of new studies. Once a new project has been proposed, PTR queries and study-specific data characterization (SSDC) activities are encouraged to verify the data are fit-for-purpose [9], examining whether the outcomes and variables of interest are available and complete for the population under study. Data curation is analogous to the first stage described above, while PTR queries and SSDC activities correspond to the second. PCORnet as a network strives to take the model a step further through a process of continuous improvement. Learnings from PTR queries and SSDC activities are fed back into the foundational data curation process [10], which has the effect of raising the baseline level of data quality, allowing the whole network to benefit from the improvements and further decreasing the amount of investigation and mitigation required during future studies.

Data curation is only one part of the process in developing research-ready datasets, however. Effort is required to obtain and standardize the data in the first place. Within a DRN, this occurs through the creation or specification of a CDM which is a harmonized representation of the different data domains available in the source systems of the network partners. Participants within a DRN create individual versions of a CDM that are then used to respond to queries (DataMarts).

## Objective

We describe the steps taken to assess and improve the research readiness of the data within PCORnet over time. We outline the process for modifying the PCORnet® CDM [11], describe extensions to the data curation process, present overall results, and discuss our findings.

## Materials and methods

### Common data model

The initial versions of the PCORnet® CDM were based on the Sentinel CDM [12] to allow the network to repurpose the Sentinel analytical tools. Given that data within the Sentinel Network are primarily sourced from administrative claims and PCORnet relies mostly on data from electronic health records (EHRs), it was recognized that the models would diverge to better reflect the differences in content. Aside from the network’s startup period, when the PCORnet® CDM went through three upgrades in a two-and-a-half-year period, and the COVID-19 pandemic, when major changes were delayed, a new version of the PCORnet® CDM is released every 12–18 months.

Each upgrade follows the same general process. The scope of the update is defined based on the network’s strategic priorities and conversations with PCORnet stakeholders, including PCORI. Additional items may be suggested by other network partners or identified by the Coordinating Center based on findings from studies, prep-to-research queries and data curation. Once the topics have been identified, a landscape scan is performed to determine if there are existing standards that can be used to model them. Common sources to investigate include the Interoperability Standards Advisory [13], the United States Core Data for Interoperability (USCDI) [14], Fast Healthcare Interoperability Resource definitions [15], other data models (e.g., OHDSI, i2b2, Sentinel) [12,16,17], HL7 and CDISC [18]. If a standard is identified, it is important to determine whether partners are capturing data in that format, and if not, whether there are plans to do so in the future. A standard that is not yet widely adopted across EHRs can present a problem, particularly if it is not possible to cleanly map existing data to it (e.g., facility type, payer type, data provenance). In these scenarios, it can sometimes be better to use a custom, PCORnet-defined terminology that is more reflective of the source data until the standard has more uptake.

Once the initial scope of the PCORnet® CDM upgrade has been defined, it is socialized with network partners to gather feedback and assess preferences. Feedback from these activities is used to either confirm the initial approach or make additional modifications. A draft version of the PCORnet® CDM specification is then created and released to network partners for comment. Final edits are made based on these comments. Upgrades typically have a single draft release, though significant ones may have multiple release candidates before the specification is finalized.

The final version of the PCORnet® CDM specification is then presented to network partners for approval. Major releases, which include the addition of new tables, are voted on by the PCORnet® Steering Committee which includes network principal investigators and patient stakeholders. Minor releases, which are limited to modifications of existing tables, are approved by a smaller Executive Management Team, a subset of the Steering Committee. Once the PCORnet® CDM has been approved, partners have approximately 6 months to implement changes before the new version goes into production.

### Data curation

The data curation process was developed and tested in 2015 and fully implemented in January 2016, when the network was on PCORnet® CDM v3.0 (a description of the initial data curation cycle can be found in Qualls et al. [8]). The data curation activities in the PCORnet network occur on a quarterly basis, with updates to the data curation query occurring every 6–9 months (referred to as a data curation cycle). There are 3 main steps to data curation: (1) Partners refresh their DataMart data once every 3 months; (2) They execute the data curation query; (3) They complete an extract-transform-load (ETL) survey which asks about their source system(s), data provenance, steps taken to remediate any data check exceptions, and other details.

The data curation query characterizes all patient-level data from the past 10 years. Each data curation query consists of a set of SAS procedures that executes against the tables of the PCORnet® CDM. These procedures generate hundreds of tables of descriptive statistics, including the frequencies of specific data elements, crosstabs of data (e.g., encounter type per year) and counts of missing, non-missing and distinct records, etc., which are used to evaluate performance against the PCORnet data checks (described below). Additionally, the data from these tables are used to create a report for each DataMart, called the Empirical Data Curation (EDC) report, which includes a summary table listing all the data checks and whether the partner passed or failed each one, as well as dozens of pages of additional charts and tables (See Supplementary Material 1).

Data checks are broad rules applied to the PCORnet® CDM (i.e., required tables are present), with data check measures as specific instantiations of each rule (i.e., the DEMOGRAPHICS table is present). Thus, a single data check may result in dozens of individual measures. Building on the classification from Kahn et al. [19], data checks fall into categories of conformance (“do values adhere to the format of the PCORnet® CDM?”), completeness (“do values appear where we expect them?”), plausibility (“do the values that appear makes sense?,” and persistence (“do large numbers of records appear or disappear between refreshes?”). These are largely the same definitions as those proposed in Khan et al. with some small changes to make the descriptions a little more friendly to lay users (e.g., persistence instead of a variation of temporal plausibility). Most data checks are associated with a threshold (e.g., <5% of records in error). Evidence-based thresholds are used when they exist, otherwise heuristics are derived from network characteristics (e.g., based on network performance at the 80^th^ percentile). Technical and functional specifications are created for each check, and performance is tested against aggregate network data before finalization to ensure there is no unwanted behavior (e.g., all network partners inadvertently fail). The entire data curation package is also beta tested before deployment to the network.

Data checks are classified as required or investigative, and all required data check exceptions must be resolved before a partner’s data are approved for use in any network query. Investigative data checks do not need to be resolved, but network partners are expected to determine whether the issue is remediable or not. If the issue can be resolved over time, they are expected to develop a mitigation plan. New or modified data checks are introduced at the beginning of each cycle to address potential data quality issues and incorporate new tables or fields associated with a PCORnet® CDM upgrade, and are held stable for the remaining refreshes in the cycle.

The composition of the PCORnet network has varied over time, with 8–9 Clinical Research Networks totaling 60–70 DataMarts that represent academic health centers, health systems, hospitals, federally qualified health centers, and claims data; Health Plan Research Networks; patient partners; and a Coordinating Center. We present the evolution of data checks and data check results for all EHR-based DataMarts that participated in Cycles 7 through Cycle 16 (legacy DataMarts), as well as data check results for all EHR-based DataMarts participating in Cycle 16. These cycles cover the network’s data refreshes between October 2019 and October 2024.

## Results

### Common data model

Table 1 shows the release date of each version of the PCORnet® CDM, along with the domains that were added during each release. The initial versions were focused on adding structured data domains most common to EHRs. Version 4.0 saw the introduction of more general-purpose observation tables that provide flexibility when it comes to storing attribute-value type records, though it can still be necessary to create tables for the purposes of analytic efficiency, as was the case with the LDS_ADDRESS_HISTORY table (to support geocoding and surveillance-type research), and HASH_TOKEN (privacy-preserving record linkage). Supplementary Material 2 provides a description of the content of each table of the PCORnet® CDM.

**Table 1.** Release date of each version of the PCORnet® CDM and the tables (data domains) that were added during each release. Minor releases (e.g., X.1) involve changes to existing tables and do not include the addition of new domains

Version 1.0 (May 30, 2014)	Version 2.0 (Feb. 27, 2015)	Version 3.0 (June 1, 2015) Version 3.1 (Nov. 15, 2016)	Version 4.0 (Jan. 2, 2018) Version 4.1 (May 15, 2018)	Version 5.0 (July 16, 2019) Version 5.1 (Sept. 10, 2019)	Version 6.0 (Oct. 22, 2020) Version 6.1 (April 3, 2023)	Version 7.0 (Dec. 23, 2024)
DEMOGRAPHIC	CONDITION	DEATH	MED_ADMIN	HASH_TOKEN	LAB_HISTORY	EXTERNAL_MEDS
DIAGNOSIS	DISPENSING	DEATH_CAUSE	PROVIDER	IMMUNIZATION		PAT_RELATIONSHIP
ENROLLMENT	LAB_RESULT_CM	PRESCRIBING	OBS_CLIN	LDS_ADDRESS_ HISTORY		PRO_CM (restructured)
ENCOUNTER	PRO_CM	HARVEST	OBS_GEN			
PROCEDURES		PCORNET_TRIAL	PRO_CM (restructured)			
VITAL						

Table 2 provides a network-level summary of the different data domains available at the end of data curation cycle 16, along with a comparison between cycles 7 and 16 for legacy DataMarts. Shown are the number of records in key categories, as well as the number of unique concepts (codes) that are used to represent them. Between Cycle 7 and Cycle 16, among legacy DataMarts, the number of patients with at least 1 face-to-face encounter in the past year increased from approximately 25.4 million to approximately 36.9 million. Within the legacy DataMarts, the number of diagnosis records increased from approximately 3.7 billion to approximately 6.9 billion, even as the number of ICD-9 coded records decreased due to the transition to ICD-10 (a similar trend was seen with procedure records).

**Table 2.** Growth of records across the network over time. Results are shown for the PCORnet legacy DataMarts (EHR-based DataMarts that participated during cycles 7 through 16) and for all EHR-based DataMarts that participated in cycle 16 refresh 2

	Legacy DataMarts			All DataMarts
	Cycle 7 refresh 1 (October 2019)	Cycle 16 refresh 2 (October 2024)	Percent change	Cycle 16 refresh 2 (October 2024)
DataMarts	51	51		74
Dates for record-level data	2010–2019	2015–2024		2015–2024
**Demographics**				
# of patients	80,648,736	132,355,814	64.1	182,584,892
# of patients with >=1 face-to-face encounter in the past 5 years	54,405,202	72,394,407	33.1	98,221,542
# of patients with >=1 face-to-face encounter in the past year	25,398,937	36,859,856	45.1	48,904,318
**Encounter**				
# of records	1,821,121,020	3,203,886,300	75.9	4,678,981,685
# Ambulatory visit records	1,026,960,840	1,401,137,191	36.4	1,914,939,225
# Emergency department or inpatient stay (ED / EI / IP) records	100,046,278	133,751,412	33.7	188,308,783
# Telehealth (TH) records	0	59,129,068		141,644,497
# Observation stay (OS) records	2,343,532	7,796,516	232.7	11,254,421
# Other ambulatory (OA) records	44,7771,647	1,075,205,689	140.1	1,541,112,317
**Diagnoses**				
# of records	3,740,458,279	6,923,486,138	85.1	9,594,629,641
# ICD-9 records (codes)	1,663,010,817 (30,034)	384,051,618 (28,794)	−76.9	524,137,778 (29,077)
# ICD-10 records (codes)	1,983,219,172 (73,022)	6,494,316,729 (82,184)	227.5	9,023,671,015 (83,850)
**Procedures**				
# of records	3,785,760,402	7,347,560,235	94.1	9,541,694,638
# CPT records (codes)	3,545,366,291 (23,363)	6,930,099,639 (30,619)	95.5	8,722,297,920 (34,962)
# ICD9 records (codes)	63,336,548 (30,034)	14,717,350 (28,794)	−76.8	18,369,000 (29, 077)
# ICD10 records (codes)	27,197,534 (42,084)	97,060,832 (51,236)	256.9	143,951,692 (53,611)
**Prescribing**				
# of records	2,152,928,302	3,998,057,492	85.7	5,137,515,208
# RxNorm records (codes)	2,054,391,804	3,775,232,044	83.8	4,853,453,805
**Labs**				
# of records	7,707,547,928	15,150,011,115	96.6	20,102,526,059
# LOINC records (codes)	7,386,451,565	14,556,940,093	97.1	19,387,415,534
# LOINC codes per dataMart, median (min, max)	1,364 (6, 6459)	2,585 (155,9454)	89.5	2,878 (155, 9454)
**Medication administration**				
# of DataMarts	36	46		69
# of records	1,685,553,966	4,333,912,221	157.1	5,738,711,473
# RxNorm records (codes)	1,413,757,427 (28,964)	3,693,356,616 (32,069)	161.2	4,892,440,490 (37,170)
# NDC records (codes)	161,581,296 (31,631)	480,641,726 (59,735)	197.5	664,181,638 (65,964)

Table 2 also shows the increase in laboratory results across the network over time. Historically, laboratory results were coded using custom terminologies. Networks like PCORnet have focused a great deal of attention on harmonizing these data to standard terminologies, like LOINC, so the results can be used in multi-site analyses. Table 2 shows the median number of unique LOINC codes per DataMart, as well as the total number of laboratory results that are associated with a LOINC code. From Cycle 7 to Cycle 16, the median number of LOINCs has grown to more than 2500, and the total number of records has grown from ∼7.4 billion to ∼14.5 billion.

### Data curation

**Figure 1.**
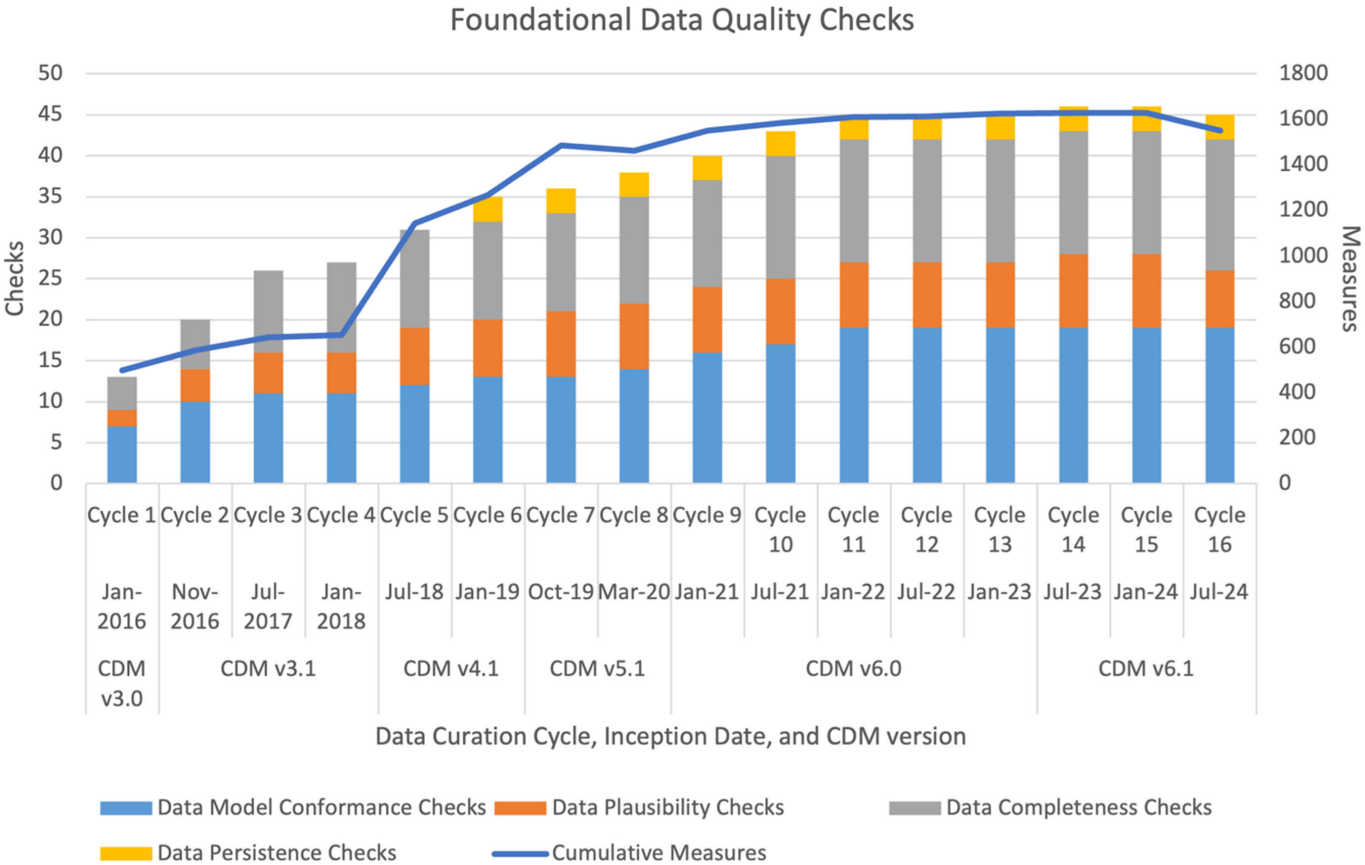
Growth in foundational data quality checks over time. Each bar represents the start of a new data curation cycle. Listed are the dates the cycle started, corresponding version of the PCORnet® CDM and number of data checks and measures. Data checks are broad rules such as “Values must conform to CDM specifications.” Measures are the number of PCORnet® CDM tables and/or fields affected by the checks.

The evolution of the data checks used in PCORnet is illustrated in Figure 1. Each bar represents the overall number of data checks, with the type of check stratified by color (e.g., conformance, plausibility, completeness, persistence). The line graph shows the number of data check measures that correspond to data checks for each cycle. The start date of each cycle is listed, along with the version of the PCORnet® CDM that was in use at that time. Of note, the cumulative data checks (measures) increased from 36 (1487) in Cycle 7 to 46 (1551) in Cycle 16.

A high-level overview of the data checks and overall network performance for selected curation cycles is shown in Table 3. The table lists the dates of each curation cycle, the number of required and investigative data checks, and performance, in terms of the number of investigative data check exceptions (median, minimum and maximum) and the percentage of DataMarts with fewer exceptions between cycles. The median number of investigative data check exceptions has decreased over time, from around 6 to 4, while the minimum and maximum numbers have been relatively stable (minimum ranging between 0–1, maximum between 11–16). The data checks used in data curation cycle 16 are listed in Table 4, along with any changes that occurred since cycle 7 (e.g., changes in number of data check measures). Table 4 contains a description of each data check. Overall network performance on the investigative data checks in Cycles 7 and 16 is also shown in Table 4. For each data check, we shown the number and percentage of DataMarts with an exception. Performance ranges from 0% (no exceptions) to 63% (32 of 51 legacy DataMarts with an exception).

**Table 3.** Overview of data checks by cycle and network performance (overall and compared to prior refreshes). To improve readability, we report the results of alternating cycles between cycles 7 and 15

	Cycle 7	Cycle 9	Cycle 11	Cycle 13	Cycle 15	Cycle 16
Common data model (CDM) version	V5.1	V6.0	V6.0	V6.0	V6.1	V6.1
DataMart refresh dates	Oct 2019 Jan 2020	Jan 2021 Apr 2021	Jan 2022 Apr 2022	Jan 2023 Apr 2023	Jan 2023 Apr 2023	Jul 2024 Oct 2024
Required data checks (measures)	15 (1165)	16 (1243)	17 (1264)	18 (1268)	18 (1268)	18 (1268)
Investigative data checks (measures)	21 (322)	24 (309)	28 (346)	27 (359)	28 (362)	27 (283)
Investigative data check exceptions, median (min, max)	6 (0,14)	5.5 (1,12)	5 (1, 13)	4 (0,14)	5 (0,16)	4 (0, 13)
DataMarts with fewer investigative data check exceptions than in the previous cycle, *n* (%)	n/a	27/50 (54%)	20/55 (36%)	30/59 (51%)	25/71 (35%)	37/74 (50%)

**Table 4.** Evolution of PCORnet data checks over time. Shown are the data checks that were active as of data curation cycle 16, the type of check (investigative or required), and % of legacy DataMarts (*n* = 51) with exceptions. One data partner was not approved for the second refresh of cycle 7, and denominators for some data checks are lower if some partners do not have the applicable data

Data check	Data check description	Measures in cycle 7 and cycle 16	Classification	Legacy DataMarts with an exception, *N* (%)	
				Cycle 7 refresh 2	Cycle 16 refresh 2
**Data model conformance**					
DC 1.01	Required tables are not present. (This check is applied to all tables.)	22, 23	Required	—	—
DC 1.02	Required tables are not populated. (This check is applied to the DEMOGRAPHIC, ENROLLMENT, ENCOUNTER, DIAGNOSIS, PROCEDURES, and HARVEST tables.)	6, 6	Required	—	—
DC 1.03	Required fields are not present. (This check is applied to all fields.)	416, 442	Required	—	—
DC 1.04	Required fields do not conform to the data model specifications for data type, length, or name. (This check is applied to all fields.)	416, 442	Required	—	—
DC 1.05	Tables have primary key definition errors. (This check is applied to all tables.)	22, 23	Required	—	—
DC 1.06	Required fields contain values outside of data model specifications. (This check is applied to all fields with value sets.)	131, 142	Required	—	—
DC 1.07	Required fields have non-permissible missing values. (This check is applied to all fields that are required to be populated.)	71, 73	Required	—	—
DC 1.08	Tables contain orphan PATIDs (PATIDS that are not present in the DEMOGRAPHIC table). This check is applied to the CONDITION, DIAGNOSIS, DEATH, DEATH_CAUSE, DISPENSING, ENCOUNTER, ENROLLMENT, HASH_TOKEN, IMMUNIZATION, LAB_RESULT_CM, LDS_ADDRESS_HIST, MED_ADMIN, OBS_CLIN, OBS_GEN, PCORNET_TRIAL, PRESCRIBING, PROCEDURES, PRO_CM, and VITAL tables.	19, 19	Required	—	—
DC 1.09	Tables contain orphan ENCOUNTERIDs (ENCOUNTERIDs that are not present in the ENCOUNTER table) for more than 5% of records. This check is applied to the CONDITION, DIAGNOSIS, IMMUNIZATION, LAB_RESULT_CM, MED_ADMIN, OBS_CLIN, OBS_GEN, PRESCRIBING, PROCEDURES, PRO_CM, and VITAL tables.	11, 11	Required	—	—
DC 1.10	Replication errors between the ENCOUNTER, PROCEDURES and DIAGNOSIS tables. (Replication errors are ENCOUNTERIDs in the DIAGNOSIS or PROCEDURES table where the encounter type or admit date does not match the corresponding value in the ENCOUNTER table.)	4, 4	Required	—	—
DC 1.11	More than 5% of encounters are assigned to more than one patient. (This check is applied to all tables that include ENCOUNTERID [CONDITION, DIAGNOSIS, ENCOUNTER, IMMUNIZATION, LAB_RESULT_CM, MED_ADMIN, OBS_CLIN, OBS_GEN, PRESCRIBING, PROCEDURES, PRO_CM, and VITAL].)	12, 12	Required	—	—
DC 1.12	Tables contain orphan PROVIDERIDs (PROVIDERIDs that are not present in the PROVIDER table). This check is applied to the ENCOUNTER, DIAGNOSIS, IMMUNIZATION, MED_ADMIN, OBS_CLIN, OBS_GEN, PRESCRIBING, and PROCEDURES tables.	8, 8	Required	—	—
DC 1.13	More than 5% of CPT/HCPCS, CVX, ICD, LOINC, NDC, or RXNORM codes do not conform to the expected length or content using terminology-specific heuristics. (This check is applied to the records in CONDITION, DIAGNOSIS, DISPENSING, IMMUNIZATION, LAB_HISTORY, LAB_RESULT_CM, MED_ADMIN, OBS_CLIN, OBS_GEN, PRESCRIBING and PROCEDURES tables.)	25, 27	Required	—	—
DC 1.14	Patients in the DEMOGRAPHIC table are not in the HASH_TOKEN table	0, 1	Investigative	—*	9/51 (17.6%)
DC 1.15	Fields with undefined lengths that are present in more than one table (PATID, ENCOUNTERID, PRESCRIBINGID, PROCEDURESID, PROVIDERID, MEDADMIN_PROVIDERID, OBSGEN_PROVIDERID, OBSCLIN_PROVIDERID, RX_PROVIDERID, and VX_PROVIDERID) do not have harmonized field lengths. This check is applied to the DEMOGRAPHIC, ENCOUNTER, ENROLLMENT, DIAGNOSIS, PROCEDURES, VITAL, DISPENSING, LAB_RESULT_CM, CONDITION, PRO_CM, PRESCRIBING, PCORNET_TRIAL, PROVIDER, DEATH, DEATH_CAUSE, MED_ADMIN, OBS_CLIN, OBS_GEN, HASH_TOKEN, LDS_ADDRESS_HISTORY and IMMUNIZATION tables.	0, 10	Required	—*	—
DC 1.16	Laboratory results or clinical observations are recorded in the wrong table. (This check is applied to the LAB_RESULT_CM table [LOINC codes that are not classtype 1 (laboratory class)] and the OBS_CLIN table [LOINC codes that are not classtype 2 (clinical class)].)	0, 4	Investigative	—*	3/51 (5.9%)
DC 1.17	Zip codes in the ENCOUNTER or LDS_ADDRESS_HISTORY table do not conform to expected values. (Non-conforming values are defined as values that contain alphabetical characters or do not have the expected number of digits.)	0, 3	Required	—*	—
DC 1.18	Table refresh dates are not documented for tables that are populated. (This check is applied to the HARVEST table.)	0, 21	Required	—*	—
DC 1.19	More than 10% of hash tokens in the HASH_TOKEN table are assigned to multiple patients (within a given DataMart)	0, 6	Investigative	—*	4/50 (8.0%)
**Data plausibility**					
DC 2.01	More than 5% of records have future dates. (Future dates are calculated as those with dates occurring after the maximum refresh date in the HARVEST table. This check is applied to the date fields in all tables except PRIVATE_ADDRESS_HISTORY, PRIVATE_ADDRESS_GEOCODE, and PRIVATE_DEMOGRAPHIC.)	30, 31	Investigative	2/50 (4.0%)	2/51 (3.9%)
DC 2.02	More than 10% of records fall into the lowest or highest categories of age (<0 or >89), height (<0 inches or >95 inches), weight (<0 lbs or >350 lbs), diastolic blood pressure (<40 mmHg or >120 mmHg), systolic blood pressure (<40 mmHg or >210 mmHg), or dispensed days supply (<1 day or >90 days). (This check is applied to the DEMOGRAPHIC, DISPENSING, and VITAL tables.)	12, 12	Investigative	5/50 (10.0%)	4/51 (7.8%)
DC 2.03	More than 5% of patients have illogical date relationships. (Illogical date relationships are defined as dates of service in the DEATH, DISPENSING, ENCOUNTER, LAB_RESULT_CM, PRESCRIBING, PROCEDURES, VITAL, MED_ADMIN, OBS_CLIN, or OBS_GEN table which occur before a patient’s birth date or after a patient’s death date; procedure dates occurring more than 5 days before the admit date or more than five days after the discharge data for the same encounter in the PROCEDURES table; or a start date before the end date in the OBS_CLIN and OBS_GEN tables.)	26, 32	Investigative	10/50 (20.0%)	8/51 (15.7%)
DC 2.04	The average number of encounters per visit is >2.0 for inpatient (IP), emergency department (ED), or ED to inpatient (EI) encounters. (A visit is defined as a unique combination of PATID, ENC_TYPE, ADMIT_DATE, and PROVIDERID in the ENCOUNTER table.)	3, 3	Investigative	0/50 (0%)	0/51 (0%)
DC 2.07	The average number of principal diagnoses per known DX_ORIGIN per encounter is above threshold [2.0 for inpatient (IP) and ED to inpatient (EI)]. (Results are calculated using the PDX, DX_ORIGIN, ENCOUNTERID, and ENC_TYPE fields in the DIAGNOSIS table.)	2, 8	Investigative	8/45 (17.8%)	9/47 (19.1%)
DC 2.08	The monthly volume of encounter, diagnosis, procedure, vital, prescribing, medication administration or laboratory records is an outlier. (Encounter types of interest are AV (Ambulatory Visit), ED (Emergency Department), EI (Emergency Department Admit to Inpatient Hospital Stay), and IP (Inpatient Hospital Stay). Outliers are defined as months with 0 records or a significant decrease compared to the average volume in the previous 12 months.)	6, 6	Investigative	21/50 (42.0%)	16/51 (31.4%)
DC 2.09	Less than 80% of patients with a face-to-face encounter during the past 5 years have at least 1 face-to-face diagnosis and 1 vital measurement. (Face-to-face is defined as an encounter type of ambulatory visit (AV), emergency department (ED), emergency department admit to inpatient hospital stay (EI), inpatient hospital (IP), or observation stay (OS).)	0, 1	Investigative	—*	10/51 (19.6%)
**Data completeness**					
DC 3.01	The average number of diagnoses records with known diagnosis types per encounter is below threshold [1.0 for ambulatory (AV), inpatient (IP), emergency department (ED), ED to inpatient (EI), or telehealth (TH) encounters]. (Results are calculated using the DIAGNOSIS and ENCOUNTER tables.)	4, 5	Investigative	2/50 (4.0%)	0/51 (0%)
DC 3.02	The average number of procedure records with known procedure types per encounter is below threshold [0.75 for ambulatory (AV) encounters, 0.75 for emergency department (ED) encounters, 1.00 for ED to inpatient (EI) encounters, and 1.00 for inpatient (IP) encounters]. (Results are calculated using the PROCEDURES and ENCOUNTER tables.)	4, 4	Investigative	6/50 (12.0%)	3/51 (5.9%)
DC 3.03	More than 10% of records have missing or unknown values for the following fields: DISCHARGE_DISPOSITION (IP/EI encounters only), DISPENSE_SUP, DX_SOURCE, SEX, code fields [DEATH_CAUSE_CODE, MEDADMIN_CODE, OBSCLIN_CODE, OBSGEN_CODE], code type fields [DX_TYPE, CONDITION_TYPE, MEDADMIN_TYPE, OBSCLIN_TYPE, OBSGEN_TYPE, PX_TYPE, VX_CODE_TYPE], date fields [BIRTH_DATE, DISCHARGE_DATE (IP/EI encounters only), RX_ORDER_DATE, PX_DATE, VX_RECORD_DATE], ENCOUNTERID fields in selected tables [DIAGNOSIS, LAB_RESULT_CM, PRESCRIBING, MED_ADMIN, OBS_CLIN, PROCEDURES, and VITAL], and provenance fields [CONDITION_SOURCE, DEATH_CAUSE_SOURCE, DEATH_SOURCE, DISPENSE_SOURCE, DX_ORIGIN, MEDADMIN_SOURCE, LAB_RESULT_SOURCE, PX_SOURCE, RX_SOURCE, VITAL_SOURCE, VX_SOURCE]	30, 38	Investigative	35/50 (70.0%)	32/51 (62.7%)
DC 3.04	Less than 50% of patients with encounters have DIAGNOSIS records	1, 1	Required	—	—
DC 3.05	Less than 50% of patients with encounters have PROCEDURES records	1, 1	Required	—	—
DC 3.06	More than 10% of IP (inpatient) or ED to inpatient (EI) encounters with any diagnosis from a known DX_ORIGIN do not have a principal diagnosis from that source. (Results are calculated using the PDX, DX_ORIGIN, ENCOUNTERID, and ENC_TYPE fields in the DIAGNOSIS table.)	2, 8	Investigative	10/45 (22.2%)	11/47 (23.4%)
DC 3.07	Encounters, diagnoses or procedures in an ambulatory (AV), telehealth (TH), emergency department (ED), ED to inpatient (EI), or inpatient (IP) setting are less than 75% complete two months prior to the current month. (Data completeness is calculated by comparing the actual volume to the average volume during the 1 year prior to the current date.)	3, 3	Investigative	13/50 (26.0%)	5/51 (9.8%)
DC 3.08	Less than 80% of prescribing orders are mapped to a RXNORM_CUI which fully specifies the ingredient, strength and dose form (i.e. RXCUI codes that have a Term Type of SCD, SBD,BPCK, or GPCK).	2, 2	Investigative	8/50 (16.0%)	4/51 (7.8%)
DC 3.09	Less than 80% of laboratory results are mapped to LAB_LOINC and have either a quantitative result (RESULT_NUM is not null and RESULT_MODIFIER is not NI, UN, OT, or null) or a qualitative result (RESULT_QUAL is not NI, UN, OT, or null)	2, 2	Investigative	3/50 (6.0%)	3/51 (5.9%)
DC 3.10	Less than 80% of quantitative results for tests mapped to LAB_LOINC fully specify the normal range. (A fully specified normal range is defined as follows: [NORM_MODIFIER_LOW=’EQ’ and NORM_MODIFIER_HIGH=’EQ’ and NORM_RANGE_LOW is not null and NORM_RANGE_HIGH is not null] *or* [NORM_MODIFIER_LOW in (“GT,” “GE”) and NORM_MODIFER_HIGH=“NO” and NORM_RANGE_LOW is not null and NORM_RANGE_HIGH is null] *or* [NORM_MODIFIER_HIGH in (“LE,” “LT”) and NORM_MODIFIER_LOW=’NO’ and NORM_RANGE_HIGH is not null and NORM_RANGE_LOW is null].)	2, 2	Investigative	21/50 (42.0%)	10/51 (19.6%)
DC 3.11	Vital, prescribing, or laboratory records are less than 75% complete three months prior to the current month. (Data completeness is calculated by comparing the actual volume to the average volume during the 1 year prior to the current date.)	3, 3	Investigative	16/50 (32.0%)	3/51 (5.9%)
DC 3.12	Less than 80% of quantitative results for tests mapped to LAB_LOINC fully specify the result unit (i.e. RESULT_NUM is not null and RESULT_UNIT is not NI, UN, OT, or null).	2, 2	Investigative	27/50 (54.0%)	3/51 (5.9%)
DC 3.14	Medication administration, dispensing, or clinical observation records are less than 75% complete three months prior to the current month. (Data completeness is calculated by comparing the actual volume to the average volume during the 1 year prior to the current date.)	0, 3	Investigative	—*	20/51 (39.2%)
DC 3.15	Less than 80% of medication administrations mapped to RXNORM are mapped to a RXNORM_CUI that fully specifies the ingredient, strength and dose form (i.e. RXCUI codes that have a Term Type of SCD,SBD,BPCK, or GPCK).	0, 2	Investigative	—*	5/39 (12.8%)
DC 3.16	Less than 80% of clinical observations are mapped to an OBSCLIN_CODE and have a quantitative result (OBSCLIN_RESULT_NUM is not null and OBSCLIN_RESULT_MODIFIER is not NI,UN,OT or null), qualitative result (OBSCLIN_RESULT_QUAL is not NI,UN,OT or null) or narrative result (OBSCLIN_RESULT_TEXT is not null).	0,1	Investigative	—*	8/46 (17.4%)
DC 3.17	Less than 80% of quantitative results for tests mapped to OBSCLIN_CODE fully specify the RESULT_UNIT (i.e. OBSCLIN_RESULT_NUM is not null and OBSCLIN_RESULT_UNIT is not NI, UN, OT, or null).	0,1	Investigative	—*	8/35 (22.9%)
**Data persistence**					
DC 4.01	More than a 5% decrease in the number of patients or records in a CDM table between the current and previous DataMart refresh. (This check is applied to the DEMOGRAPHIC, ENROLLMENT, ENCOUNTER, DIAGNOSIS, PROCEDURES, VITAL, DEATH, PRESCRIBING, DISPENSING, LAB_RESULT_CM, CONDITION, DEATH_CAUSE, PRO_CM, PROVIDER, MED_ADMIN, OBS_CLIN, OBS_GEN, HASH_TOKEN, IMMUNIZATION, LDS_ADDRESS_HISTORY and LAB_HISTORY tables.)	39, 39	Investigative	21/50 (42.0%)	15/51 (29.4%)
DC 4.02	More than a 5% decrease in the number of patients or records for diagnosis, procedures, labs or prescriptions during an ambulatory (AV), telehealth (TH), other ambulatory (OA), emergency department (ED), or inpatient (IP) encounter between the current and previous DataMart refresh.	32, 40	Investigative	12/50 (24.0%)	9/51 (17.6%)
DC 4.03	More than a 5% decrease in the number of records or distinct codes for CPT/HCPCS, CVX, ICD10, NDC, or RXNORM codes between the current and previous DataMart refresh. (This check is applied to the DIAGNOSIS, DISPENSING, IMMUNIZATION, MED_ADMIN, PRESCRIBING and PROCEDURES tables.)	10, 24	Investigative	9/50 (18.0%)	11/51 (21.6%)

*Indicates data check was added after cycle 7. DX = diagnosis; PX = procedures.

We provide additional detail on data check performance in the figures below. Results tend to fall into one of four categories: (1) network performance is consistently high (i.e., all, or almost all DataMarts pass the check as soon as it is introduced or shortly thereafter); (2) network performance improves over time; (3) network performance is stable and the same DataMarts have exceptions; and (4) network performance is stable but different DataMarts have exceptions over time.

All of the required data checks fall into category #1, as DataMarts must resolve any issues before their refresh can be approved. Investigative checks in this category include DC2.04 (encounters per visit), DC3.01 (diagnoses per encounter), and many of the variables that are checked as part of DC3.03 (missingness), including birth date and many provenance fields.

**Figure 2.**
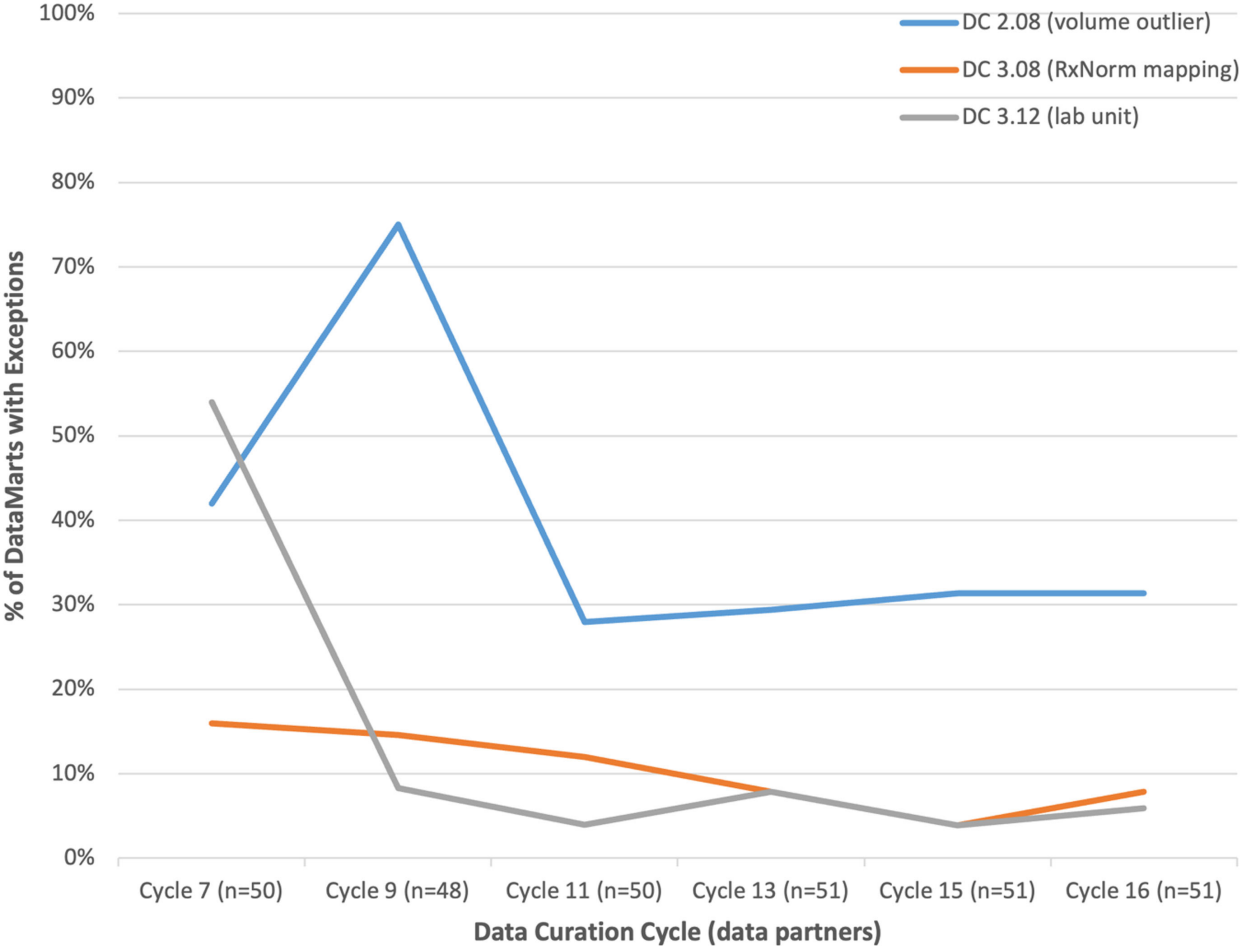
Improvement in data check performance over time. The spike in DC2.08 is due to volumes being affected by the COVID-19 pandemic. The expectation is that all EHR-based DataMarts should be able to eventually pass these checks.

Data checks in category #2, where performance has improved over time, include DC2.08 (volume outliers), DC3.08 (RxNorm mapping), and DC3.12 (lab units). These checks are shown in Figure 2. For DC3.12, 54% of DataMarts had exceptions in Cycle 7. In Cycle 16, it was only ∼6%. For DC3.08, the percentage of DataMarts with exceptions decreased from 16% to ∼8%. For DC2.08, 42% of DataMarts had exceptions in Cycle 7 and in Cycle 16 it was ∼30% (the spike in Cycle 9 was due to the changes in healthcare volumes during the shutdowns related to the COVID-19 pandemic). One of the characteristics about the data checks in this category is that they tend to deal with relatively modifiable factors – mapping source data to a reference terminology like LOINC or RxNORM or making changes to an ETL process to shorten the refresh timing.

**Figure 3.**
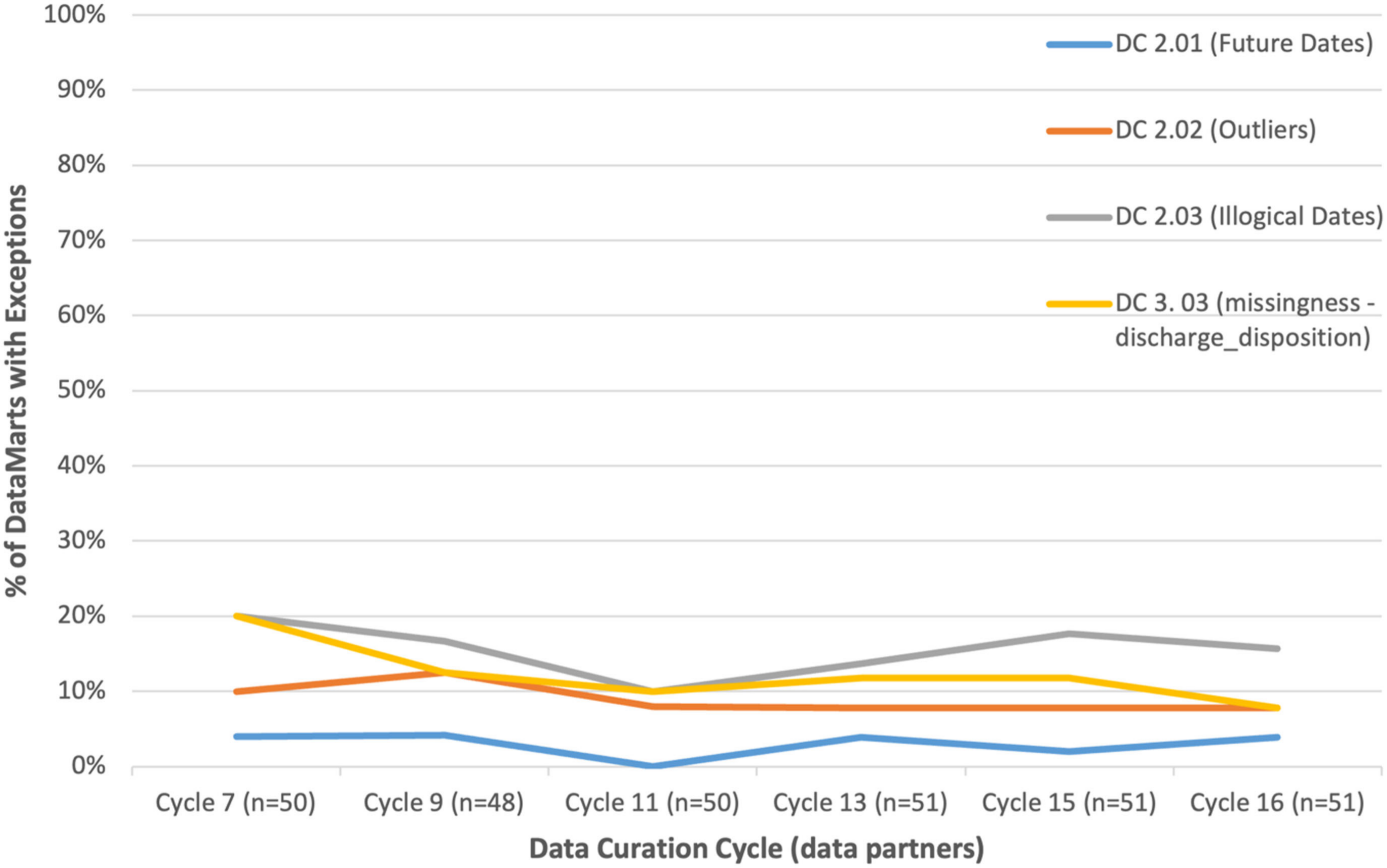
Data checks with relatively stable performance, where the DataMarts with exceptions tend to also be stable. Each line presents the percentage of DataMarts with an exception during a given data curation cycle.

Examples of checks that fit into category #3 – stable performance with the same DataMarts having issues – are shown in Figure 3. They include many of the data plausibility checks, including DC2.01 (future dates), DC2.02 (outliers), DC2.03 (illogical dates), and many aspects of DC3.03 (missingness), such as discharge disposition and days supply for prescribing records (results for the check related to discharge disposition are shown in the figure). Exceptions to these checks tend to occur due to workflow or source data limitations (e.g., days supply is not populated as part of a medication order, discharge disposition is not populated if the patient is alive at discharge) and are generally not remediable.

**Figure 4.**
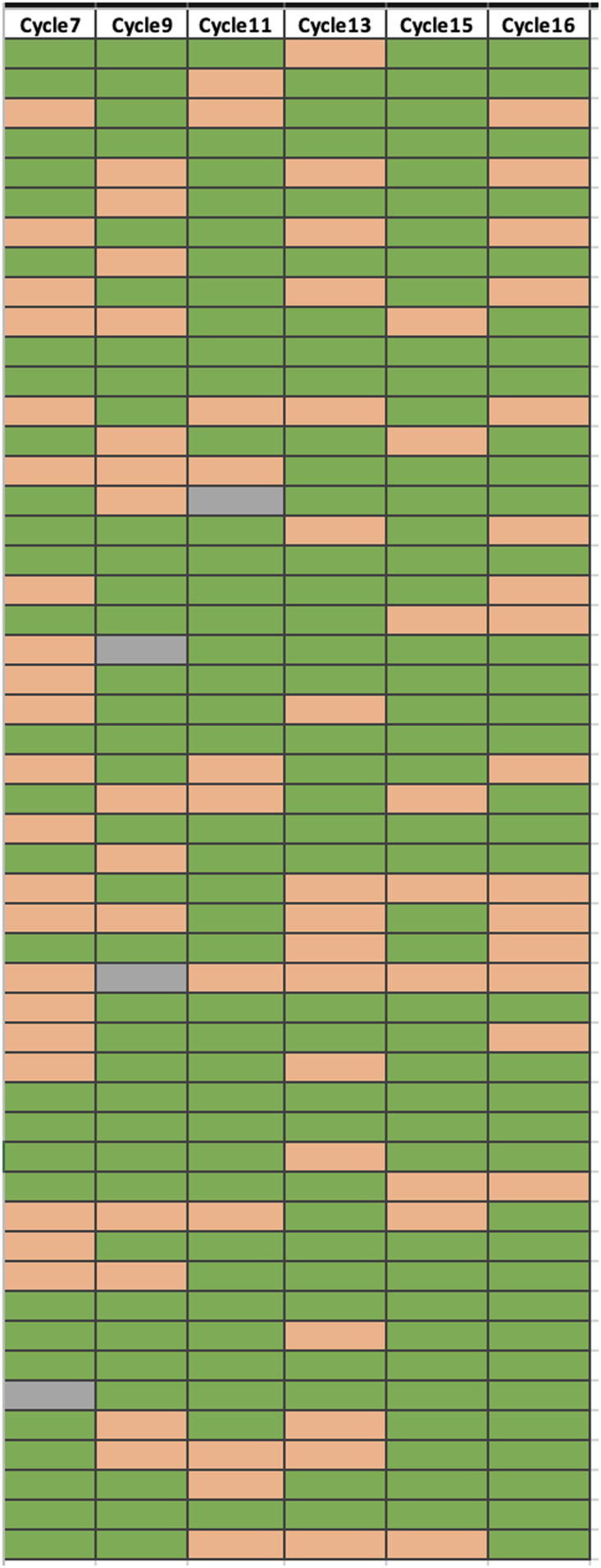
Example of DC4.01, a data check where network performance is stable, but the DataMarts with exceptions tends to change over time. Each row represents a DataMart, and each column a refresh. If a DataMart had an exception for the check, the cell is shaded orange. No exception is shaded green. A gray cell indicates that a DataMart did not submit new data for that refresh or was not approved.

The checks related to persistence (DC4.01–DC4.03) are examples of checks in category #4, where performance is relatively stable, but the mix of DataMarts with exceptions changes from refresh to refresh. For the persistence checks, exceptions may occur because of a loss of a data feed (temporary or permanent), onboarding a new hospital or physician practice, changes in ETL processes, or updated mapping strategies (e.g., how to assign ICD-10 codes). An example is shown in Figure 4, which shows results for DC4.01 for cycles 7 through 16. Each row in the figure represents a DataMart, and each column a different refresh. If a DataMart had an exception for the check, the cell is shaded orange. No exception is shaded green. A gray cell indicates that a DataMart did not submit new data for that refresh or was not approved. This demonstrates the need to monitor changes over time, since studies that utilize these data need to be sure that there are not large changes in the underlying source data/population between refreshes or study queries (or compared to baseline if they are relying on a single extract).

## Discussion

A transparent data curation process is an essential component of a DRN, allowing network partners to learn from one another, while also informing the decisions of study investigators on which sites to include in their project. In designing the PCORnet data curation process, the Coordinating Center tried to strike a balance between a completely centralized process like the one found within Sentinel and a voluntary or “self-attesting” process like that of the OHDSI community. Within prior iterations of the Sentinel network, participants (data partners) submitted results to the Operations Center and then received feedback on whether issues need remediation after the results have been reviewed. Given the number of network partners within PCORnet, the Coordinating Center wanted to implement a more streamlined process, with a curation package that partners could execute with results that made it clear whether they passed or failed. In this manner, having an organized, coordinated process can help ensure a level of accountability that may be missing if curation was more of a voluntary or “self-attesting” exercise. It is important to note that PCORnet does not ask network partners to delete records or impute values if there are quality issues. While individual study teams may make those decisions as part of their analysis, we believe it is important to present the quality of the data as they are, as different study designs may have varying thresholds for what is considered a minimum level of data quality. For instance, a study team may not want to include a site that has imputed 50% of its laboratory result units for a retrospective data analysis, but the site may be perfectly suitable for a study if the only data utilized were diagnosis records to help with cohort identification and recruitment. A wholesale deletion of records or imputation of values can obfuscate issues that might otherwise be disqualifying.

In evaluating specific data check failures, there are several reasons why a given DataMart may have exceptions. In many cases, it is because the issue is essentially “irremediable” or unresolvable for that DataMart. Examples would be those that are due to practice variation or care settings (e.g., missing inpatient visits in an ambulatory care center), population characteristics (i.e., variation in normal blood pressure ranges between pediatric and adult populations), or missing data that results from source system limitations (e.g., discharge status not recorded in the EHR if the patient is discharged alive, billing systems that record procedure dates before or after the corresponding encounter dates in the EHR). Other exceptions result from factors that could potentially be improved, such as errors in mapping records to a reference terminology (e.g., LOINC), missing metadata that is not in the EHR or research data warehouse but may exist in ancillary systems (e.g., lab reference ranges), or issues introduced by the extract-transform-load process used to populate the CDM. Unfortunately, it can be difficult to say categorically that all of the exceptions for a given check are due to one set of factors. Given the heterogeneity of PCORnet network partners, a given metric that is remediable for one partner may not be for another, and “remediability” may change over time (e.g., an issue can be resolved after a source system upgrade). This variation is one of the reasons why there are so few required data checks within the foundational curation process. To help PCORnet network partners learn from one another, the network has recently implemented the concept of a Data Quality Collaborative, to allow for more sharing of best practices to address issues in the foundational curation process as well as any issues uncovered through study-specific data curation.

The attention of PCORnet partners has been focused primarily on those metrics that are generally modifiable. These metrics also tend to be the ones that are tied to contract (funding) milestones for network partners. We have seen clear improvement in mapping to reference terminologies. Laboratory results and medication orders have not traditionally been captured in source systems using the formats mandated for interoperability (e.g., LOINC, RxNorm), so we designed several checks that would allow us to assess the assignment of these mappings over time, and we have seen improvement between Cycles (Table 4). The percentages for some of the other lab-related checks are lower because they rely on data elements that may not be present in the EHR or research data warehouse used to populate the CDM (e.g., specimen source, result unit, reference range). However, these elements are important for verifying that a correct LOINC code was assigned to the lab and/or using the records in an analysis (i.e., result unit), so we continue to work with network partners to improve performance.

A number of data checks have essentially stable performance and/or they plateau after a period of initial improvement. Many of the plausibility checks fall into this category. Even if the results for a data check cannot be improved further, reporting on the measure can influence a study design before the project starts (e.g., consider a different approach or supplemental data source(s) or determine whether a site needs to be excluded from an analysis). The data check that looks at Principal Diagnosis (DC2.07) is such an example. Principal Diagnosis is an important concept in many analyses that utilize administrative claims data, and it is present in some EHRs, but not all. Having a check that focuses on this concept ensures study teams are aware that they may not be able to rely on that data element when working with certain network partners. The ADAPTABLE study team originally planned to rely on that concept for some of their analyses, but had to modify their protocols due to the relatively high degree of missingness for Principal Diagnosis across the network.

The findings from projects, in terms of study-specific data quality investigations, are also key to the functioning of the network, allowing results to inform the foundational data curation process and improve overall network quality. For example, the PCORnet Antibiotic Study [20–22], which used medication orders as part of its analysis, issued its first SSDC query before the PRESCRIBING table had been fully curated. Key medications of interest appeared to be missing in the PCORnet® CDM because of how network partners had mapped their data to RxNorm. These findings directly influenced the design of DC3.08, which defined a set of preferred term types. Almost every study had faced issues with partners dropping records between refreshes, which is why the persistence checks were added in Cycle 6. While there are sometimes reasons why a network partner may have issues across multiple refreshes (e.g., migrating EHRs, adding or removing hospitals and clinics), others are simply due to the operational nature of the EHR and reflect underlying system changes, making it difficult to predict whether any given network partner may have an issue. The DC2.08 (monthly outliers) check was created due the discovery of a site dropping all records for a single quarter within their study extract. Another example of a data check informed from study experiences is DC3.12 (lab units). Laboratory results that lack units of measure cannot be used for queries that rely on laboratory cut-off values (e.g., stratifying glucose levels at different levels depending on whether the results are measured in mg/dL or mmol/L). Within ADAPTABLE [23–26], the study team needed a way of determining the completeness of various PCORnet® CDM tables (i.e., if the PCORnet® CDM was refreshed in March, what is the most recent month with “complete” data?), which led to the creation of the latency checks DC3.07, DC3.11 and DC3.14.

Latency can be a tricky concept within DRNs like PCORnet. Since data are only refreshed once a quarter, there will always be a lag. The purpose of the PCORnet latency checks has been to move the network so as much recent data are included as close to the refresh date as possible. A more frequent refresh cadence is possible, such as during the COVID-19 pandemic, when a subset of the PCORnet® CDM was refreshed weekly and then monthly. During this time, only a subset of data curation process was utilized. With the entire process as it currently exists, there would eventually be a point where the process to curate the data takes longer than the window until the next refresh. Given that most observational and retrospective research projects do not need data in real-time, the network has opted to maintain a quarterly refresh schedule to balance costs. Studies that need more real-time information, such as recent laboratory results to screen patients for recruitment, often employ a hybrid approach, using the PCORnet® CDM to for some variables, with a handful of other variables extracted directly from the EHR.

In deciding which study findings to prioritize as data checks, we have focused on those that are cross-cutting, or likely to affect projects across multiple domains. Anecdotally, teams that have conducted multiple studies leveraging PCORnet report that the implementation of these new checks have resolved the issue that was uncovered or at least allowed them to select a different site for participation. Network partners that receive infrastructure funding to participate in PCORnet have expectations within their contract milestones from PCORI to report on progress towards addressing data quality issues, so another factor that affects data check selection is whether there are reasonable expectations that issues can be addressed. Other data checks are based on the expansion of the PCORnet® CDM, which has historically been based in part on priorities of funders like PCORI.

The human resources required to develop and maintain the PCORnet® CDM and data curation processes across the Coordinating Center has been relatively stable across the years, designed and maintained by a dedicated team of project leaders, programmers and informatics analysts. Responsibilities include development of specifications and programs, testing, and working with network partners to resolve issues and gather feedback. When new network partners are onboarded as participants in PCORnet, it generally takes them a couple of curation cycles to resolve major issues and stabilize the process. Network partners that tend to consistently have more issues (e.g., a higher than median number of data check exceptions) tend to be those who participate in fewer research initiatives or lack dedicated staff for producing EHR extracts for research purposes. Even so, as more academic medical centers become proficient with common data models and data harmonization, and as EHR vendors standardize their internal data warehouses and reporting databases, it has made the process of implementing the PCORnet® CDM more straightforward.

## Conclusion

The design of the PCORnet® CDM and iterative data curation process have allowed the PCORnet network to assess the state of the underlying data in the source systems of network partners. While there are still areas that need attention, the overall quality of the data in the network has improved, increasing its research readiness over time. The quality issues that exist within the network are not inherent to PCORnet or the PCORnet® CDM, but stem from the way that data are captured within the EHR across healthcare generally. We have been able to make great strides, and given the intense focus on real-world evidence, personalized medicine, and value-based care, we believe that the industry as a whole will begin to push for more quality in the collection of data in the EHR. Many of the techniques piloted within PCORnet will be broadly applicable to those efforts. These include the structure and value sets used within the PCORnet® CDM, the data check definitions, data curation package routines, and the format and structure of the EDC report that is used to convey findings to users.

There has not yet been an effort to harmonize data quality checks across distributed networks, though there have been comparisons of data checks across networks and they do leverage the learnings from one another [19,27,28]. Since networks may be focused on different uses cases (i.e., cohort recruitment vs. retrospective pharmacoepidemiology studies vs. prospective pragmatic trials) [16,29–33], they may have drastically different quality requirements. In general, if a partner participates in multiple networks, passing the checks of one network tends to improve their ability to pass the checks of another. As the FDA produces guidance on the use of RWD and the corpus of RWE studies grows, this should help the field achieve some level of agreement on the numbers/types of checks that are sufficient for determining if data are fit for use.

## Supporting information

10.1017/cts.2025.10207.sm001 Marsolo et al. supplementary material 1 Marsolo et al. supplementary material

10.1017/cts.2025.10207.sm002 Marsolo et al. supplementary material 2 Marsolo et al. supplementary material

## Data Availability

Data metrics reported in this manuscript are available from the authors upon request.

## Supplementary material

The supplementary material for this article can be found at https://doi.org/10.1017/cts.2025.10207.
