# Supplementary material for: Assessing and improving research readiness in PCORnet®
Source: J Clin Transl Sci. 2025 Dec 17;9(1):e279. doi: 10.1017/cts.2025.10207 (PMC12722095; doi:10.1017/cts.2025.10207)
Supplement: Marsolo et al. supplementary material 1 — Marsolo et al. supplementary material [file S2059866125102070sup001.pdf]

PCORnet® Empirical Data Curation Report Table of Contents

This report is derived from the results of the data curation query. This query package fully characterizes all CDM v6.1 tables.
Report Run Date: 2025-01-01
Query Run Date: 2025-01-01
Maximum Table Refresh Date: 2024-12-31
Query Package: DC V6.17
Lookback Date: 2019-01-01
CDM Version: 061
SAS_ETS licensed: Yes / SAS_ETS installed: Yes

Section	Table	Table Description	Data Check(s)
Data Check Summary	DC Summary	Data Check Exception Summary	n/a
Section I: Descriptive Information	Table IA	Demographic Summary	n/a
	Table IB	Potential Pools of Patients	2.09, 3.04, 3.05
	Table IC	Height, Weight, and Body Mass Index (BMI)	n/a
	Table ID	Records, Refresh Dates, Patients, Encounters, and Date Ranges by Table	1.18
	Table IE	Records Per Table by Encounter Type	n/a
	Table IF	Date Obfuscation or Imputation	n/a
	Chart IA	Trend in Vital Measures by Measurement Date, Past 5 Years	n/a
	Chart IB	Trend in Encounters by Admit Date and Encounter Type, Past 5 Years	n/a
	Chart IC	Trend in Institutional Encounters by Discharge Date and Encounter Type, Past 5 Years	n/a
	Chart ID	Trend in Laboratory Results by Result Date, Past 5 Years	n/a
	Chart IE	Trend in Prescribed Medications by Rx Order Date, Past 5 Years	n/a
	Chart IF	Trend in Dispensed Medications by Dispense Date, Past 5 Years	n/a
	Chart IG	Trend in Administered Medications by Start Date, Past 5 Years	n/a
	Chart IH	Trend in Condition Records by Report Date, Past 5 Years	n/a
	Chart II	Trend in Death Records by Death Date and Source, Past 5 Years	n/a
	Chart IJ	Trend in Immunization Records by Vx Record Date, Past 5 Years	n/a
	Chart IK	Trend in Clinical Observation Records by Start Date, Past 5 Years	n/a
	Chart IL	Trend in General Observation Records by Start Date, Past 5 Years	n/a

Sample data

REPORT_RUN_DATE is the date the EDC report was run. QUERY_RUN_DATE is the date the data curation query (Part 1 portion if running the program in parts) was run. MAXIMUM_TABLE_REFRESH_DATE is derived from the HARVEST table. The LOOKBACK_DATE is the earliest date eligible for inclusion in the query results (see the Work Plan for details). CDM_VERSION is from the HARVEST table. The SAS_ETS module is used for Data Check 2.08.

PCORnet Empirical Data Curation Report Table of Contents (continued - page 2 of 3)

This report is derived from the results of the data curation query. This query package fully characterizes all CDM v6.1 tables. Please refer to the PCORnet Empirical Data Curation Report v6.15 Specifications for additional details about this report.

Section	Table	Table Description	Data Check(s)
Section II: Data Model Conformance	Table IIA	Primary Key Errors	1.05
	Table IIB	Values Outside of Common Data Model (CDM) Specifications	1.06
	Table IIC	Non-Permissible Missing Values	1.07
	Table IID	Diagnostic Errors	1.01, 1.02, 1.03, 1.04, 1.17
	Table IIE	Orphan Records, Replication Errors, Encounter Duplication and Hash Token Duplication	1.08, 1.09,1.10, 1.11, 1.12, 1.14, 1.15, 1.19
	Table IIF	Potential Code Errors and Misplaced Codes	1.13, 1.16
	Table IIG	LOINC Panel Codes	1.20
Section III: Data Plausibility	Table IIIA	Future Dates	2.01
	Table IIIB	Records with Extreme Values	2.02
	Table IIIC	Illogical Dates	2.03
	Table IIID	Encounters Per Visit and Per Patient	2.04
	Table IIIG	Monthly Record Volume Outliers, Selected Domains	2.08
	Chart IIIA	Monthly Record Volume Outliers, Encounters	2.08
	Chart IIIB	Monthly Record Volume Outliers, Diagnoses	2.08
	Chart IIIC	Monthly Record Volume Outliers, Procedures	2.08
	Chart IIID	Monthly Record Volume Outliers, Vitals	2.08
	Chart IIIE	Monthly Record Volume Outliers, Prescribing	2.08
	Chart IIIF	Monthly Record Volume Outliers, Labs	2.08
	Chart IIIG	Monthly Record Volume Outliers, Med Admin	2.08

Sample data

REPORT_RUN_DATE is the date the EDC report was run. QUERY_RUN_DATE is the date the data curation query (Part 1 portion if running the program in parts) was run. MAXIMUM_TABLE_REFRESH_DATE is derived from the HARVEST table. The LOOKBACK_DATE is the earliest date eligible for inclusion in the query results (see the Work Plan for details). CDM_VERSION is from the HARVEST table. The SAS_ETS module is used for Data Check 2.08.

PCORnet Empirical Data Curation Report Table of Contents (continued - page 3 of 3)

This report is derived from the results of the data curation query. This query package fully characterizes all CDM v6.1 tables. Please refer to the PCORnet Empirical Data Curation Report v6.15 Specifications for additional details about this report.

Section	Table	Table Description	Data Check(s)
Section IV: Data Completeness and Plausibility	Table IVA	Diagnosis Records Per Encounter, Overall and by Encounter Type	3.01
	Chart IVA	Diagnosis Records Per Encounter by Admit Date and Encounter Type, Past 5 Years	n/a
	Table IVB	Procedure Records Per Encounter, Overall and by Encounter Type	3.02
	Chart IVB	Procedure Records Per Encounter by Admit Date and Encounter Type, Past 5 Years	n/a
	Table IVC	Missing or Unknown Values, Required Tables	3.03
	Table IVD	Missing or Unknown Values, Optional Tables	3.03
	Table IVE	Principal Diagnoses for Institutional Encounters	2.07, 3.06
	Table IVF	Data Latency and Completeness of Encounter, Diagnosis and Procedure Data, Past 2 Years	3.07
	Table IVG	Data Latency and Completeness of Vital, Prescription, and Lab Data, Past 2 Years	3.11
	Table IVH	RXNORM Term Type Mapping	3.08, 3.15
	Table IVI	Laboratory and Clinical Observation Result Data Completeness	3.09, 3.10, 3.12, 3.16, 3.17
	Table IVI_Ref	Laboratory and Clinical Observation Result Data Completeness Definitions	n/a
	Table IVJ	Data Latency and Completeness of Medication Administration, Dispensing and Clinical Observation Data, Past 2 Years	3.14
Section V: Data Persistence	Table VA	Changes in Tables	4.01
	Table VB	Changes in Selected Encounter Types and Domains	4.02
	Table VC	Changes in Selected Code Types	4.03

Sample data

REPORT_RUN_DATE is the date the EDC report was run. QUERY_RUN_DATE is the date the data curation query (Part 1 portion if running the program in parts) was run. MAXIMUM_TABLE_REFRESH_DATE is derived from the HARVEST table. The LOOKBACK_DATE is the earliest date eligible for inclusion in the query results (see the Work Plan for details). CDM_VERSION is from the HARVEST table. The SAS_ETS module is used for Data Check 2.08.

Data Check Exception Summary

This table summarizes conformance with PCORnet Data Checks v16. Changes to the previous cycle's data checks are explained in the footnotes. Exceptions to required data checks are highlighted in red and must be corrected before returning results. Exceptions to investigative data checks are highlighted in blue and must be investigated and explained in the ETL ADD.

Data Check	EDC Table	Data Check Description	Category	Type	Exception?	% DataMarts w/ exceptions
DC 1.01[a]	Table IID	Required tables are not present	Data Model Conformance	Required	No	---
DC 1.02[a]	Table IID	Required tables are not populated	Data Model Conformance	Required	**Yes**	---
DC 1.03[a]	Table IID	Required fields are not present	Data Model Conformance	Required	**Yes**	---
DC 1.04[a]	Table IID	Required fields do not conform to the data model specifications for data type, length, or name	Data Model Conformance	Required	**Yes**	---
DC 1.05	Table IIA	Tables have primary key definition errors	Data Model Conformance	Required	No	---
DC 1.06[b]	Table IIB	Required fields contain values outside of data model specifications	Data Model Conformance	Required	**Yes**	---
DC 1.07	Table IIC	Required fields have non-permissible missing values	Data Model Conformance	Required	No	---
DC 1.08	Table IIE	Tables contain orphan PATIDs	Data Model Conformance	Required	**Yes**	---
DC 1.09	Table IIE	Tables contain orphan ENCOUNTERIDs for more than 5% of records	Data Model Conformance	Required	**Yes**	---
DC 1.10	Table IIE	Replication errors between the ENCOUNTER, PROCEDURES and DIAGNOSIS tables	Data Model Conformance	Required	**Yes**	---
DC 1.11	Table IIE	More than 5% of encounters are assigned to more than one patient	Data Model Conformance	Required	**Yes**	---
DC 1.12	Table IIE	Tables contain orphan PROVIDERIDs	Data Model Conformance	Required	**Yes**	---
DC 1.13[c]	Table IIF	More than 5% of CPT/HCPCS, CVX, ICD, LOINC, NDC, or RXNORM codes do not conform to the expected length or content	Data Model Conformance	Required	**Yes**	---
DC 1.14	Table IIE	Patients in the DEMOGRAPHIC table are not in the HASH_TOKEN table	Data Model Conformance	Investigative	**Yes**	---
DC 1.15[c]	Table IID	Fields with undefined lengths that are present in more than one table do not have harmonized field lengths	Data Model Conformance	Required	**Yes**	---
DC 1.16	Table IIF	Laboratory results or clinical observations are recorded in the wrong table	Data Model Conformance	Investigative	No	---
DC 1.17	Table IID	Zip codes in the ENCOUNTER or LDS_ADDRESS_HISTORY table do not conform to expected values	Data Model Conformance	Required	**Yes**	---
DC 1.18	Table ID	Table refresh dates are not documented	Data Model Conformance	Required	No	---

Sample data

(a) Can be detected by CDM Diagnostic Query.
(b) Can be detected by the Value Set Conformance Query
(c) Can be detected by the Potential Code Errors Query
(d) New
(e) Added MED_ADMIN
(f) Changed from 3 months to 2 months

Data Check Exception Summary (continued - page 2 of 4)

This table summarizes conformance with PCORnet Data Checks v16. Changes to the previous cycle's data checks are explained in the footnotes. Exceptions to required data checks are highlighted in red and must be corrected before returning results. Exceptions to investigative data checks are highlighted in blue and must be investigated and explained in the ETL ADD.

Data Check	EDC Table	Data Check Description	Category	Type	Exception?	% DataMarts w/ exceptions
DC 1.19	Table IIE	More than 10% of hash tokens are assigned to multiple patients	Data Model Conformance	Investigative	**Yes**	---
DC 1.20[d]	Table IIG	More than 5% of LOINC records in the LAB_RESULT_CM, PRO_CM, and OBS_CLIN tables are panel codes	Data Model Conformance	Investigative	**Yes**	---
DC 2.01	Table IIIA	More than 5% of records have future dates	Data Plausibility	Investigative	**Yes**	---
DC 2.02	Table IIIB	More than 10% of records fall into the lowest or highest categories of age, height, weight, diastolic blood pressure, systolic blood pressure or dispensed days supply	Data Plausibility	Investigative	**Yes**	---
DC 2.03	Table IIIC	More than 5% of patients have illogical date relationships	Data Plausibility	Investigative	**Yes**	---
DC 2.04	Table IIID	The average number of encounters per visit is > 2.0 for inpatient (IP), emergency department (ED), or ED to inpatient (EI) encounters	Data Plausibility	Investigative	No	---
DC 2.07	Table IVE	The average number of principal diagnoses per known DX_ORIGIN per encounter is above threshold [2.0 for inpatient (IP) and ED to inpatient (EI)]	Data Plausibility	Investigative	No	---
DC 2.08[e]	Table IIIG	The monthly volume of encounter, diagnosis, procedure, vital, prescribing, medication administration or laboratory records is an outlier.	Data Plausibility	Investigative	No	---
DC 2.09	Table IB	Less than 80% of patients with a face-to-face encounter during the past 5 years have at least 1 face-to-face diagnosis and 1 vital measurement.	Data Plausibility	Investigative	**Yes**	---
DC 3.01	Table IVA	The average number of diagnoses records with known diagnosis types per encounter is below threshold [1.0 for ambulatory (AV), inpatient (IP), emergency department (ED), ED to inpatient (EI), or telehealth (TH) encounters].	Data Completeness	Investigative	**Yes**	---
DC 3.02	Table IVB	The average number of procedure records with known procedure types per encounter is below threshold [0.75 for ambulatory (AV) encounters, 0.75 for emergency department (ED) encounters, 1.00 for ED to inpatient (EI) encounters, and 1.00 for inpatient (IP) encounters].	Data Completeness	Investigative	No	---

Sample data

(a) Can be detected by CDM Diagnostic Query.
(b) Can be detected by the Value Set Conformance Query
(c) Can be detected by the Potential Code Errors Query
(d) New
(e) Added MED_ADMIN
(f) Changed from 3 months to 2 months

Data Check Exception Summary (continued - page 3 of 4)

This table summarizes conformance with PCORnet Data Checks v16. Changes to the previous cycle's data checks are explained in the footnotes. Exceptions to required data checks are highlighted in red and must be corrected before returning results. Exceptions to investigative data checks are highlighted in blue and must be investigated and explained in the ETL ADD.

Data Check	EDC Table	Data Check Description	Category	Type	Exception?	% DataMarts w/ exceptions
DC 3.03	Table IVC	More than 10% of records have missing or unknown values for the following fields: DISCHARGE_DISPOSITION (IP/EI encounters only), DISPENSE_SUP, DX_SOURCE, SEX, code fields [DEATH_CAUSE_CODE, MEDADMIN_CODE, OBSCLIN_CODE, OBSGEN_CODE], code type fields [CONDITION_TYPE, DX_TYPE, MEDADMIN_TYPE, OBSCLIN_TYPE, OBSGEN_TYPE, PX_TYPE, VX_CODE_TYPE], date fields [BIRTH_DATE, DISCHARGE_DATE (IP/EI encounters only), RX_ORDER_DATE, PX_DATE, VX_RECORD_DATE], ENCOUNTERID fields in selected tables [DIAGNOSIS, LAB_RESULT_CM, PRESCRIBING, MED_ADMIN, OBS_CLIN, PROCEDURES, and VITAL], and provenance fields [CONDITION_SOURCE, DEATH_CAUSE_SOURCE, DEATH_SOURCE, DISPENSE_SOURCE, DX_ORIGIN, MEDADMIN_SOURCE, LAB_RESULT_SOURCE, PX_SOURCE, RX_SOURCE, VITAL_SOURCE, VX_SOURCE]	Data Completeness	Investigative	**Yes**	---
DC 3.03	Table IVD	See Table IVC	Data Completeness	Investigative	**Yes**	---
DC 3.04	Table IB	Less than 50% of patients with encounters have DIAGNOSIS records	Data Completeness	Required	No	---
DC 3.05	Table IB	Less than 50% of patients with encounters have PROCEDURES records	Data Completeness	Required	No	---
DC 3.06	Table IVE	More than 10% of IP (inpatient) or ED to inpatient (EI) encounters with any diagnosis from a known DX_ORIGIN don't have a principal diagnosis from that source	Data Completeness	Investigative	**Yes**	---
DC 3.07[f]	Table IVF	Encounters, diagnoses or procedures in an ambulatory (AV), telehealth (TH), emergency department (ED), ED to inpatient (EI), or inpatient (IP) setting are less than 75% complete two months prior to the current month	Data Completeness	Investigative	**Yes**	---
DC 3.08	Table IVH	Less than 80% of prescribing orders are mapped to a RXNORM_CUI which fully specifies the ingredient, strength and dose form	Data Completeness	Investigative	**Yes**	---

(a) Can be detected by CDM Diagnostic Query.
(b) Can be detected by the Value Set Conformance Query
(c) Can be detected by the Potential Code Errors Query
(d) New
(e) Added MED_ADMIN
(f) Changed from 3 months to 2 months

Sample data

Data Check Exception Summary (continued - page 4 of 4)

This table summarizes conformance with PCORnet Data Checks v16. Changes to the previous cycle's data checks are explained in the footnotes. Exceptions to required data checks are highlighted in red and must be corrected before returning results. Exceptions to investigative data checks are highlighted in blue and must be investigated and explained in the ETL ADD.

Data Check	EDC Table	Data Check Description	Category	Type	Exception?	% DataMarts w/ exceptions
DC 3.09	Table IVI	Less than 80% of laboratory results are mapped to LAB_LOINC and have either a quantitative or qualitative result	Data Completeness	Investigative	No	---
DC 3.10	Table IVI	Less than 80% of quantitative results for tests mapped to LAB_LOINC fully specify the normal range	Data Completeness	Investigative	**Yes**	---
DC 3.11	Table IVG	Vital, prescribing, or laboratory records are less than 75% complete three months prior to the current month.	Data Completeness	Investigative	**Yes**	---
DC 3.12	Table IVI	Less than 80% of quantitative results for tests mapped to LAB_LOINC fully specify the result unit	Data Completeness	Investigative	**Yes**	---
DC 3.14	Table IVJ	Medication administration, dispensing, or clinical observation records are less than 75% complete three months prior to the current month	Data Completeness	Investigative	**Yes**	---
DC 3.15	Table IVH	Less than 80% of medication administrations mapped to RXNORM are mapped to a RXNORM_CUI that fully specifies the ingredient, strength and dose form.	Data Completeness	Investigative	**Yes**	---
DC 3.16	Table IVI	Less than 80% of clinical observations are mapped to an OBSCLIN_CODE and have a quantitative, qualitative or narrative result	Data Completeness	Investigative	**Yes**	---
DC 3.17	Table IVI	Less than 80% of quantitative results for tests mapped to OBSCLIN_CODE fully specify the RESULT_UNIT	Data Completeness	Investigative	**Yes**	---
DC 4.01	Table VA	More than a 5% decrease in the number of patients or records in a CDM table	Data Persistence	Investigative	**Yes**	---
DC 4.02	Table VB	More than a 5% decrease in the number of patients or records for diagnosis, procedures, labs or prescriptions during an ambulatory (AV), telehealth (TH), other ambulatory (OA), emergency department (ED), or inpatient (IP) encounter.	Data Persistence	Investigative	**Yes**	---
DC 4.03	Table VC	More than a 5% decrease in the number of records or distinct codes for CPT/HCPCS, CVX, ICD10, NDC, or RXNORM codes.	Data Persistence	Investigative	**Yes**	---

Sample data

(a) Can be detected by CDM Diagnostic Query.
(b) Can be detected by the Value Set Conformance Query
(c) Can be detected by the Potential Code Errors Query
(d) New
(e) Added MED_ADMIN
(f) Changed from 3 months to 2 months

Table IA. Demographic Summary

This table contains general descriptive information about the patients in the DEMOGRAPHIC table. These patients may or may not be represented in other CDM tables.

	N	%	Source table
Patients	3,644		DEM_L3_N
Age			DEM_L3_AGEYRSDIST1
Mean	36		
Median	24		
Age group			DEM_L3_AGEYRSDIST2
0-4	103	2.8	
5-14	43	1.2	
15-21	718	19.7	
22-64	2,581	70.8	
65+	199	5.5	
Missing	0	0.0	
Hispanic			DEM_L3_HISPDIST
N (No)	1,597	43.8	
Y (Yes)	1,626	44.6	
Missing or Refused	421	11.6	
Sex			DEM_L3_SEXDIST
F (Female)	1,297	35.6	
M (Male)	1,250	34.3	
Missing or Ambiguous	1,097	30.1	
Race			DEM_L3_RACEDIST
White	460	12.6	
Non-White	2,331	63.9	
Missing or Refused	853	23.5	

Sample data

The four 'flavors of null' defined in the CDM are combined in the missing categories shown here, but details are available in the source tables.
Age is calculated as current age or age at death if death date is known. If multiple death records exist, the earlier death date is used.
Percentages are sums of the percentages in the source table and are subject to rounding errors.
Race distribution is shown for all patients and for patients seen after Meaningful Use standards were widely implemented.

Table IA. Demographic Summary (continued)

This table contains general descriptive information about the patients in the DEMOGRAPHIC table. These patients may or may not be represented in other CDM tables.

	N	%	Source table
Race among patients with at least 1 encounter after December 2011			XTBL_L3_RACE_ENC
White	371	12.4	
Non-White	1,911	64.1	
Missing or Refused	699	23.4	
Gender Identity			DEM_L3_GENDERDIST
GQ (Genderqueer/Non-Binary)	348	9.5	
M (Man)	333	9.1	
W (Woman)	339	9.3	
MU (Multiple gender categories), SE (Something else), TF (Transgender female/Trans woman/Male-to-female), or TM (Transgender male/Trans man/Female-to-male)	1,377	37.8	
Missing or Refused	1,247	34.2	
Sexual Orientation			DEM_L3_ORIENTDIST
Bisexual	298	8.2	
Gay	285	7.8	
Lesbian	340	9.3	
Queer	317	8.7	
Straight	273	7.5	
AS (Asexual), MU (Multiple sexual orientations), SE (Something else), QS (Questioning)	1,304	35.7	
Missing or Refused	461	12.8	

Sample data

The four 'flavors of null' defined in the CDM are combined in the missing categories shown here, but details are available in the source tables.
Age is calculated as current age or age at death if death date is known. If multiple death records exist, the earlier death date is used.
Percentages are sums of the percentages in the source table and are subject to rounding errors.
Race distribution is shown for all patients and for patients seen after Meaningful Use standards were widely implemented.

Table IB. Potential Pools of Patients

This table illustrates the number of patients meeting different inclusion criteria and supports Data Check 2.09 (Less than 80% of patients with a face-to-face encounter during the past 5 years have at least 1 face-to-face diagnosis and 1 vital measurement), Data Check 3.04 (less than 50% of patients with encounters have DIAGNOSIS records) and Data Check 3.05 (less than 50% of patients with encounters have PROCEDURES records). Data check exceptions to 3.04 and 3.05 are highlighted in red and must be corrected; data check exceptions to 2.09 are highlighted in blue and must be explained in the ETL ADD.

Metric	Metric Description	Result	%	Source table
All patients	Number of unique patients in the DEMOGRAPHIC table	3,644		DEM_L3_N
Potential pool of patients for observational studies	Number of unique patients with at least 1 face-to-face (ED, EI, IP, OS, or AV) encounter within the past 5 years	3,689	101	ENC_L3_DASH2
Potential pool of patients for trials	Number of unique patients with at least 1 face-to-face (ED, EI, IP, OS, or AV) encounter within the past 1 year	2,298	63	ENC_L3_DASH2
Potential pool of patients for studies requiring data on diagnoses, vital measures and (a) medications or (b) medications and lab results	Number of unique patients with at least 1 DIAGNOSIS record in a face-to-face setting and at least 1 VITAL record within the past 5 years	1,202	**32.6**	XTBL_L3_DASH1
	Number of unique patients with at least 1 DIAGNOSIS record in a face-to-face setting, at least 1 VITAL record, and at least 1 PRESCRIBING or MED_ADMIN record within the past 5 years	1,100	29.8	XTBL_L3_DASH2
	Number of unique patients with at least 1 DIAGNOSIS record in a face-to-face setting, at least 1 VITAL record, at least 1 PRESCRIBING or MED_ADMIN record, and at least 1 LAB_RESULT_CM record within the past 5 years	924	25.0	XTBL_L3_DASH3
Patients with diagnosis data	Percentage of patients with encounters who have at least 1 diagnosis	78%		ENC_L3_N; DIA_L3_N
Patients with procedure data	Percentage of patients with encounters who have at least 1 procedure	99%		ENC_L3_N; PRO_L3_N

Sample data

Table IC. Height, Weight, and Body Mass Index

This table contains descriptive statistics and frequencies of VITAL measurements.

	Result	%	Source table
Height measurements			VIT_L3_HT_DIST
Records	3,686		
Height (inches), mean	65		
Height (inches), median	65		
Weight measurements			VIT_L3_WT_DIST
Records	3,714		
Weight (lbs.), mean	219		
Weight (lbs.), median	173		
Body Mass Index (BMI) measurements			VIT_L3_BMI
Records	2,768		
BMI <=25	1,495	54.0	
BMI 26-30	347	12.5	
BMI >=31	926	33.5	

Sample data

Table ID. Records, Refresh Dates, Patients, Encounters, and Date Ranges by Table

This table displays record counts and refresh dates for all populated tables, and summary counts and date ranges for the characterized data. The characterized data may be a subset of all data for all tables except for DEMOGRAPHIC, DEATH_CAUSE, HASH_TOKEN, LAB_HISTORY, LDS_ADDRESS_HISTORY, and PROVIDER. Differences are due to the lookback date restriction and/or the exclusion of LOINC codes that may contain PII from the OBS_CLIN, OBS_GEN, LAB_RESULT_CM and PRO_CM tables. These data support Data Check 1.18 (table refresh dates are not documented). Data check exceptions are highlighted in red and must be corrected.

	All Data		Characterized Data			Characterized Data Range			
Table	Records	Refresh Date	Records	Patients	Encounters	Field name	5th Percentile	95th Percentile	Source Tables
DEMOGRAPHIC	3,644	31DEC2024	3,644	3,644	---	BIRTH_DATE	1942_Jan	2003_Dec	DEM_L3_N; XTBL_L3_DATES
ENCOUNTER	107,044	31DEC2024	107,044	4,039	107,044	ADMIT_DATE	2021_Feb	2024_Aug	ENC_L3_N; XTBL_L3_DATES
DIAGNOSIS	32,031	31DEC2024	32,031	3,138	32,031	ADMIT_DATE	2021_Feb	2024_Jun	DIA_L3_N; XTBL_L3_DATES
PROCEDURES	186,105	31DEC2024	186,105	4,005	186,103	ADMIT_DATE	2021_Mar	2025_Dec	PRO_L3_N; XTBL_L3_DATES
DEATH	1,216	30JUN2024	1,216	1,216	---	DEATH_DATE	2021_Feb	2024_Feb	DEATH_L3_N; XTBL_L3_DATES
VITAL	3,765	31DEC2024	3,765	2,299	2	MEASURE_DATE	2021_Feb	2024_Aug	VIT_L3_N; XTBL_L3_DATES
LAB_RESULT_CM	10,508	31DEC2024	10,508	4,330	0	RESULT_DATE	2021_Feb	2024_Sep	LAB_L3_N; XTBL_L3_DATES
PRESCRIBING	7,574	31DEC2024	7,574	3,365	3,767	RX_ORDER_DATE	2021_Feb	2024_Aug	PRES_L3_N; XTBL_L3_DATES

Sample data

^The lookback date for ENROLLMENT considers either ENR_START_DATE or ENR_END_DATE, but only ENR_START_DATE is displayed here. Descriptive statistics for ENR_END_DATE are available in the XTBL_L3_DATES dataset.

In the All Data columns, records comes from the DATAMART_ALL table and Refresh Date comes from the XTBL_L3_REFRESH_DATE table.

Records are the sum of ALL_N and NULL_N for the field which is the primary key for the table. Patients are the sum of DISTINCT_N and NULL_N for the PATID field. Encounters are the sum of DISTINCT_N and NULL_N for the ENCOUNTERID field.

Table ID. Records, Refresh Dates, Patients, Encounters, and Date Ranges by Table (continued - page 2 of 3)

This table displays record counts and refresh dates for all populated tables, and summary counts and date ranges for the characterized data. The characterized data may be a subset of all data for all tables except for DEMOGRAPHIC, DEATH_CAUSE, HASH_TOKEN, LAB_HISTORY, LDS_ADDRESS_HISTORY, and PROVIDER. Differences are due to the lookback date restriction and/or the exclusion of LOINC codes that may contain PII from the OBS_CLIN, OBS_GEN, LAB_RESULT_CM and PRO_CM tables. These data support Data Check 1.18 (table refresh dates are not documented). Data check exceptions are highlighted in red and must be corrected.

	All Data		Characterized Data			Characterized Data Range			
Table	Records	Refresh Date	Records	Patients	Encounters	Field name	5th Percentile	95th Percentile	Source Tables
DISPENSING	12,323	30JUN2024	12,323	4,557	---	DISPENSE_DATE	2021_Feb	2024_Mar	DISP_L3_N; XTBL_L3_DATES
PROVIDER	10,670	31DEC2024	10,670	---	---	---	---	---	PROV_L3_N
MED_ADMIN	4,349	31DEC2024	4,349	4,076	3,382	MEDADMIN_ START_DATE	2021_Feb	2024_Sep	MEDADM_L3_N; XTBL_L3_DATES
OBS_CLIN	3,354	31DEC2024	3,354	3,192	0	OBSCLIN_START_ DATE	2021_Mar	2024_Aug	OBSCLIN_L3_N; XTBL_L3_DATES

Sample data

^The lookback date for ENROLLMENT considers either ENR_START_DATE or ENR_END_DATE, but only ENR_START_DATE is displayed here. Descriptive statistics for ENR_END_DATE are available in the XTBL_L3_DATES dataset.

In the All Data columns, records comes from the DATAMART_ALL table and Refresh Date comes from the XTBL_L3_REFRESH_DATE table.

Records are the sum of ALL_N and NULL_N for the field which is the primary key for the table. Patients are the sum of DISTINCT_N and NULL_N for the PATID field. Encounters are the sum of DISTINCT_N and NULL_N for the ENCOUNTERID field.

Table ID. Records, Refresh Dates, Patients, Encounters, and Date Ranges by Table (continued - page 3 of 3)

This table displays record counts and refresh dates for all populated tables, and summary counts and date ranges for the characterized data. The characterized data may be a subset of all data for all tables except for DEMOGRAPHIC, DEATH_CAUSE, HASH_TOKEN, LAB_HISTORY, LDS_ADDRESS_HISTORY, and PROVIDER. Differences are due to the lookback date restriction and/or the exclusion of LOINC codes that may contain PII from the OBS_CLIN, OBS_GEN, LAB_RESULT_CM and PRO_CM tables. These data support Data Check 1.18 (table refresh dates are not documented). Data check exceptions are highlighted in red and must be corrected.

	All Data		Characterized Data			Characterized Data Range			
Table	Records	Refresh Date	Records	Patients	Encounters	Field name	5th Percentile	95th Percentile	Source Tables
HASH_TOKEN	5,720	31DEC2024	5,720	4,720	---	---	---	---	HASH_L3_N
IMMUNIZATION	166,567	30JUN2024	166,567	3,967	158,177	VX_RECORD_DATE	2021_Feb	2024_Mar	IMMUNE_L3_N; XTBL_L3_DATES

Sample data

^The lookback date for ENROLLMENT considers either ENR_START_DATE or ENR_END_DATE, but only ENR_START_DATE is displayed here. Descriptive statistics for ENR_END_DATE are available in the XTBL_L3_DATES dataset.

In the All Data columns, records comes from the DATAMART_ALL table and Refresh Date comes from the XTBL_L3_REFRESH_DATE table.

Records are the sum of ALL_N and NULL_N for the field which is the primary key for the table. Patients are the sum of DISTINCT_N and NULL_N for the PATID field. Encounters are the sum of DISTINCT_N and NULL_N for the ENCOUNTERID field.

Table IE. Records Per Table By Encounter Type

This table contains record counts by encounter type for the ENCOUNTER, DIAGNOSIS, and PROCEDURES tables.

	ENCOUNTER		DIAGNOSIS		PROCEDURES	
Encounter Type	N	%	N	%	N	%
AV (Ambulatory Visit)	10,682	10.0	3,077	9.6	18,114	9.7
ED (Emergency Dept)	10,649	9.9	3,146	9.8	18,156	9.8
EI (ED to IP Stay)	10,918	10.2	3,152	9.8	18,125	9.7
IC (Institutional Professional Consult)	10,734	10.0	3,087	9.6	18,135	9.7
IP (Inpatient Hospital Stay)	10,748	10.0	3,041	9.5	18,099	9.7
IS (Non-acute Institutional Stay)	10,731	10.0	3,213	10.0	18,295	9.8
OA (Other Ambulatory Visit)	10,691	10.0	3,066	9.6	18,124	9.7
OS (Observation Stay)	10,828	10.1	3,102	9.7	18,198	9.8
TH (Telehealth)	10,603	9.9	3,181	9.9	18,094	9.7
Missing, NI, UN or OT	10,460	9.8	3,966	12.4	22,765	12.2
Total	107,044		32,031		186,105	
Source table	ENC_L3_ENCTYPE		DIA_L3_ENCTYPE		PRO_L3_ENCTTPE	

Sample data

Table IF. Date Obfuscation or Imputation

This table contains information about the presence of date obfuscation or imputation. Imputed or obfuscated dates are important to consider when interpreting results. The use of date obfuscation is highlighted in orange and will be used to assess compliance with the terms of the CRN Scope of Work.

Table	Field	DATE_MGMT	Source table
HARVEST	BIRTH_DATE_MGMT	**03 (Date obfuscation)**	XTBL_L3_METADATA
HARVEST	ENR_START_DATE_MGMT	01 (No imputation or obfuscation)	XTBL_L3_METADATA
HARVEST	ENR_END_DATE_MGMT	01 (No imputation or obfuscation)	XTBL_L3_METADATA
HARVEST	ADMIT_DATE_MGMT	01 (No imputation or obfuscation)	XTBL_L3_METADATA
HARVEST	DISCHARGE_DATE_MGMT	01 (No imputation or obfuscation)	XTBL_L3_METADATA
HARVEST	PX_DATE_MGMT	01 (No imputation or obfuscation)	XTBL_L3_METADATA
HARVEST	RX_ORDER_DATE_MGMT	01 (No imputation or obfuscation)	XTBL_L3_METADATA
HARVEST	RX_START_DATE_MGMT	02 (Imputation for incomplete dates)	XTBL_L3_METADATA
HARVEST	RX_END_DATE_MGMT	02 (Imputation for incomplete dates)	XTBL_L3_METADATA
HARVEST	DISPENSE_DATE_MGMT	01 (No imputation or obfuscation)	XTBL_L3_METADATA
HARVEST	LAB_ORDER_DATE_MGMT	01 (No imputation or obfuscation)	XTBL_L3_METADATA
HARVEST	SPECIMEN_DATE_MGMT	01 (No imputation or obfuscation)	XTBL_L3_METADATA
HARVEST	RESULT_DATE_MGMT	01 (No imputation or obfuscation)	XTBL_L3_METADATA
HARVEST	MEASURE_DATE_MGMT	01 (No imputation or obfuscation)	XTBL_L3_METADATA
HARVEST	ONSET_DATE_MGMT	01 (No imputation or obfuscation)	XTBL_L3_METADATA
HARVEST	REPORT_DATE_MGMT	01 (No imputation or obfuscation)	XTBL_L3_METADATA
HARVEST	RESOLVE_DATE_MGMT	01 (No imputation or obfuscation)	XTBL_L3_METADATA
HARVEST	PRO_DATE_MGMT	01 (No imputation or obfuscation)	XTBL_L3_METADATA
HARVEST	DEATH_DATE_MGMT	01 (No imputation or obfuscation)	XTBL_L3_METADATA
HARVEST	MEDADMIN_START_DATE_MGMT	01 (No imputation or obfuscation)	XTBL_L3_METADATA
HARVEST	MEDADMIN_STOP_DATE_MGMT	01 (No imputation or obfuscation)	XTBL_L3_METADATA
HARVEST	OBSCLIN_START_DATE_MGMT	01 (No imputation or obfuscation)	XTBL_L3_METADATA
HARVEST	OBSCLIN_STOP_DATE_MGMT	01 (No imputation or obfuscation)	XTBL_L3_METADATA
HARVEST	OBSGEN_START_DATE_MGMT	01 (No imputation or obfuscation)	XTBL_L3_METADATA
HARVEST	OBSGEN_STOP_DATE_MGMT	01 (No imputation or obfuscation)	XTBL_L3_METADATA
HARVEST	DX_DATE_MGMT	01 (No imputation or obfuscation)	XTBL_L3_METADATA
HARVEST	ADDRESS_PERIOD_START_MGMT	01 (No imputation or obfuscation)	XTBL_L3_METADATA
HARVEST	ADDRESS_PERIOD_END_MGMT	01 (No imputation or obfuscation)	XTBL_L3_METADATA
HARVEST	VX_RECORD_DATE_MGMT	01 (No imputation or obfuscation)	XTBL_L3_METADATA
HARVEST	VX_ADMIN_DATE_MGMT	01 (No imputation or obfuscation)	XTBL_L3_METADATA
HARVEST	VX_EXP_DATE_MGMT	01 (No imputation or obfuscation)	XTBL_L3_METADATA

Sample data

Details regarding the type of obfuscation or imputation are available in the source data.

Chart IA. Trend In Vital Measures by Measurement Date, Past 5 Years

This chart illustrates relative changes over time in the number of records found in the VITAL table. For all charts, the X-axis is the 60 months prior to the maximum refresh date. Monthly record counts were standardized over the period shown to have a mean of 0 and a standard deviation of 1. The y-axis reflects the deviation in each month’s count from the mean. A value above 0 indicates an above-average number of records; a value below 0 indicates a below-average number of records. Significant inflection points and other unexpected patterns should be investigated.

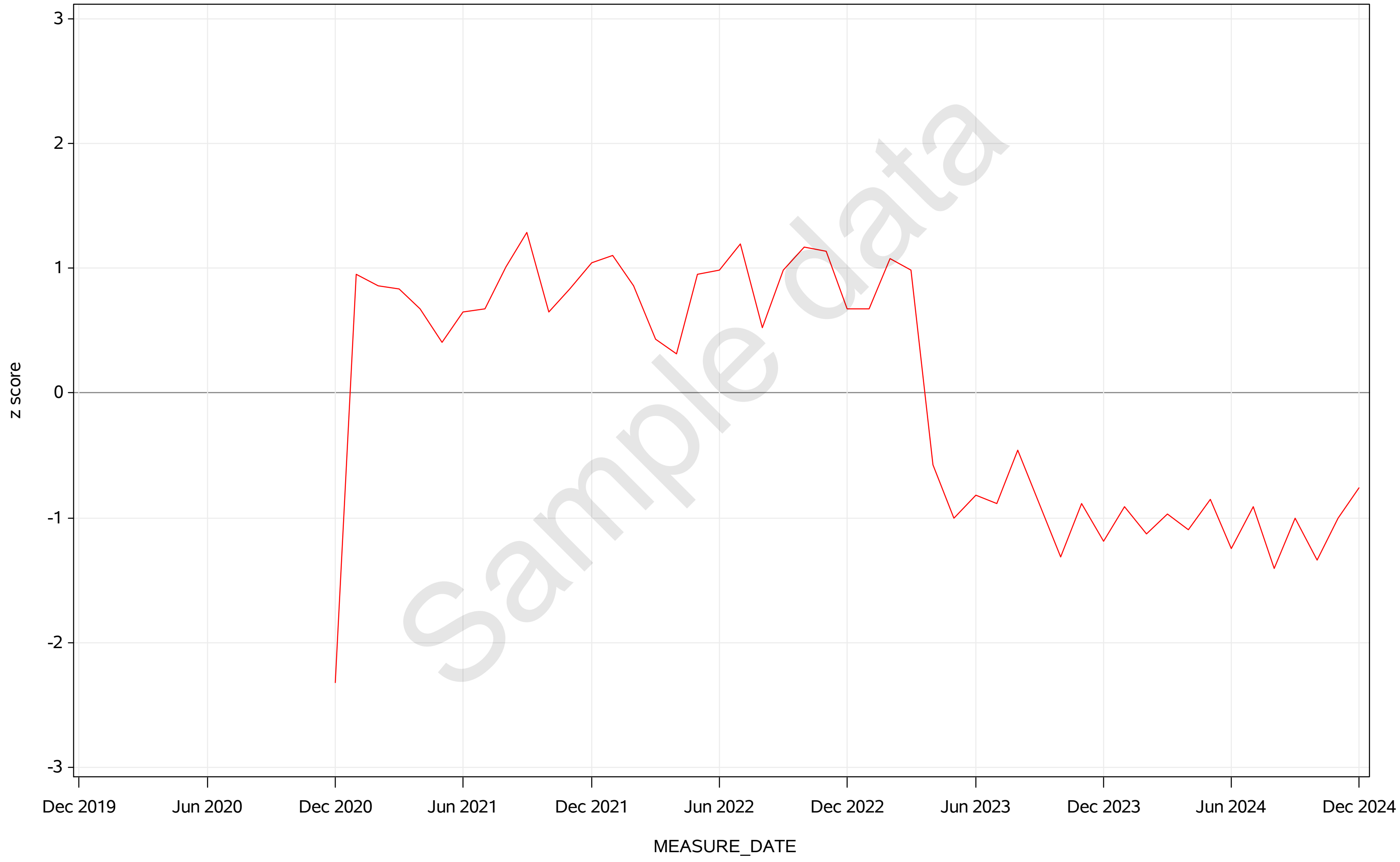

Chart IB. Trend in Encounters by Admit Date and Encounter Type, Past 5 Years

This 3 panel chart illustrates relative changes over time in the number of records per encounter type found in the ENCOUNTER table by admit date. The first panel includes AV, OA, and TH encounter types; the second panel includes ED, EI and IP encounter types, and the third panel includes IS, IC, and OS encounter types. For all charts, the X-axis is the 60 months prior to the maximum refresh date. Monthly record counts were standardized over the period shown to have a mean of 0 and a standard deviation of 1. The y-axis reflects the deviation in each month's count from the mean. A value above 0 indicates an above-average number of records; a value below 0 indicates a below-average number of records. Significant inflection points and other unexpected patterns should be investigated.

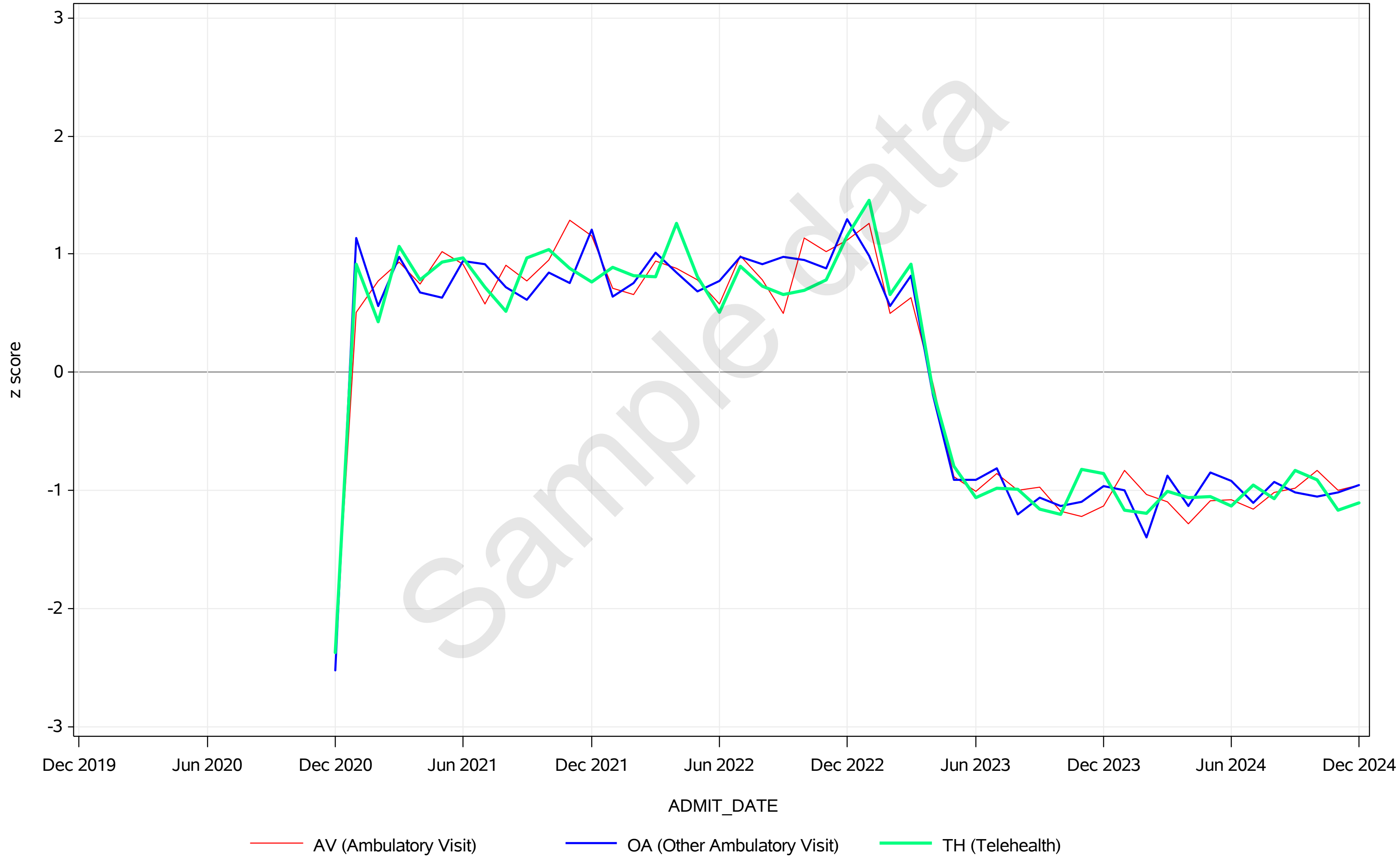

Chart IB. Trend in Encounters by Admit Date and Encounter Type, Past 5 Years (continued)

This 3 panel chart illustrates relative changes over time in the number of records per encounter type found in the ENCOUNTER table by admit date. The first panel includes AV, OA, and TH encounter types; the second panel includes ED, EI and IP encounter types, and the third panel includes IS, IC, and OS encounter types. For all charts, the X-axis is the 60 months prior to the maximum refresh date. Monthly record counts were standardized over the period shown to have a mean of 0 and a standard deviation of 1. The y-axis reflects the deviation in each month's count from the mean. A value above 0 indicates an above-average number of records; a value below 0 indicates a below-average number of records. Significant inflection points and other unexpected patterns should be investigated.

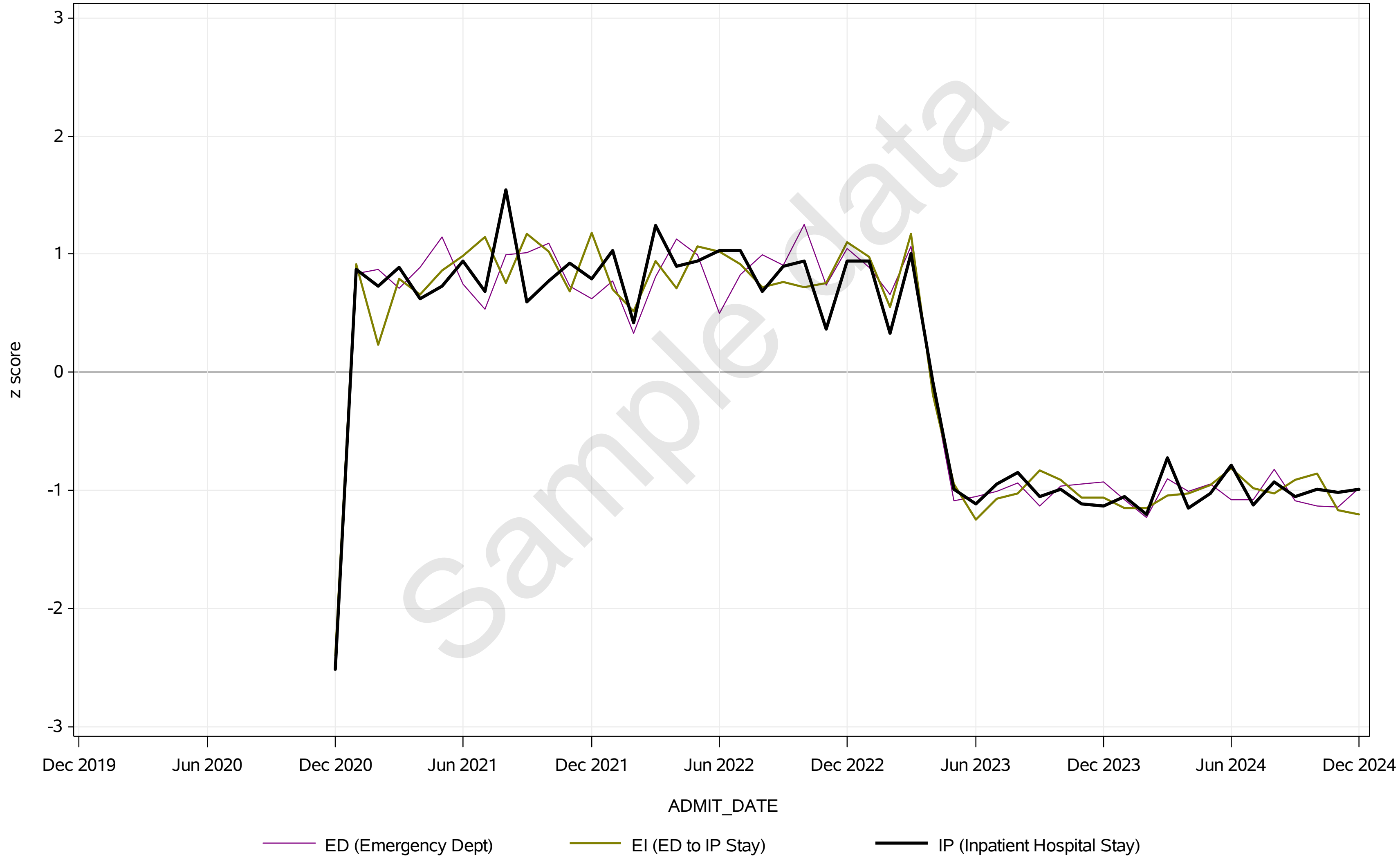

Chart IB. Trend in Encounters by Admit Date and Encounter Type, Past 5 Years (continued)

This 3 panel chart illustrates relative changes over time in the number of records per encounter type found in the ENCOUNTER table by admit date. The first panel includes AV, OA, and TH encounter types; the second panel includes ED, EI and IP encounter types, and the third panel includes IS, IC, and OS encounter types. For all charts, the X-axis is the 60 months prior to the maximum refresh date. Monthly record counts were standardized over the period shown to have a mean of 0 and a standard deviation of 1. The y-axis reflects the deviation in each month's count from the mean. A value above 0 indicates an above-average number of records; a value below 0 indicates a below-average number of records. Significant inflection points and other unexpected patterns should be investigated.

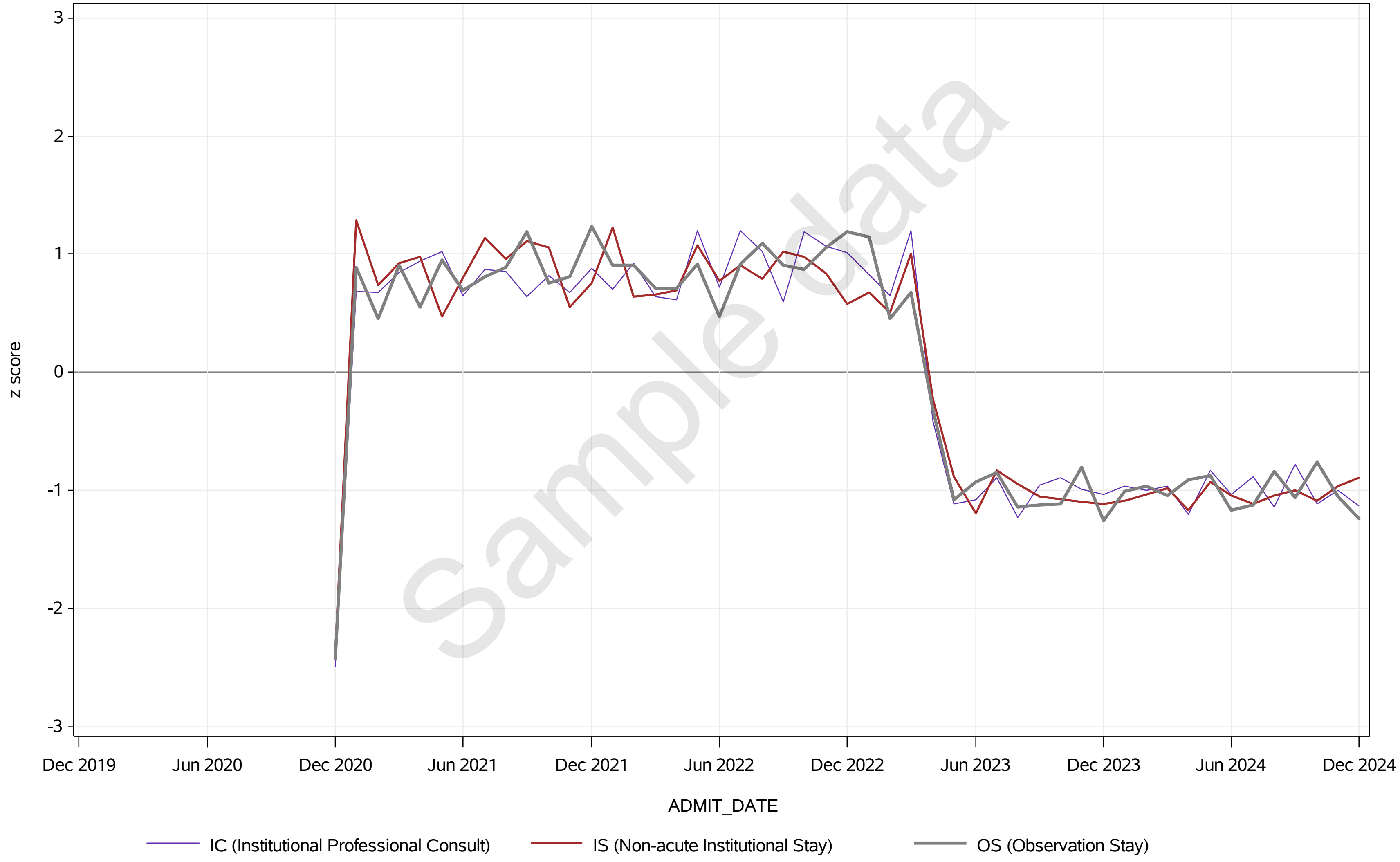

Chart IC. Trend in Institutional Encounters by Discharge Date and Encounter Type, Past 5 Years

This chart illustrates relative changes over time in the number of records for IP, IS, and EI encounter types found in the ENCOUNTER table by discharge date. For all charts, the X-axis is the 60 months prior to the maximum refresh date. Monthly record counts were standardized over the period shown to have a mean of 0 and a standard deviation of 1. The y-axis reflects the deviation in each month's count from the mean. A value above 0 indicates an above-average number of records; a value below 0 indicates a below-average number of records. Significant inflection points and other unexpected patterns should be investigated.

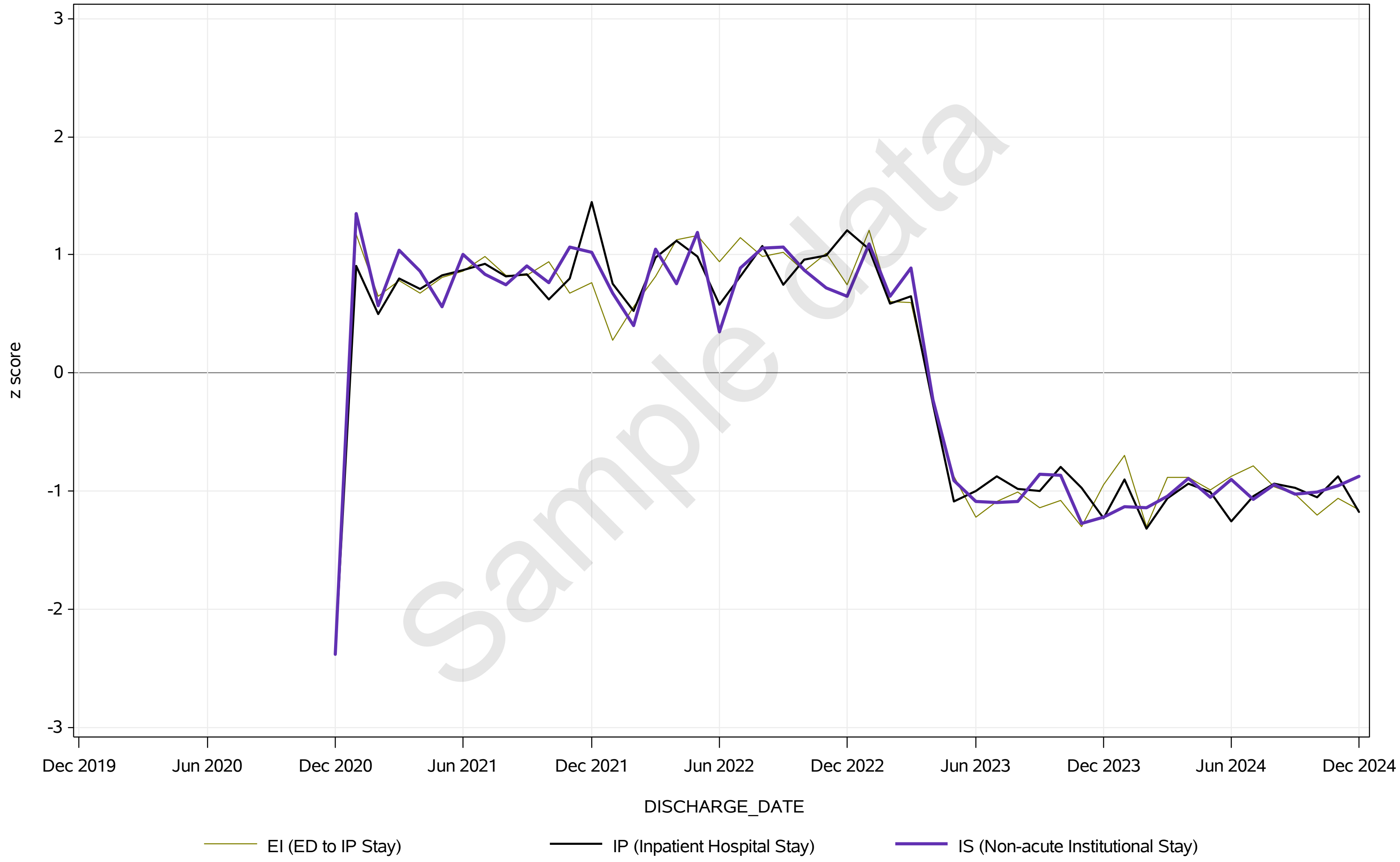

Chart ID. Trend in Lab Results By Result Date, Past 5 Years

This chart illustrates relative changes over time in the number of records found in the LAB_RESULT_CM table. For all charts, the X-axis is the 60 months prior to the maximum refresh date. Monthly record counts were standardized over the period shown to have a mean of 0 and a standard deviation of 1. The y-axis reflects the deviation in each month’s count from the mean. A value above 0 indicates an above-average number of records; a value below 0 indicates a below-average number of records. Significant inflection points and other unexpected patterns should be investigated.

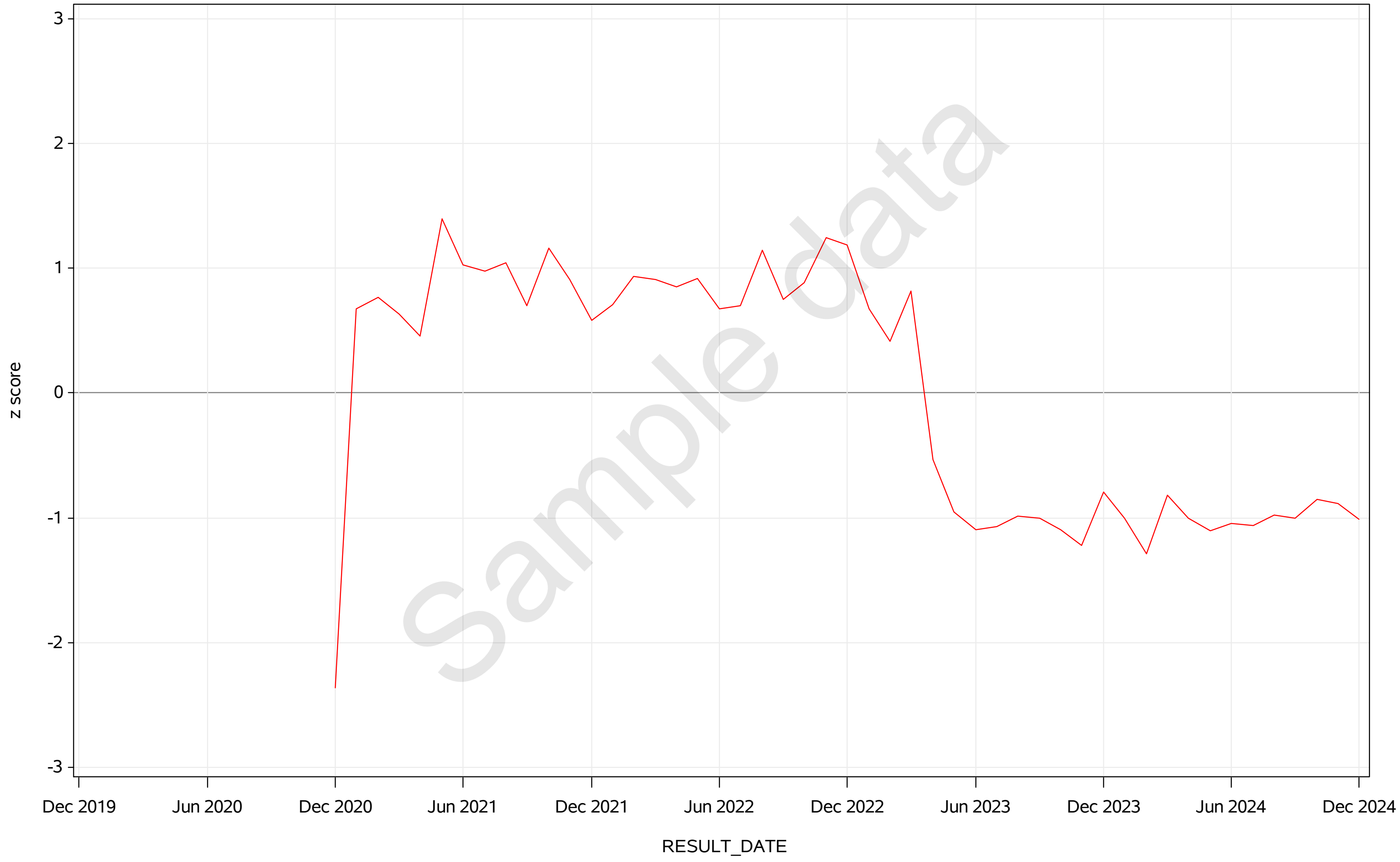

Chart IE. Trend in Prescribed Medications by Rx Order Date, Past 5 Years

This chart illustrates relative changes over time in the number of records found in the PRESCRIBING table. For all charts, the X-axis is the 60 months prior to the maximum refresh date. Monthly record counts were standardized over the period shown to have a mean of 0 and a standard deviation of 1. The y-axis reflects the deviation in each month’s count from the mean. A value above 0 indicates an above-average number of records; a value below 0 indicates a below-average number of records. Significant inflection points and other unexpected patterns should be investigated. Significant inflection points and other unexpected patterns should be investigated.

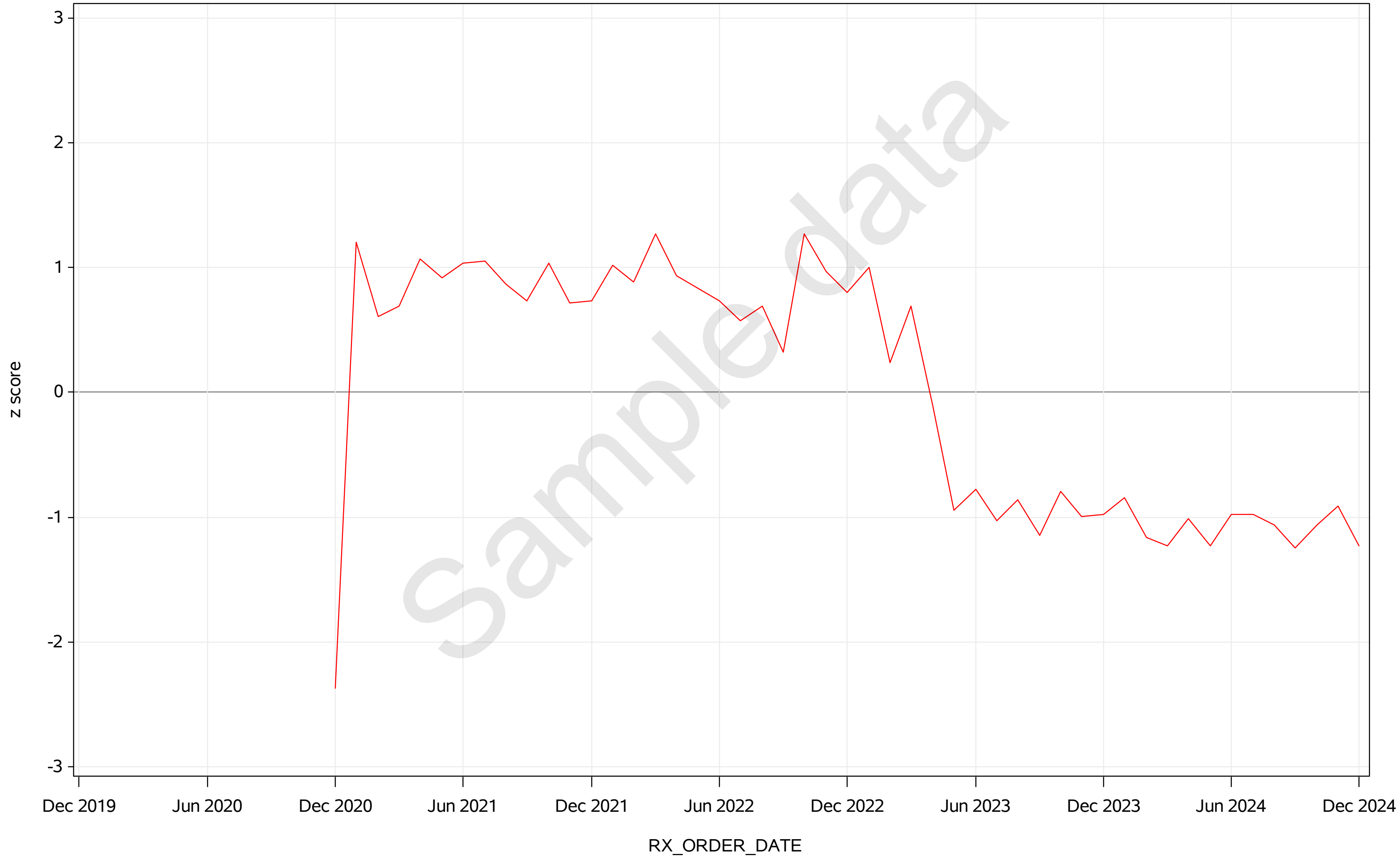

Chart IF. Trend in Dispensed Medications by Dispense Date, Past 5 Years

This chart illustrates relative changes over time in the number of records found in the DISPENSING table. For all charts, the X-axis is the 60 months prior to the maximum refresh date. Monthly record counts were standardized over the period shown to have a mean of 0 and a standard deviation of 1. The y-axis reflects the deviation in each month's count from the mean. A value above 0 indicates an above-average number of records; a value below 0 indicates a below-average number of records. Significant inflection points and other unexpected patterns should be investigated.

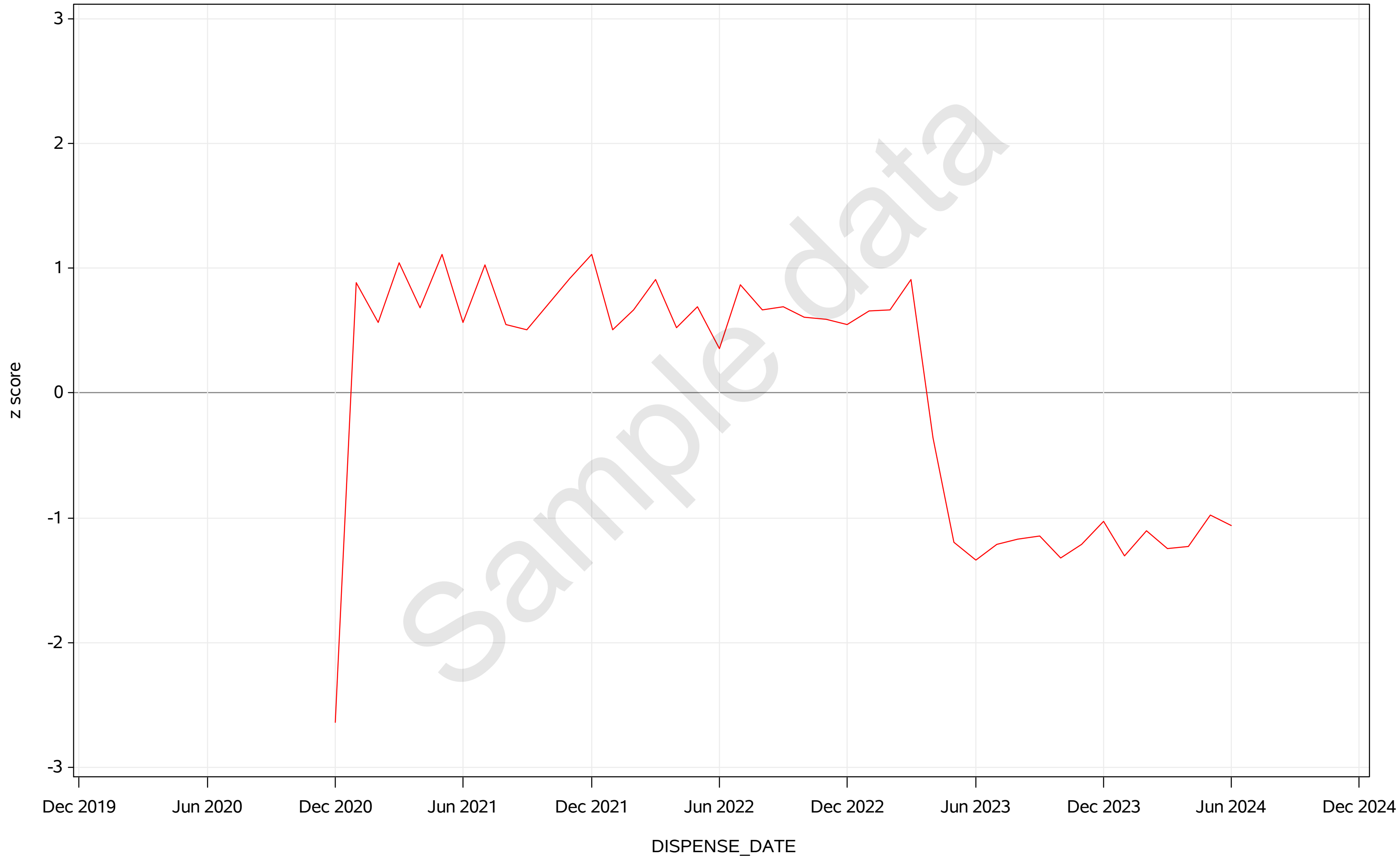

Chart IG. Trend in Administered Medications by Start Date, Past 5 Years

This chart illustrates relative changes over time in the number of records found in the MED_ADMIN table. For all charts, the X-axis is the 60 months prior to the maximum refresh date. Monthly record counts were standardized over the period shown to have a mean of 0 and a standard deviation of 1. The y-axis reflects the deviation in each month’s count from the mean. A value above 0 indicates an above-average number of records; a value below 0 indicates a below-average number of records. Significant inflection points and other unexpected patterns should be investigated.

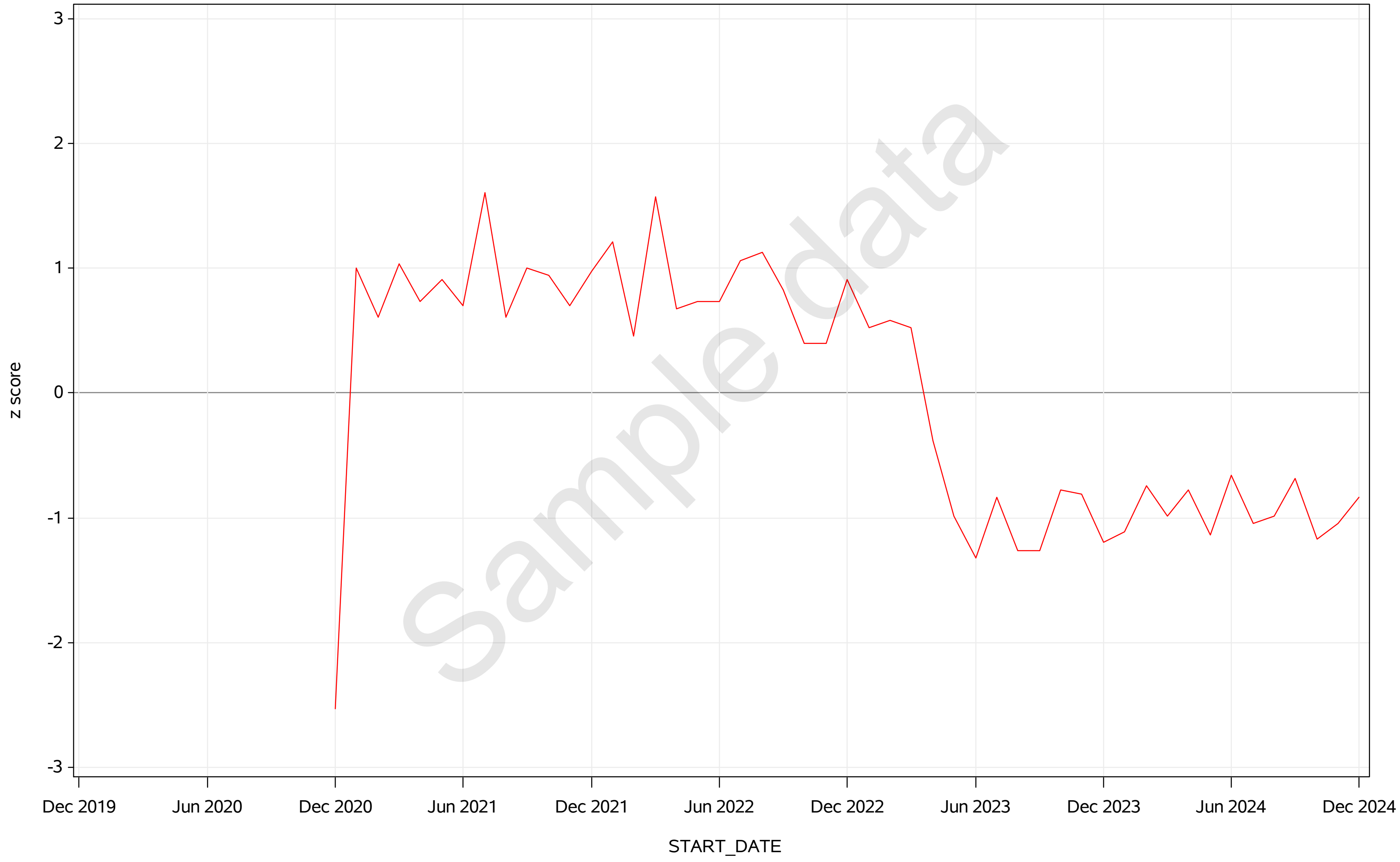

Chart II. Trend in Death Records by Death Date and Source, Past 5 Years

This chart illustrates relative changes over time in the number of records per death source found in the DEATH table. For all charts, the X-axis is the 60 months prior to the maximum refresh date. Monthly record counts were standardized over the period shown to have a mean of 0 and a standard deviation of 1. The y-axis reflects the deviation in each month's count from the mean. A value above 0 indicates an above-average number of records; a value below 0 indicates a below-average number of records. Significant inflection points and other unexpected patterns should be investigated.

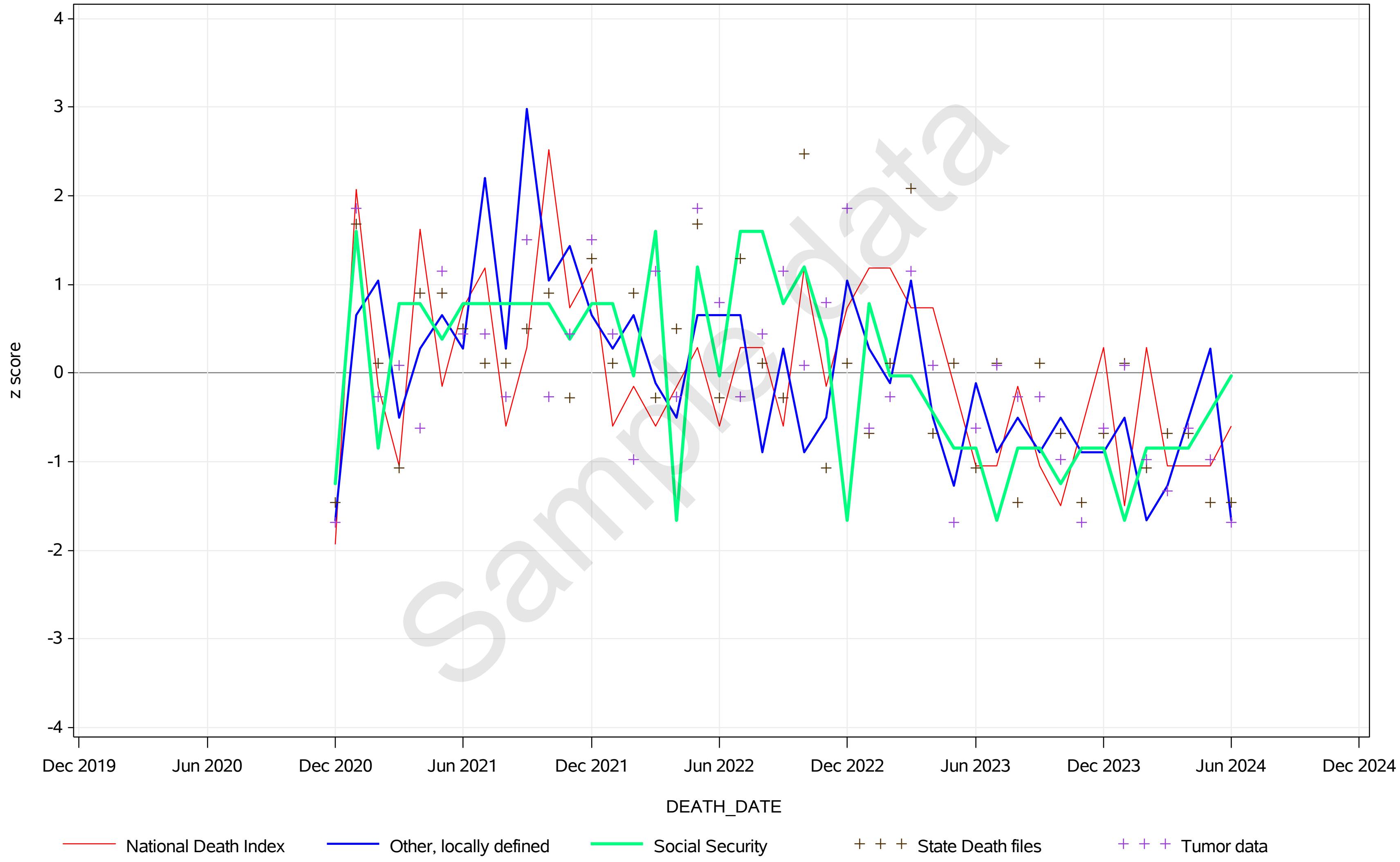

Chart IJ. Trend in Immunization Records by Vx Record Date, Past 5 Years

This chart illustrates relative changes over time in the number of records found in the IMMUNIZATION table. For all charts, the X-axis is the 60 months prior to the maximum refresh date. Monthly record counts were standardized over the period shown to have a mean of 0 and a standard deviation of 1. The y-axis reflects the deviation in each month’s count from the mean. A value above 0 indicates an above-average number of records; a value below 0 indicates a below-average number of records. Significant inflection points and other unexpected patterns should be investigated.

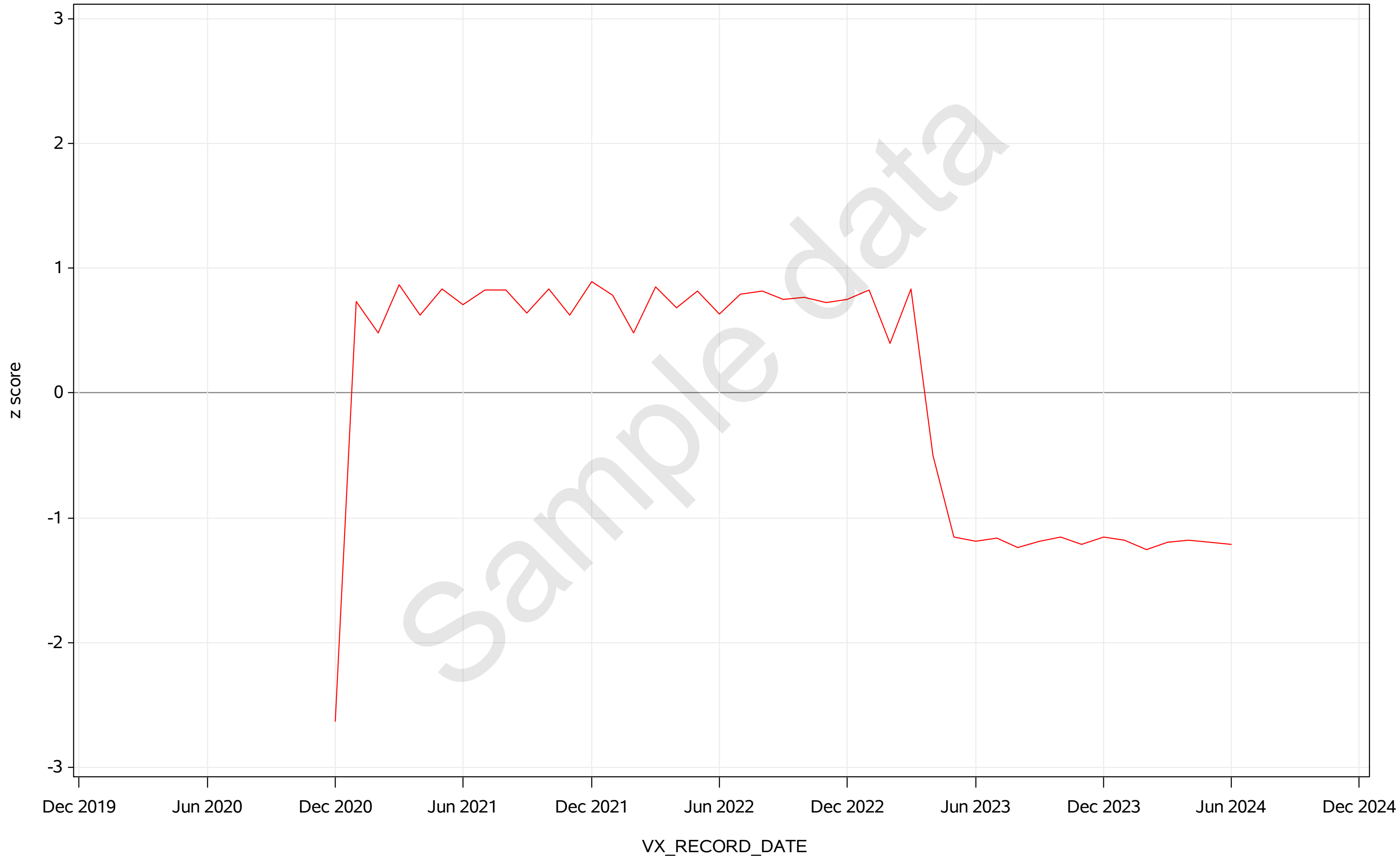

Chart IK. Trend in Clinical Observation Records by Start Date, Past 5 Years

This chart illustrates relative changes over time in the number of records found in the OBS_CLIN table. For all charts, the X-axis is the 60 months prior to the maximum refresh date. Monthly record counts were standardized over the period shown to have a mean of 0 and a standard deviation of 1. The y-axis reflects the deviation in each month's count from the mean. A value above 0 indicates an above-average number of records; a value below 0 indicates a below-average number of records. Significant inflection points and other unexpected patterns should be investigated.

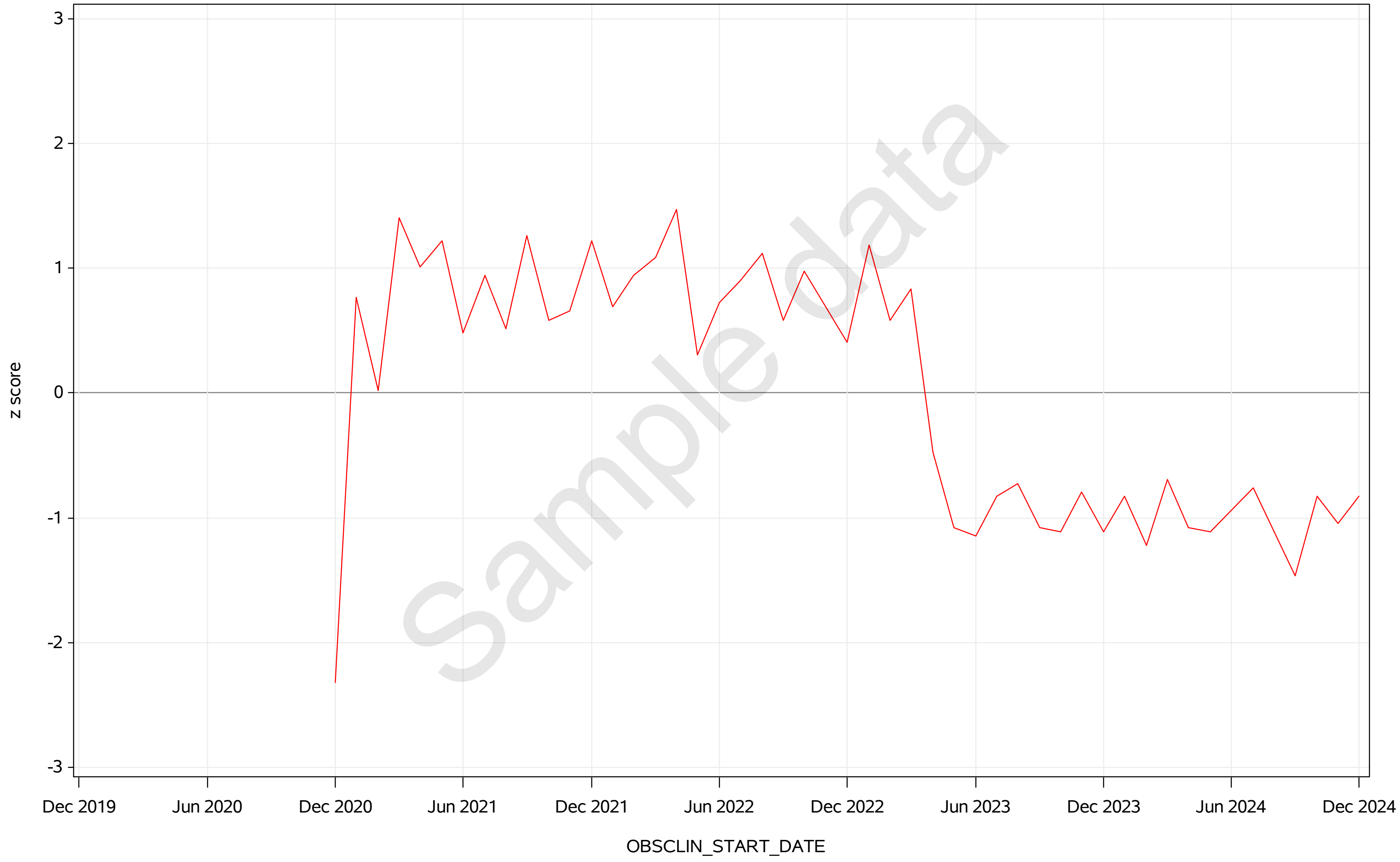

Table IIA. Primary Key Errors

This table shows the required primary key definitions and supports Data Check 1.05 (primary key definition errors). Data check exceptions are highlighted in red and must be corrected.

Table	CDM specifications for primary keys	Exception to specifications	Source table
DEMOGRAPHIC	PATID is unique	No	DEM_L3_N
DEATH	DEATHID (concatenation of PATID and DEATH_SOURCE) is unique	No	DEATH_L3_N
ENCOUNTER	ENCOUNTERID is unique	No	ENC_L3_N
DIAGNOSIS	DIAGNOSISID is unique	No	DIA_L3_N
PROCEDURES	PROCEDURESID is unique	No	PRO_L3_N
VITAL	VITALID is unique	No	VIT_L3_N
PRESCRIBING	PRESCRIBINGID is unique	No	PRES_L3_N
DISPENSING	DISPENSINGID is unique	No	DISP_L3_N
LAB_RESULT_CM	LAB_RESULT_CM_ID is unique	No	LAB_L3_N
HARVEST	NETWORKID+DATAMARTID is unique	No	XTBL_L3_METADATA
PROVIDER	PROVIDERID is unique	No	PROV_L3_N
MED_ADMIN	MEDADMINID is unique	No	MEDADM_L3_N
OBS_CLIN	OBSCLINID is unique	No	OBSCLIN_L3_N
HASH_TOKEN	HASHID (concatenation of PATID + TOKEN_ENCRYPTION_KEY) is unique	No	HASH_L3_N
IMMUNIZATION	IMMUNIZATIONID is unique	No	IMMUNE_L3_N

Sample data

The data checking logic is that the count of all records (ALL_N) and distinct records for the variable of interest (DISTINCT_N) must match.

Table IIB. Values Outside of CDM Specifications

This table lists all fields with pre-defined value sets and supports Data Check 1.06 (required fields contain values outside of data model specifications). Data check exceptions are highlighted in red and must be corrected.

Table	Field	Number of records with values outside of specifications	Source table
DEMOGRAPHIC	SEX	0	DEM_L3_SEXDIST
DEMOGRAPHIC	HISPANIC	0	DEM_L3_HISPDIST
DEMOGRAPHIC	RACE	0	DEM_L3_RACEDIST
DEMOGRAPHIC	PAT_PREF_LANGUAGE_SPOKEN	0	DEM_L3_PATPREFLANG
DEMOGRAPHIC	GENDER_IDENTITY	0	DEM_L3_GENDERDIST
DEMOGRAPHIC	SEXUAL_ORIENTATION	**37**	DEM_L3_ORIENTDIST
DEATH	DEATH_DATE_IMPUTE	0	DEATH_L3_IMPUTE
DEATH	DEATH_SOURCE	0	DEATH_L3_SOURCE
DEATH	DEATH_MATCH_CONFIDENCE	0	DEATH_L3_MATCH
ENCOUNTER	ENC_TYPE	0	ENC_L3_ENCTYPE
ENCOUNTER	DISCHARGE_DISPOSITION	0	ENC_L3_DISDISP
ENCOUNTER	DISCHARGE_STATUS	0	ENC_L3_DISSTAT
ENCOUNTER	DRG_TYPE	0	ENC_L3_DRG_TYPE
ENCOUNTER	ADMITTING_SOURCE	0	ENC_L3_ADMSRC
ENCOUNTER	PAYER_TYPE_PRIMARY	0	ENC_L3_PAYERTYPE1
ENCOUNTER	PAYER_TYPE_SECONDARY	0	ENC_L3_PAYERTYPE2
ENCOUNTER	FACILITY_TYPE	0	ENC_L3_FACILITYTYPE
ENCOUNTER	FACILITY_LOCATION	**107,044**	ENC_L3_N
DIAGNOSIS	ENC_TYPE	0	DIA_L3_ENCTYPE
DIAGNOSIS	DX_TYPE	0	DIA_L3_DXTYPE
DIAGNOSIS	DX_SOURCE	0	DIA_L3_DXSOURCE
DIAGNOSIS	PDX	0	DIA_L3_PDX
DIAGNOSIS	DX_POA	0	DIA_L3_DXPOA
DIAGNOSIS	DX_ORIGIN	0	DIA_L3_ORIGIN
PROCEDURES	ENC_TYPE	0	PRO_L3_ENCTYPE
PROCEDURES	PX_TYPE	0	PRO_L3_PXTYPE
PROCEDURES	PX_SOURCE	0	PRO_L3_PXSOURCE
PROCEDURES	PPX	0	PRO_L3_PPX

Sample data

NDC result is calculated as the difference between the total number of records with NDC codes minus the number of records with NDC codes in the HIPAA format (11-digit, no-dash).

Table IIB. Values Outside of CDM Specifications (continued - page 2 of 5)
This table lists all fields with pre-defined value sets and supports Data Check 1.06 (required fields contain values outside of data model specifications). Data check exceptions are highlighted in red and must be corrected.

Table	Field	Number of records with values outside of specifications	Source table
VITAL	VITAL_SOURCE	0	VIT_L3_VITAL_SOURCE
VITAL	BP_POSITION	0	VIT_L3_BP_POSITION_TYPE
VITAL	SMOKING	0	VIT_L3_SMOKING
VITAL	TOBACCO	0	VIT_L3_TOBACCO
VITAL	TOBACCO_TYPE	**3,765**	VIT_L3_TOBACCO_TYPE
PRESCRIBING	RX_BASIS	0	PRES_L3_BASIS
PRESCRIBING	RX_FREQUENCY	0	PRES_L3_FREQ
PRESCRIBING	RX_DOSE_FORM	0	PRES_L3_RXDOSEFORM
PRESCRIBING	RX_DOSE_ORDERED_UNIT	0	PRES_L3_RXDOSEODRUNIT
PRESCRIBING	RX_PRN_FLAG	0	PRES_L3_PRNFLAG
PRESCRIBING	RX_ROUTE	0	PRES_L3_ROUTE
PRESCRIBING	RX_SOURCE	0	PRES_L3_SOURCE
PRESCRIBING	RX_DISPENSE_AS_WRITTEN	0	PRES_L3_DISPASWRTN
DISPENSING	NDC	0	DISP_L3_N
DISPENSING	DISPENSE_DOSE_DISP_UNIT	0	DISP_L3_DOSEUNIT
DISPENSING	DISPENSE_ROUTE	0	DISP_L3_ROUTE
DISPENSING	DISPENSE_SOURCE	0	DISP_L3_SOURCE
LAB_RESULT_CM	LAB_LOINC_SOURCE	0	LAB_L3_LSOURCE
LAB_RESULT_CM	LAB_RESULT_SOURCE	**10,508**	LAB_L3_RSOURCE
LAB_RESULT_CM	SPECIMEN_SOURCE	0	LAB_L3_SOURCE
LAB_RESULT_CM	PRIORITY	0	LAB_L3_PRIORITY
LAB_RESULT_CM	RESULT_LOC	0	LAB_L3_LOC
LAB_RESULT_CM	LAB_PX_TYPE	0	LAB_L3_PX_TYPE
LAB_RESULT_CM	RESULT_QUAL	0	LAB_L3_QUAL
LAB_RESULT_CM	RESULT_MODIFIER	0	LAB_L3_MOD
LAB_RESULT_CM	NORM_MODIFIER_LOW	0	LAB_L3_LOW
LAB_RESULT_CM	NORM_MODIFIER_HIGH	0	LAB_L3_HIGH
LAB_RESULT_CM	ABN_IND	0	LAB_L3_ABN
LAB_RESULT_CM	RESULT_UNIT	0	LAB_L3_UNIT

Sample data

NDC result is calculated as the difference between the total number of records with NDC codes minus the number of records with NDC codes in the HIPAA format (11-digit, no-dash).

Table IIB. Values Outside of CDM Specifications (continued - page 3 of 5)
This table lists all fields with pre-defined value sets and supports Data Check 1.06 (required fields contain values outside of data model specifications). Data check exceptions are highlighted in red and must be corrected.

Table	Field	Number of records with values outside of specifications	Source table
HARVEST	DATAMART_PLATFORM	0	XTBL_L3_METADATA
HARVEST	DATAMART_CLAIMS	0	XTBL_L3_METADATA
HARVEST	DATAMART_EHR	0	XTBL_L3_METADATA
HARVEST	BIRTH_DATE_MGMT	0	XTBL_L3_METADATA
HARVEST	ENR_START_DATE_MGMT	0	XTBL_L3_METADATA
HARVEST	ENR_END_DATE_MGMT	0	XTBL_L3_METADATA
HARVEST	ADMIT_DATE_MGMT	0	XTBL_L3_METADATA
HARVEST	DISCHARGE_DATE_MGMT	0	XTBL_L3_METADATA
HARVEST	PX_DATE_MGMT	0	XTBL_L3_METADATA
HARVEST	DX_DATE_MGMT	0	XTBL_L3_METADATA
HARVEST	RX_ORDER_DATE_MGMT	0	XTBL_L3_METADATA
HARVEST	RX_START_DATE_MGMT	0	XTBL_L3_METADATA
HARVEST	RX_END_DATE_MGMT	0	XTBL_L3_METADATA
HARVEST	DISPENSE_DATE_MGMT	0	XTBL_L3_METADATA
HARVEST	LAB_ORDER_DATE_MGMT	0	XTBL_L3_METADATA
HARVEST	SPECIMEN_DATE_MGMT	0	XTBL_L3_METADATA
HARVEST	RESULT_DATE_MGMT	0	XTBL_L3_METADATA
HARVEST	MEASURE_DATE_MGMT	0	XTBL_L3_METADATA
HARVEST	ONSET_DATE_MGMT	0	XTBL_L3_METADATA
HARVEST	REPORT_DATE_MGMT	0	XTBL_L3_METADATA
HARVEST	RESOLVE_DATE_MGMT	0	XTBL_L3_METADATA
HARVEST	PRO_DATE_MGMT	0	XTBL_L3_METADATA
HARVEST	DEATH_DATE_MGMT	0	XTBL_L3_METADATA
HARVEST	MEDADMIN_START_DATE_MGMT	0	XTBL_L3_METADATA
HARVEST	MEDADMIN_STOP_DATE_MGMT	0	XTBL_L3_METADATA
HARVEST	OBSCLIN_START_DATE_MGMT	0	XTBL_L3_METADATA
HARVEST	OBSGEN_START_DATE_MGMT	0	XTBL_L3_METADATA

Sample data

NDC result is calculated as the difference between the total number of records with NDC codes minus the number of records with NDC codes in the HIPAA format (11-digit, no-dash).

Table IIB. Values Outside of CDM Specifications (continued - page 4 of 5)

This table lists all fields with pre-defined value sets and supports Data Check 1.06 (required fields contain values outside of data model specifications). Data check exceptions are highlighted in red and must be corrected.

Table	Field	Number of records with values outside of specifications	Source table
HARVEST	ADDRESS_PERIOD_START_MGMT	0	XTBL_L3_METADATA
HARVEST	ADDRESS_PERIOD_END_MGMT	0	XTBL_L3_METADATA
HARVEST	VX_RECORD_DATE_MGMT	0	XTBL_L3_METADATA
HARVEST	VX_ADMIN_DATE_MGMT	0	XTBL_L3_METADATA
HARVEST	VX_EXP_DATE_MGMT	0	XTBL_L3_METADATA
HARVEST	OBSCLIN_STOP_DATE_MGMT	0	XTBL_L3_METADATA
HARVEST	OBSGEN_STOP_DATE_MGMT	0	XTBL_L3_METADATA
HARVEST	CDM_VERSION	0	XTBL_L3_METADATA
PROVIDER	PROVIDER_SPECIALTY_PRIMARY	0	PROV_L3_SPECIALTY
PROVIDER	PROVIDER_SEX	0	PROV_L3_SEX
PROVIDER	PROVIDER_NPI_FLAG	0	PROV_L3_NPIFLAG
MED_ADMIN	MEDADMIN_DOSE_ADMIN_UNIT	0	MEDADM_L3_DOSEADMUNIT
MED_ADMIN	MEDADMIN_ROUTE	0	MEDADM_L3_ROUTE
MED_ADMIN	MEDADMIN_SOURCE	0	MEDADM_L3_SOURCE
MED_ADMIN	MEDADMIN_TYPE	0	MEDADM_L3_TYPE

Sample data

NDC result is calculated as the difference between the total number of records with NDC codes minus the number of records with NDC codes in the HIPAA format (11-digit, no-dash).

Table IIB. Values Outside of CDM Specifications (continued - page 5 of 5)
This table lists all fields with pre-defined value sets and supports Data Check 1.06 (required fields contain values outside of data model specifications). Data check exceptions are highlighted in red and must be corrected.

Table	Field	Number of records with values outside of specifications	Source table
OBS_CLIN	OBSCLIN_RESULT_MODIFIER	0	OBSCLIN_L3_MOD
OBS_CLIN	OBSCLIN_RESULT_QUAL	0	OBSCLIN_L3_QUAL
OBS_CLIN	OBSCLIN_RESULT_UNIT	0	OBSCLIN_L3_RUNIT
OBS_CLIN	OBSCLIN_TYPE	0	OBSCLIN_L3_TYPE
OBS_CLIN	OBSCLIN_SOURCE	0	OBSCLIN_L3_SOURCE
OBS_CLIN	OBSCLIN_ABN_IND	0	OBSCLIN_L3_ABN
IMMUNIZATION	VX_CODE_TYPE	0	IMMUNE_L3_CODE_CODETYPE
IMMUNIZATION	VX_STATUS	0	IMMUNE_L3_STATUS
IMMUNIZATION	VX_STATUS_REASON	0	IMMUNE_L3_STATUSREASON
IMMUNIZATION	VX_SOURCE	0	IMMUNE_L3_SOURCE
IMMUNIZATION	VX_DOSE_UNIT	0	IMMUNE_L3_DOSEUNIT
IMMUNIZATION	VX_ROUTE	0	IMMUNE_L3_ROUTE
IMMUNIZATION	VX_BODY_SITE	0	IMMUNE_L3_BODYSITE
IMMUNIZATION	VX_MANUFACTURER	0	IMMUNE_L3_MANUFACTURER

Sample data

NDC result is calculated as the difference between the total number of records with NDC codes minus the number of records with NDC codes in the HIPAA format (11-digit, no-dash).

Table IIC. Non-Permissible Missing Values

This table lists all fields which are required to be populated, as defined by the table constraints in the CDM, and supports Data Check 1.07 (required fields have non-permissible missing values). Data check exceptions are highlighted in red and must be corrected.

Table	Field	Number of records with missing values	Source table
All fields conform to specifications			

Sample data

Table IID. Diagnostic Errors

This table illustrates exceptions to Data Checks 1.01 (required tables are not present), 1.02 (required tables are not populated), 1.03 (required fields are not present), 1.04 (required fields do not conform to data model specifications for data type, length, or name), 1.15 (fields with undefined lengths that are present in more than one table do not have harmonized field lengths^) and Data Check 1.17 (Zip codes in the ENCOUNTER or LDS_ADDRESS_HISTORY table do not conform to expected values). Data check exceptions are highlighted in red and must be corrected.

Data Check	Data Check Description	Exception	Table(s)	Field(s)	Source table(s)
1.01	Required tables are not present	Required table is not present. All tables must be present in an instantiation of the CDM	None	n/a	DATAMART_ALL
1.02	Expected tables are not populated	Table expected to be populated (DEMOGRAPHIC, ENROLLMENT, ENCOUNTER, DIAGNOSIS, PROCEDURES, and HARVEST) is not populated	**ENROLLMENT**	n/a	DATAMART_ALL
1.03	Required fields are not present	Required numeric field is not present	None	None	DATAMART_ALL
1.03	Required fields are not present	Required character field is not present	**ENCOUNTER**	**RAW_FACILITY_TYPE**	DATAMART_ALL
1.04	Required fields do not conform to data model specifications for data type, length, or name	Required character field is numeric	**LAB_RESULT_CM**	**LAB_RESULT_SOURCE**	DATAMART_ALL
1.04	Required fields do not conform to data model specifications for data type, length, or name	Required numeric field is character	None	None	DATAMART_ALL
1.04	Required fields do not conform to data model specifications for data type, length, or name	Required field is present but of unexpected length	**VITAL**	**TOBACCO_TYPE**	DATAMART_ALL
1.15	Fields with undefined lengths that are present in more than one table do not have harmonized field lengths	Field lengths are not harmonized for one or more of the following fields: PATID, ENCOUNTERID, PRESCRIBINGID, PROCEDURESID, PROVIDERID, MEDADMIN_PROVIDERID, OBSGEN_PROVIDERID, OBSCLIN_PROVIDERID, RX_PROVIDERID, and VX_PROVIDERID.		**PATID**	DATAMART_ALL

Sample data

Diagnostic checks are performed on all CDM tables and fields. Diagnostic checks ensure conformance to CDM v6.1 table structure, variable names, SAS variable lengths, and SAS data types. ^ To identify the differing field lengths, either open the datamart_all.sas7bdat dataset (in dmlocal) and review the field length attribute or run the CDM Diagnostic Query, remembering that the provider identifier field varies by table (PROVIDERID, MEDADMIN_PROVIDERID, OBSGEN_PROVIDERID, OBSCLIN_PROVIDERID, RX_PROVIDERID, or VX_PROVIDERID).

Table IID. Diagnostic Errors (continued)

This table illustrates exceptions to Data Checks 1.01 (required tables are not present), 1.02 (required tables are not populated), 1.03 (required fields are not present), 1.04 (required fields do not conform to data model specifications for data type, length, or name), 1.15 (fields with undefined lengths that are present in more than one table do not have harmonized field lengths^) and Data Check 1.17 (Zip codes in the ENCOUNTER or LDS_ADDRESS_HISTORY table do not conform to expected values). Data check exceptions are highlighted in red and must be corrected.

Data Check	Data Check Description	Exception	Table(s)	Field(s)	Source table(s)
1.17	Zip codes in the ENCOUNTER or LDS_ADDRESS_HISTORY table do not conform to expected values	LDS_ADDRESS_HISTORY.ADDRESS_ZIP5, LDS_ADDRESS_HISTORY.ADDRESS_ZIP9 or ENCOUNTER.FACILITY_LOCATION contains alphabetical characters or does not have the expected number of digits.	**ENCOUNTER**	**FACLITY_ LOCATION**	ENC_L3_N; LDSADRS_L3_N

Sample data

Diagnostic checks are performed on all CDM tables and fields. Diagnostic checks ensure conformance to CDM v6.1 table structure, variable names, SAS variable lengths, and SAS data types. ^ To identify the differing field lengths, either open the datamart_all.sas7bdat dataset (in dmlocal) and review the field length attribute or run the CDM Diagnostic Query, remembering that the provider identifier field varies by table (PROVIDERID, MEDADMIN_PROVIDERID, OBSGEN_PROVIDERID, OBSCLIN_PROVIDERID, RX_PROVIDERID, or VX_PROVIDERID).

Table IIE. Orphan Records, Replication Errors, Encounter Duplication and Hash Token Duplication

This table illustrates exceptions to Data Checks 1.08 (tables contain orphan PATIDs), 1.09 (tables contain orphan ENCOUNTERIDs for more than 5% of records), 1.10 (replication errors between the ENCOUNTER, PROCEDURES and DIAGNOSIS tables), 1.11 (more than 5% of encounters are assigned to more than one patient), 1.12 (tables contain orphan PROVIDERIDs), 1.14 (patients are missing from HASH_TOKEN), and 1.19 (more than 10% of hash tokens are assigned to multiple patients). Orphan PATIDs are not present in the DEMOGRAPHIC table. Orphan ENCOUNTERIDs are not present in the ENCOUNTER table. Orphan PROVIDERIDs are not present in the PROVIDER table. Replication errors are ENCOUNTERIDs in the DIAGNOSIS or PROCEDURES table where the encounter type or admit date does not match the corresponding value in the ENCOUNTER table. Data check exceptions to 1.14 and 1.19 are highlighted in blue and must be explained in the ETL ADD; all other data check exceptions are highlighted in red and must be corrected.

Data Check	Data Check Description	Exception	Table(s)	Field(s)	Count	%	Source table(s)
1.08	Orphan PATIDs	Orphan PATID(S) in the CONDITION, DIAGNOSIS, DEATH, DEATH_CAUSE, DISPENSING, ENCOUNTER, ENROLLMENT, HASH_TOKEN, IMMUNIZATION, LAB_RESULT_CM, LDS_ADDRESS_HISTORY, MED_ADMIN, OBS_CLIN, OBS_GEN, PCORNET_TRIAL, PRESCRIBING, PROCEDURES, PRO_CM, or VITAL table	ENCOUNTER	PATID	**1,058**	26.2	XTBL_L3_MISMATCH;
			DIAGNOSIS	PATID	**815**	26.0	ENR_L3_N; ENC_L3_N;
			PROCEDURES	PATID	**1,060**	26.5	DIA_L3_N; PRO_L3_N;
			VITAL	PATID	**604**	26.3	VIT_L3_N; LAB_L3_N;
			LAB_RESULT_CM	PATID	**1,157**	26.7	PRES_L3_N; DISP_L3_N;
			PRESCRIBING	PATID	**916**	27.2	DEATH_L3_N;
			DISPENSING	PATID	**1,227**	26.9	DEATHC_L3_N;
			DEATH	PATID	**306**	25.2	COND_L3_N;
			MED_ADMIN	PATID	**1,087**	26.7	PROCM_L3_N;
			OBS_CLIN	PATID	**858**	26.9	TRIAL_L3_N;
			HASH_TOKEN	PATID	**1,258**	26.7	OBSCLIN_L3_N;
			IMMUNIZATION	PATID	**1,045**	26.3	OBSGEN_L3_N;
							MEDADM_L3_N;
							HASH_L3_N;
							IMMUNE_L3_N;
							LDSADRS_L3_N

Sample data

For Data Checks 1.08, 1.09, 1.11 and 1.12, the count is the number of distinct IDs with an exception. For Data Check 1.10, the count is the number of records with replication errors. For Data Check 1.19, the count is the number of valid records (i.e. where the token is not an error code) where the tokens are not distinct (calculated as VALID_N minus VALID_DISTINCT_N).

Percent is calculated as the number of distinct IDs with exceptions (shown) divided by the total distinct IDs in the table (not shown). For example, if the DIAGNOSIS table has 2 orphan PATIDs (source table: XTBL_L3_MISMATCH) and 100 distinct PATIDs (source table: DEM_L3_N), the percentage of distinct IDs is 2.0%. For Data Check 1.19, the percent is calculated as 100%-valid_distinct_n_pct.

Source tables include all potential source tables for the data check calculations.

Table IIE. Orphan Records, Replication Errors, Encounter Duplication and Hash Token Duplication (continued - page 2 of 4)

This table illustrates exceptions to Data Checks 1.08 (tables contain orphan PATIDs), 1.09 (tables contain orphan ENCOUNTERIDs for more than 5% of records), 1.10 (replication errors between the ENCOUNTER, PROCEDURES and DIAGNOSIS tables), 1.11 (more than 5% of encounters are assigned to more than one patient), 1.12 (tables contain orphan PROVIDERIDs), 1.14 (patients are missing from HASH_TOKEN), and 1.19 (more than 10% of hash tokens are assigned to multiple patients). Orphan PATIDs are not present in the DEMOGRAPHIC table. Orphan ENCOUNTERIDs are not present in the ENCOUNTER table. Orphan PROVIDERIDs are not present in the PROVIDER table. Replication errors are ENCOUNTERIDs in the DIAGNOSIS or PROCEDURES table where the encounter type or admit date does not match the corresponding value in the ENCOUNTER table. Data check exceptions to 1.14 and 1.19 are highlighted in blue and must be explained in the ETL ADD; all other data check exceptions are highlighted in red and must be corrected.

Data Check	Data Check Description	Exception	Table(s)	Field(s)	Count	%	Source table(s)
1.09	Orphan ENCOUNTERIDs	Orphan ENCOUNTERID(S) for more than 5% of the records in the CONDITION, DIAGNOSIS, IMMUNIZATION, LAB_RESULT_CM, MED_ADMIN, OBS_CLIN, OBS_GEN, PRESCRIBING, PROCEDURES, PRO_CM, or VITAL table	DIAGNOSIS	ENCOUNTERID	30,234	**94.4**	XTBL_L3_MISMATCH; DIA_L3_N; PRO_L3_N; VIT_L3_N; PRES_L3_N; LAB_L3_N; PROCM_L3_N; COND_L3_N; OBSCLIN_L3_N; OBSGEN_L3_N; MEDADM_L3_N; IMMUNE_L3_N
			PROCEDURES	ENCOUNTERID	177,076	**95.1**	
			VITAL	ENCOUNTERID	2	**100.0**	
			PRESCRIBING	ENCOUNTERID	412	**10.9**	
			MED_ADMIN	ENCOUNTERID	422	**12.5**	
			IMMUNIZATION	ENCOUNTERID	150,673	**95.3**	
1.10	Replication errors	Replication error(s) in ENC_TYPE or ADMIT_DATE in the DIAGNOSIS or PROCEDURES table	DIAGNOSIS	ENC_TYPE	**1,628**	n/a	XTBL_L3_MISMATCH
			DIAGNOSIS	ADMIT_DATE	**1,796**	n/a	
			PROCEDURES	ENC_TYPE	**8,228**	n/a	
			PROCEDURES	ADMIT_DATE	**9,024**	n/a	

Sample data

For Data Checks 1.08, 1.09, 1.11 and 1.12, the count is the number of distinct IDs with an exception. For Data Check 1.10, the count is the number of records with replication errors. For Data Check 1.19, the count is the number of valid records (i.e. where the token is not an error code) where the tokens are not distinct (calculated as VALID_N minus VALID_DISTINCT_N).

Percent is calculated as the number of distinct IDs with exceptions (shown) divided by the total distinct IDs in the table (not shown). For example, if the DIAGNOSIS table has 2 orphan PATIDs (source table: XTBL_L3_MISMATCH) and 100 distinct PATIDs (source table: DEM_L3_N), the percentage of distinct IDs is 2.0%. For Data Check 1.19, the percent is calculated as 100%-valid_distinct_n_pct.

Source tables include all potential source tables for the data check calculations.

Table IIE. Orphan Records, Replication Errors, Encounter Duplication and Hash Token Duplication (continued - page 3 of 4)
This table illustrates exceptions to Data Checks 1.08 (tables contain orphan PATIDs), 1.09 (tables contain orphan ENCOUNTERIDs for more than 5% of records), 1.10 (replication errors between the ENCOUNTER, PROCEDURES and DIAGNOSIS tables), 1.11 (more than 5% of encounters are assigned to more than one patient), 1.12 (tables contain orphan PROVIDERIDs), 1.14 (patients are missing from HASH_TOKEN), and 1.19 (more than 10% of hash tokens are assigned to multiple patients). Orphan PATIDs are not present in the DEMOGRAPHIC table. Orphan ENCOUNTERIDs are not present in the ENCOUNTER table. Orphan PROVIDERIDs are not present in the PROVIDER table. Replication errors are ENCOUNTERIDs in the DIAGNOSIS or PROCEDURES table where the encounter type or admit date does not match the corresponding value in the ENCOUNTER table. Data check exceptions to 1.14 and 1.19 are highlighted in blue and must be explained in the ETL ADD; all other data check exceptions are highlighted in red and must be corrected.

Data Check	Data Check Description	Exception	Table(s)	Field(s)	Count	%	Source table(s)
1.11	More than 5% of encounters	An ENCOUNTERID in the	PRESCRIBING	ENCOUNTERID	1	0.0	XTBL_L3_NON_UNIQUE;
	are assigned to more than	CONDITION, DIAGNOSIS,	VITAL	ENCOUNTERID	1	**50.0**	COND_L3_N; DIA_L3_N;
	one patient	ENCOUNTER, IMMUNIZATION,	MED_ADMIN	ENCOUNTERID	1	0.0	ENC_L3_N: LAB_L3_N;
		LAB_RESULT_CM, MED_ADMIN,	IMMUNIZATION	ENCOUNTERID	1	0.0	PRES_L3_N; PRO_L3_N;
		OBS_CLIN, OBS_GEN, PRESCRIBING,					VIT_L3_N;
		PROCEDURES, PRO_CM, or VITAL					PROCM_L3_N;
		table is associated with more					OBSCLIN_L3_N;
		than 1 PATID in the same table					OBSGEN_L3_N;
							MEDADM_L3_N;
							IMMUNE_L3_N
1.12	Orphan PROVIDERIDs	Orphan PROVIDERID(S) in the	ENCOUNTER	PROVIDERID	**2,995**	22.6	XTBL_L3_MISMATCH;
		ENCOUNTER, DIAGNOSIS,	DIAGNOSIS	PROVIDERID	**1,516**	22.6	DIA_L3_N; ENC_L3_N;
		IMMUNIZATION, MED_ADMIN,	PROCEDURES	PROVIDERID	**2,870**	22.9	MEDADM_L3_N;
		OBS_CLIN, OBS_GEN, PRESCRIBING,	PRESCRIBING	PROVIDERID	**935**	26.7	OBSCLIN_L3_N;
		or PROCEDURES table.	MED_ADMIN	PROVIDERID	**525**	22.8	OBSGEN_L3_N;
			OBS_CLIN	PROVIDERID	**421**	21.5	PRES_L3_N; PRO_L3_N;
			IMMUNIZATION	PROVIDERID	**2,788**	22.9	IMMUNE_L3_N
1.14	A PATID in the	A PATID in the DEMOGRAPHIC	DEMOGRAPHIC	PATID	**182**	5.0	XTBL_L3_MISMATCH
	DEMOGRAPHIC table is not	table is not included in the					
	included in the HASH_TOKEN	HASH_TOKEN table					

Sample data

For Data Checks 1.08, 1.09, 1.11 and 1.12, the count is the number of distinct IDs with an exception. For Data Check 1.10, the count is the number of records with replication errors. For Data Check 1.19, the count is the number of valid records (i.e. where the token is not an error code) where the tokens are not distinct (calculated as VALID_N minus VALID_DISTINCT_N).
Percent is calculated as the number of distinct IDs with exceptions (shown) divided by the total distinct IDs in the table (not shown). For example, if the DIAGNOSIS table has 2 orphan PATIDs (source table: XTBL_L3_MISMATCH) and 100 distinct PATIDs (source table: DEM_L3_N), the percentage of distinct IDs is 2.0%. For Data Check 1.19, the percent is calculated as 100%-valid_distinct_n_pct.
Source tables include all potential source tables for the data check calculations.

Table IIE. Orphan Records, Replication Errors, Encounter Duplication and Hash Token Duplication (continued - page 4 of 4)

This table illustrates exceptions to Data Checks 1.08 (tables contain orphan PATIDs), 1.09 (tables contain orphan ENCOUNTERIDs for more than 5% of records), 1.10 (replication errors between the ENCOUNTER, PROCEDURES and DIAGNOSIS tables), 1.11 (more than 5% of encounters are assigned to more than one patient), 1.12 (tables contain orphan PROVIDERIDs), 1.14 (patients are missing from HASH_TOKEN), and 1.19 (more than 10% of hash tokens are assigned to multiple patients). Orphan PATIDs are not present in the DEMOGRAPHIC table. Orphan ENCOUNTERIDs are not present in the ENCOUNTER table. Orphan PROVIDERIDs are not present in the PROVIDER table. Replication errors are ENCOUNTERIDs in the DIAGNOSIS or PROCEDURES table where the encounter type or admit date does not match the corresponding value in the ENCOUNTER table. Data check exceptions to 1.14 and 1.19 are highlighted in blue and must be explained in the ETL ADD; all other data check exceptions are highlighted in red and must be corrected.

Data Check	Data Check Description	Exception	Table(s)	Field(s)	Count	%	Source table(s)
1.19	More than 10% of hash tokens are assigned to multiple patients	More than 10% of the values in any hash token field are assigned to multiple patients	HASH_TOKEN	TOKEN_01	4,044	**98.8**	HASH_L3_N
			HASH_TOKEN	TOKEN_02	0		
			HASH_TOKEN	TOKEN_03	0		
			HASH_TOKEN	TOKEN_04	0		
			HASH_TOKEN	TOKEN_05	0		
			HASH_TOKEN	TOKEN_06	0		
			HASH_TOKEN	TOKEN_07	0		
			HASH_TOKEN	TOKEN_08	0		
			HASH_TOKEN	TOKEN_09	0		
			HASH_TOKEN	TOKEN_101	0		
			HASH_TOKEN	TOKEN_102	0		
			HASH_TOKEN	TOKEN_103	0		
			HASH_TOKEN	TOKEN_104	0		
			HASH_TOKEN	TOKEN_105	0		
			HASH_TOKEN	TOKEN_106	0		
			HASH_TOKEN	TOKEN_107	0		
			HASH_TOKEN	TOKEN_108	0		
			HASH_TOKEN	TOKEN_109	0		
			HASH_TOKEN	TOKEN_110	0		
			HASH_TOKEN	TOKEN_111	0		
			HASH_TOKEN	TOKEN_12	0		
			HASH_TOKEN	TOKEN_14	0		

For Data Checks 1.08, 1.09, 1.11 and 1.12, the count is the number of distinct IDs with an exception. For Data Check 1.10, the count is the number of records with replication errors. For Data Check 1.19, the count is the number of valid records (i.e. where the token is not an error code) where the tokens are not distinct (calculated as VALID_N minus VALID_DISTINCT_N).

Percent is calculated as the number of distinct IDs with exceptions (shown) divided by the total distinct IDs in the table (not shown). For example, if the DIAGNOSIS table has 2 orphan PATIDs (source table: XTBL_L3_MISMATCH) and 100 distinct PATIDs (source table: DEM_L3_N), the percentage of distinct IDs is 2.0%. For Data Check 1.19, the percent is calculated as 100%-valid_distinct_n_pct.

Source tables include all potential source tables for the data check calculations.

Sample data

Table IIE. Orphan Records, Replication Errors, Encounter Duplication and Hash Token Duplication (continued - page 4 of 4)

This table illustrates exceptions to Data Checks 1.08 (tables contain orphan PATIDs), 1.09 (tables contain orphan ENCOUNTERIDs for more than 5% of records), 1.10 (replication errors between the ENCOUNTER, PROCEDURES and DIAGNOSIS tables), 1.11 (more than 5% of encounters are assigned to more than one patient), 1.12 (tables contain orphan PROVIDERIDs), 1.14 (patients are missing from HASH_TOKEN), and 1.19 (more than 10% of hash tokens are assigned to multiple patients). Orphan PATIDs are not present in the DEMOGRAPHIC table. Orphan ENCOUNTERIDs are not present in the ENCOUNTER table. Orphan PROVIDERIDs are not present in the PROVIDER table. Replication errors are ENCOUNTERIDs in the DIAGNOSIS or PROCEDURES table where the encounter type or admit date does not match the corresponding value in the ENCOUNTER table. Data check exceptions to 1.14 and 1.19 are highlighted in blue and must be explained in the ETL ADD; all other data check exceptions are highlighted in red and must be corrected.

Data Check	Data Check Description	Exception	Table(s)	Field(s)	Count	%	Source table(s)
			HASH_TOKEN	TOKEN_15	0		
			HASH_TOKEN	TOKEN_16	0		
			HASH_TOKEN	TOKEN_17	0		
			HASH_TOKEN	TOKEN_18	0		
			HASH_TOKEN	TOKEN_23	0		
			HASH_TOKEN	TOKEN_24	0		
			HASH_TOKEN	TOKEN_25	0		
			HASH_TOKEN	TOKEN_26	0		
			HASH_TOKEN	TOKEN_29	0		
			HASH_TOKEN	TOKEN_30	0		

Sample data

For Data Checks 1.08, 1.09, 1.11 and 1.12, the count is the number of distinct IDs with an exception. For Data Check 1.10, the count is the number of records with replication errors. For Data Check 1.19, the count is the number of valid records (i.e. where the token is not an error code) where the tokens are not distinct (calculated as VALID_N minus VALID_DISTINCT_N).

Percent is calculated as the number of distinct IDs with exceptions (shown) divided by the total distinct IDs in the table (not shown). For example, if the DIAGNOSIS table has 2 orphan PATIDs (source table: XTBL_L3_MISMATCH) and 100 distinct PATIDs (source table: DEM_L3_N), the percentage of distinct IDs is 2.0%. For Data Check 1.19, the percent is calculated as 100%-valid_distinct_n_pct.

Source tables include all potential source tables for the data check calculations.

Table IIF. Potential Code Errors

This table illustrates exceptions to Data Check 1.13 (more than 5% of ICD, CPT, LOINC, RXCUI, or NDC codes do not conform to the expected length or content) and 1.16 (laboratory results or clinical observations are recorded in the wrong table). Results will be displayed for the code types observed in the data. Exceptions to Data Check 1.13 are highlighted in red and must be corrected. The CDM specification provides guidance on addressing potential code type errors (see General Implementation Guidance issue #5). Exceptions to Data Check 1.16 are highlighted in blue and must be explained in the ETL ADD.

Table	Code Type	Distinct Codes	Records	Records with Code Type Errors	Percent of Records with Code Type Errors (Data Check 1.13)	Records in the Wrong Table	Percentage of Records in the Wrong Table (Data Check 1.16)
DIAGNOSIS	09	3582	28,799	578	2.01	0	0.00
DISPENSING	ND	12323	12,323	0	0.00	0	0.00
IMMUNIZATION	CH	10	41,523	0	0.00	0	0.00
IMMUNIZATION	CX	10	41,791	0	0.00	0	0.00
IMMUNIZATION	ND	10	41,747	0	0.00	0	0.00
IMMUNIZATION	RX	10	41,506	0	0.00	0	0.00
LAB_RESULT_CM	LC	9	10,508	4	0.04	0	0.00
MED_ADMIN	ND	1795	1,795	33	1.84	0	0.00
MED_ADMIN	RX	50	1,614	0	0.00	0	0.00
OBS_CLIN	LC	288	1,286	0	0.00	0	0.00
PRESCRIBING	RX	50	7,409	0	0.00	0	0.00
PROCEDURES	09	2025	18,470	17,102	**92.59**	0	0.00
PROCEDURES	10	1651	19,375	19,375	**100.00**	0	0.00
PROCEDURES	CH	1620	17,312	359	2.07	0	0.00
PROCEDURES	ND	1676	19,069	19,069	**100.00**	0	0.00

Sample data

Results are produced by the potential_code_errors program. If errors are present, see the Potential Code Errors PDF and the SAS datasets produced by this program (see the Potential Code Errors section of the Data Curation Query Workplan for a list of the datasets).

Table IIG. LOINC Panel Codes

This table illustrates exceptions to Data Check 1.20 (more than 5% of LOINC records in the LAB_RESULT_CM, PRO_CM, and OBS_CLIN tables are panel codes). Exceptions are highlighted in blue and must be explained in the ETL ADD.

Table	Field	LOINC records with a Panel_Type of 'Panel'	LOINC records	% of LOINC records that are panels	Source table(s)
LAB_RESULT_CM	LAB_LOINC	0	10,508	0.00	LAB_L3_LOINC
OBS_CLIN	OBSCLIN_CODE	1,011	1,286	**78.62**	OBSCLIN_L3_LOINC
PRO_CM	PRO_ITEM_LOINC	0	0		PROCM_L3_LOINC

Sample data

Table IIIA. Future Dates

This table includes most but not all CDM date fields and supports Data Check 2.01 (more than 5% of records have future dates). Future dates are calculated as those with dates occurring after the maximum refresh date. Future dates may be attributable to data entry errors in the source data or ETL errors such as incorrect REFRESH dates or including scheduled appointments in the ENCOUNTER table. Data check exceptions are highlighted in blue and should be investigated and explained in the ETL ADD.

		Records with future dates			
Table	Field	Numerator	Denominator	%	Source table(s)
DEMOGRAPHIC	BIRTH_DATE	0	3,644	0.00	XTBL_L3_DATES
DEATH	DEATH_DATE	0	1,191	0.00	XTBL_L3_DATES
ENCOUNTER	ADMIT_DATE	0	107,044	0.00	XTBL_L3_DATES
ENCOUNTER	DISCHARGE_DATE	0	104,935	0.00	XTBL_L3_DATES
DIAGNOSIS	ADMIT_DATE	0	31,356	0.00	XTBL_L3_DATES
DIAGNOSIS	DX_DATE	0	31,383	0.00	XTBL_L3_DATES
PROCEDURES	ADMIT_DATE	19,999	182,893	**10.93**	XTBL_L3_DATES
PROCEDURES	PX_DATE	19,999	182,822	**10.94**	XTBL_L3_DATES
VITAL	MEASURE_DATE	0	3,765	0.00	XTBL_L3_DATES
PRESCRIBING	RX_ORDER_DATE	0	7,433	0.00	XTBL_L3_DATES
PRESCRIBING	RX_START_DATE	2,999	7,478	**40.10**	XTBL_L3_DATES
DISPENSING	DISPENSE_DATE	0	12,323	0.00	XTBL_L3_DATES
LAB_RESULT_CM	LAB_ORDER_DATE	0	10,308	0.00	XTBL_L3_DATES
LAB_RESULT_CM	SPECIMEN_DATE	0	10,319	0.00	XTBL_L3_DATES
LAB_RESULT_CM	RESULT_DATE	0	10,508	0.00	XTBL_L3_DATES
HARVEST	REFRESH_MAX	0			XTBL_L3_METADATA
MED_ADMIN	MEDADMIN_START_DATE	0	4,349	0.00	XTBL_L3_DATES
MED_ADMIN	MEDADMIN_STOP_DATE	0	4,268	0.00	XTBL_L3_DATES

Sample data

The numerator is the number of records with dates after the maximum DataMart refresh date (see REFRESH_MAX in the XTBL_L3_METADATA table). The denominator is the number of records with a populated date.

Table IIIA. Future Dates (continued)

This table includes most but not all CDM date fields and supports Data Check 2.01 (more than 5% of records have future dates). Future dates are calculated as those with dates occurring after the maximum refresh date. Future dates may be attributable to data entry errors in the source data or ETL errors such as incorrect REFRESH dates or including scheduled appointments in the ENCOUNTER table. Data check exceptions are highlighted in blue and should be investigated and explained in the ETL ADD.

		Records with future dates			
Table	Field	Numerator	Denominator	%	Source table(s)
OBS_CLIN	OBSCLIN_START_DATE	0	3,354	0.00	XTBL_L3_DATES
OBS_CLIN	OBSCLIN_STOP_DATE	0	3,282	0.00	XTBL_L3_DATES
IMMUNIZATION	VX_RECORD_DATE	0	162,990	0.00	XTBL_L3_DATES
IMMUNIZATION	VX_ADMIN_DATE	0	162,886	0.00	XTBL_L3_DATES

Sample data

The numerator is the number of records with dates after the maximum DataMart refresh date (see REFRESH_MAX in the XTBL_L3_METADATA table). The denominator is the number of records with a populated date.

Table IIIB. Records With Extreme Values

This table supports Data Check 2.02 (more than 10% of records fall into the lowest or highest categories of age, height, weight, diastolic blood pressure, systolic blood pressure, or dispensed days supply). A high percentage of records in these categories may signal incorrect measurement units. Exceptions for blood pressure measures are expected for primarily pediatric populations. Data check exceptions are highlighted in blue and should be investigated and explained in the ETL ADD.

		Data Check Parameters			Records with values in the lowest category		Records with values in the highest category			
Table	Field	Low	High	Records	N	%	N	%	Median	Source table
DEMOGRAPHIC	AGE (derived from BIRTH_DATE)	<0 yrs.	>89 yrs.	3,644	15	0.4	0	0.0	24	DEM_L3_AGEYRSDIST2; DEM_L3_AGEYRSDIST1
VITAL	HT	<0 inches	>=95 inches	3,686	0	0.0	52	1.4	65	VIT_L3_HT; VIT_L3_HT_DIST
VITAL	WT	<0 lbs.	>350 lbs.	3,714	0	0.0	1,050	**28.3**	173	VIT_L3_WT; VIT_L3_WT_DIST
VITAL	DIASTOLIC	<40 mgHg	>120 mgHg	3,696	65	1.8	477	**12.9**	n/a	VIT_L3_DIASTOLIC
VITAL	SYSTOLIC	<40 mgHg	>210 mgHg	3,698	83	2.2	71	1.9	n/a	VIT_L3_SYSTOLIC
DISPENSING	DISPENSE_SUP_GROUP	<1 day	>90 days	12,192	472	3.9	5,184	**42.5**	n/a	DISP_L3_SUPDIST2

Table excludes records with values outside of CDM specifications.

Sample data

Table IIIC. Illogical Dates

This table shows the number of patients who have dates of service in the DEATH, DISPENSING, ENCOUNTER, LAB_RESULT_CM, PRESCRIBING, PROCEDURES, VITAL, MED_ADMIN, OBS_CLIN, or OBS_GEN table which occur before their birth date or after their death date; the number of patients who have procedure dates occurring more than 5 days before the admit date or more than five days after the discharge date for the same encounter in the PROCEDURES table; and the number of patients who have a start date before the end date in the OBS_CLIN and OBS_GEN tables. The table shows these numbers as a percentage of patients in the ENCOUNTER table. Although some patients may not be included in the ENCOUNTER table, patients with encounters are the most relevant denominator for this table. This table supports Data Check 2.03 (more than 5% of patients have illogical date relationships). Data check exceptions are highlighted in blue and should be investigated and explained in the ETL ADD.

DATE_COMPARISON	Patients	Percentage of total patients in the ENCOUNTER table	Source tables
ADMIT_DATE < BIRTH_DATE	92	2.3	XTBL_L3_DATE_LOGIC;ENC_L3_N
DISCHARGE_DATE < BIRTH_DATE	92	2.3	XTBL_L3_DATE_LOGIC;ENC_L3_N
PX_DATE < BIRTH_DATE	90	2.2	XTBL_L3_DATE_LOGIC;ENC_L3_N
DX_DATE < BIRTH_DATE	75	1.9	XTBL_L3_DATE_LOGIC;ENC_L3_N
MEASURE_DATE < BIRTH_DATE	29	0.7	XTBL_L3_DATE_LOGIC;ENC_L3_N
DISPENSE_DATE < BIRTH_DATE	91	2.3	XTBL_L3_DATE_LOGIC;ENC_L3_N
RX_START_DATE < BIRTH_DATE	57	1.4	XTBL_L3_DATE_LOGIC;ENC_L3_N
RESULT_DATE < BIRTH_DATE	83	2.1	XTBL_L3_DATE_LOGIC;ENC_L3_N
DEATH_DATE < BIRTH_DATE	21	0.5	XTBL_L3_DATE_LOGIC;ENC_L3_N
MEDADMIN_START_DATE < BIRTH_DATE	73	1.8	XTBL_L3_DATE_LOGIC;ENC_L3_N
OBSCLIN_START_DATE < BIRTH_DATE	54	1.3	XTBL_L3_DATE_LOGIC;ENC_L3_N
OBSGEN_START_DATE < BIRTH_DATE	0	0.0	XTBL_L3_DATE_LOGIC;ENC_L3_N
VX_RECORD_DATE < BIRTH_DATE	91	2.3	XTBL_L3_DATE_LOGIC;ENC_L3_N
ADMIT_DATE > DEATH_DATE	161	4.0	XTBL_L3_DATE_LOGIC;ENC_L3_N
DISCHARGE_DATE > DEATH_DATE	143	3.5	XTBL_L3_DATE_LOGIC;ENC_L3_N
PX_DATE > DEATH_DATE	155	3.8	XTBL_L3_DATE_LOGIC;ENC_L3_N
DX_DATE > DEATH_DATE	171	4.2	XTBL_L3_DATE_LOGIC;ENC_L3_N
MEASURE_DATE > DEATH_DATE	278	**6.9**	XTBL_L3_DATE_LOGIC;ENC_L3_N
DISPENSE_DATE > DEATH_DATE	329	**8.1**	XTBL_L3_DATE_LOGIC;ENC_L3_N
RX_START_DATE > DEATH_DATE	513	**12.7**	XTBL_L3_DATE_LOGIC;ENC_L3_N
RESULT_DATE > DEATH_DATE	378	**9.4**	XTBL_L3_DATE_LOGIC;ENC_L3_N
MEDADMIN_START_DATE > DEATH_DATE	536	**13.3**	XTBL_L3_DATE_LOGIC;ENC_L3_N
OBSCLIN_START_DATE > DEATH_DATE	436	**10.8**	XTBL_L3_DATE_LOGIC;ENC_L3_N

Sample data

Table IIIC. Illogical Dates (continued)

This table shows the number of patients who have dates of service in the DEATH, DISPENSING, ENCOUNTER, LAB_RESULT_CM, PRESCRIBING, PROCEDURES, VITAL, MED_ADMIN, OBS_CLIN, or OBS_GEN table which occur before their birth date or after their death date; the number of patients who have procedure dates occurring more than 5 days before the admit date or more than five days after the discharge date for the same encounter in the PROCEDURES table; and the number of patients who have a start date before the end date in the OBS_CLIN and OBS_GEN tables. The table shows these numbers as a percentage of patients in the ENCOUNTER table. Although some patients may not be included in the ENCOUNTER table, patients with encounters are the most relevant denominator for this table. This table supports Data Check 2.03 (more than 5% of patients have illogical date relationships). Data check exceptions are highlighted in blue and should be investigated and explained in the ETL ADD.

DATE_COMPARISON	Patients	Percentage of total patients in the ENCOUNTER table	Source tables
OBSGEN_START_DATE > DEATH_DATE	0	0.0	XTBL_L3_DATE_LOGIC;ENC_L3_N
VX_RECORD_DATE > DEATH_DATE	119	2.9	XTBL_L3_DATE_LOGIC;ENC_L3_N
ADMIT_DATE > DISCHARGE_DATE	3,685	**91.2**	XTBL_L3_DATE_LOGIC;ENC_L3_N
PX_DATE is More Than 5 Days Prior To The ADMIT_DATE	3,462	**85.7**	XTBL_L3_DATE_LOGIC;ENC_L3_N
PX_DATE is More Than 5 Days After The DISCHARGE_DATE	0	0.0	XTBL_L3_DATE_LOGIC;ENC_L3_N
DX_DATE is More Than 5 Days Prior To The ADMIT_DATE	2,554	**63.2**	XTBL_L3_DATE_LOGIC;ENC_L3_N
DX_DATE is More Than 5 Days After The DISCHARGE_DATE	0	0.0	XTBL_L3_DATE_LOGIC;ENC_L3_N
OBSCLIN_START_DATE > OBSCLIN_STOP_DATE	1,507	**37.3**	XTBL_L3_DATE_LOGIC;ENC_L3_N

Sample data

Table IIID. Encounters Per Visit and Per Patient

This table shows the number of encounters, patients, encounters per patient, visits (unique encounters per patient, provider, encounter type, and day), and encounters per visit by encounter type. These data support Data Check 2.04 (the average number of encounters per visit is > 2.0 for inpatient (IP), emergency department (ED), or ED to inpatient (EI) encounters). A high number of encounters per visit may signal potential redundancy or duplication. Data check exceptions are highlighted in blue and should be investigated and explained in the ETL ADD.

Encounter Type	Encounters	Patients	Encounters per Patient	Encounters with known PROVIDERID	Visit (unique combinations of PATID, ENC_TYPE, ADMIT_DATE, and PROVIDERID)	Encounters with known PROVIDERID per visit	Source table
AV (Ambulatory Visit)	10,682	2,577	4.1	10,575	10,562	1.00	ENC_L3_ENCTYPE
ED (Emergency Dept)	10,649	2,590	4.1	10,532	10,516	1.00	ENC_L3_ENCTYPE
EI (ED to IP Stay)	10,918	2,589	4.2	10,821	10,805	1.00	ENC_L3_ENCTYPE
IC (Institutional Professional Consult)	10,734	2,611	4.1	10,615	10,603	1.00	ENC_L3_ENCTYPE
IP (Inpatient Hospital Stay)	10,748	2,563	4.2	10,642	10,633	1.00	ENC_L3_ENCTYPE
IS (Non-acute Institutional Stay)	10,731	2,569	4.2	10,626	10,615	1.00	ENC_L3_ENCTYPE
OA (Other Ambulatory Visit)	10,691	2,551	4.2	10,580	10,570	1.00	ENC_L3_ENCTYPE
OS (Observation Stay)	10,828	2,562	4.2	10,717	10,699	1.00	ENC_L3_ENCTYPE
TH (Telehealth)	10,603	2,576	4.1	10,474	10,466	1.00	ENC_L3_ENCTYPE
Missing, NI, UN or OT	10,460	4,784	2.2	10,356	10,351	1.00	ENC_L3_ENCTYPE

Sample data

Table IIIG. Monthly Record Volume Outliers, Selected Domains

This table displays data check exceptions for Data Check 2.08 (the monthly volume of encounter, diagnosis, procedure, vital, prescribing, or laboratory records is an outlier). Encounter types of interest are AV (Ambulatory Visit), ED (Emergency Department), EI (Emergency Department Admit to Inpatient Hospital Stay), and IP (Inpatient Hospital Stay). The Evaluation Window^ is shown in the table. Outliers are identified by computing a Difference Ratio^^ of each month compared to statistics of the previous 12 months. An outlier is defined as 0 records or a Difference Ratio of -7.0 or less. Months are excluded from the data check calculation if (a) the average record count during the previous 12 months was <500 or (b) the standard deviation during the previous 12 months was 0. If the exception occurs within the 60 months prior to the end of the Evaluation Window, the data check exception will be graphically compared to monthly record volumes in Charts IIIA through Chart IIIF. Data check exceptions are highlighted in blue and should be investigated and explained in the ETL ADD.

Table	Evaluation Window^	Encounter Type	Exception Month	Records	Average Records in the Previous 12 Months	Standard Deviation in the Previous 12 Months	Difference Ratio^^	Source table
	No Data Table contains a Data Check Exception							

Sample data

^ The Evaluation Window is determined as follows. The most recent twelve months are excluded because they are already evaluated by the data latency checks (Data Checks 3.07 and 3.11). Months prior to the 5th percentile for the Encounter Admission Date, Rx Order Date, Lab Result Date, Med Admin Start Date and Vital Measurement Date are excluded since volumes may be unstable in these early months.

^^ The Difference Ratio is calculated as the volume in the current month minus the average volume in the previous 12 months divided by the standard deviation during the previous 12 months.

Table IVA. Diagnosis Records Per Encounter and Per Patient, Overall and by Encounter Type

This table supports Data Check 3.01 (the average number of diagnoses records with known diagnosis types per encounter is below threshold [1.0 for ambulatory (AV), inpatient (IP), emergency department (ED), ED to inpatient (EI) encounters, or telehealth (TH) encounters]). Data check exceptions are highlighted in blue and should be investigated and explained in the ETL ADD.

Encounter Type	DIAGNOSIS records	DIAGNOSIS records with known DX_TYPE	ENCOUNTER records	Diagnosis records per encounter	Diagnosis records with known DX_TYPE per encounter	Source table
AV (Ambulatory Visit)	3,077	2,848	10,682	0.29	**0.27**	DIA_L3_ENCTYPE; DIA_L3_DXTYPE_ENCTYPE; ENC_L3_ENCTYPE
ED (Emergency Dept)	3,146	2,872	10,649	0.30	**0.27**	DIA_L3_ENCTYPE; DIA_L3_DXTYPE_ENCTYPE; ENC_L3_ENCTYPE
EI (ED to IP Stay)	3,152	2,887	10,918	0.29	**0.26**	DIA_L3_ENCTYPE; DIA_L3_DXTYPE_ENCTYPE; ENC_L3_ENCTYPE
IC (Institutional Professional Consult)	3,087	2,829	10,734	0.29	0.26	DIA_L3_ENCTYPE; DIA_L3_DXTYPE_ENCTYPE; ENC_L3_ENCTYPE
IP (Inpatient Hospital Stay)	3,041	2,787	10,748	0.28	**0.26**	DIA_L3_ENCTYPE; DIA_L3_DXTYPE_ENCTYPE; ENC_L3_ENCTYPE
IS (Non-acute Institutional Stay)	3,213	2,943	10,731	0.30	0.27	DIA_L3_ENCTYPE; DIA_L3_DXTYPE_ENCTYPE; ENC_L3_ENCTYPE
OA (Other Ambulatory Visit)	3,066	2,800	10,691	0.29	0.26	DIA_L3_ENCTYPE; DIA_L3_DXTYPE_ENCTYPE; ENC_L3_ENCTYPE
OS (Observation Stay)	3,102	2,844	10,828	0.29	0.26	DIA_L3_ENCTYPE; DIA_L3_DXTYPE_ENCTYPE; ENC_L3_ENCTYPE
TH (Telehealth)	3,181	2,905	10,603	0.30	**0.27**	DIA_L3_ENCTYPE; DIA_L3_DXTYPE_ENCTYPE; ENC_L3_ENCTYPE
Missing, NI, UN or OT	3,966	3,602	10,460	0.38	0.34	DIA_L3_ENCTYPE; DIA_L3_DXTYPE_ENCTYPE; ENC_L3_ENCTYPE
Total	32,031	29,317	107,044	0.30	0.27	DIA_L3_ENCTYPE; DIA_L3_DXTYPE_ENCTYPE; ENC_L3_ENCTYPE

Sample data

The denominator comes from the ENCOUNTER table.

Table IVB. Procedure Records Per Encounter and Per Patient, Overall and by Encounter Type

This table supports Data Check 3.02 (the average number of procedure records with known procedure types per encounter is below threshold [0.75 for ambulatory (AV) encounters, 0.75 for emergency department (ED) encounters, 1.00 for ED to inpatient (EI) encounters, and 1.00 for inpatient (IP) encounters]). Data check exceptions are highlighted in blue and should be investigated and explained in the ETL ADD.

Encounter Type	PROCEDURES records	PROCEDURES records with known PX_TYPE	ENCOUNTER records	Procedures records per encounter	Procedures records with known PX_TYPE per encounter	Source table
AV (Ambulatory Visit)	18,114	13,561	10,682	1.70	1.27	PRO_L3_ENCTYPE; PRO_L3_PXTYPE_ENCTYPE; ENC_L3_ENCTYPE
ED (Emergency Dept)	18,156	13,498	10,649	1.70	1.27	PRO_L3_ENCTYPE; PRO_L3_PXTYPE_ENCTYPE; ENC_L3_ENCTYPE
EI (ED to IP Stay)	18,125	13,543	10,918	1.66	1.24	PRO_L3_ENCTYPE; PRO_L3_PXTYPE_ENCTYPE; ENC_L3_ENCTYPE
IC (Institutional Professional Consult)	18,135	13,495	10,734	1.69	1.26	PRO_L3_ENCTYPE; PRO_L3_PXTYPE_ENCTYPE; ENC_L3_ENCTYPE
IP (Inpatient Hospital Stay)	18,099	13,574	10,748	1.68	1.26	PRO_L3_ENCTYPE; PRO_L3_PXTYPE_ENCTYPE; ENC_L3_ENCTYPE
IS (Non-acute Institutional Stay)	18,295	13,560	10,731	1.70	1.26	PRO_L3_ENCTYPE; PRO_L3_PXTYPE_ENCTYPE; ENC_L3_ENCTYPE
OA (Other Ambulatory Visit)	18,124	13,486	10,691	1.70	1.26	PRO_L3_ENCTYPE; PRO_L3_PXTYPE_ENCTYPE; ENC_L3_ENCTYPE
OS (Observation Stay)	18,198	13,641	10,828	1.68	1.26	PRO_L3_ENCTYPE; PRO_L3_PXTYPE_ENCTYPE; ENC_L3_ENCTYPE
TH (Telehealth)	18,094	13,563	10,603	1.71	1.28	PRO_L3_ENCTYPE; PRO_L3_PXTYPE_ENCTYPE; ENC_L3_ENCTYPE
Missing, NI, UN or OT	22,765	16,991	10,460	2.18	1.62	PRO_L3_ENCTYPE; PRO_L3_PXTYPE_ENCTYPE; ENC_L3_ENCTYPE
Total	186,105	138,912	107,044	1.74	1.30	PRO_L3_ENCTYPE; PRO_L3_PXTYPE_ENCTYPE; ENC_L3_ENCTYPE

The denominator comes from the ENCOUNTER table.

Sample data

Table IVC. Missing or Unknown Values, Required Tables

This table includes fields in the DEMOGRAPHIC, ENROLLMENT, ENCOUNTER, DIAGNOSIS, and PROCEDURES tables where the field is included in the query results and is not required to be populated. The table depicts the percentage of records with missing or unknown values. Results support Data Check 3.03 (more than 10% of records have missing or unknown values) for the following fields: BIRTH_DATE, SEX, DISCHARGE_DATE (IP/EI encounters only), DISCHARGE_DISPOSITION (IP/EI encounters only), DX_ORIGIN, DX_SOURCE, DX_TYPE, DIAGNOSIS.ENCOUNTERID, PX_DATE, PX_SOURCE, PX_TYPE, and PROCEDURES.ENCOUNTERID. Data check exceptions are highlighted in blue and should be investigated and explained in the ETL ADD.

			Records with missing, NI, UN, or OT values			
Table	Field	Encounter Type Constraint	Numerator	Denominator	%	Source table
DEMOGRAPHIC	BIRTH_DATE		0	3,644		XTBL_L3_DATES
DEMOGRAPHIC	BIRTH_TIME		382	3,644	10.5	XTBL_L3_TIMES
DEMOGRAPHIC	SEX		910	3,644	**25.0**	DEM_L3_SEXDIST
DEMOGRAPHIC	HISPANIC		421	3,644	11.6	DEM_L3_HISPDIST
DEMOGRAPHIC	RACE		382	3,644	10.5	DEM_L3_RACEDIST
DEMOGRAPHIC	GENDER_IDENTITY		874	3,644	24.0	DEM_L3_GENDERDIST
DEMOGRAPHIC	SEXUAL_ORIENTATION		160	3,644	4.4	DEM_L3_ORIENTDIST
DEMOGRAPHIC	PAT_PREF_LANGUAGE_ SPOKEN		190	3,644	5.2	DEM_L3_PATPREFLANG
ENROLLMENT	ENR_END_DATE		0			XTBL_L3_DATES
ENROLLMENT	CHART		0			ENR_L3_CHART
ENCOUNTER	ADMIT_TIME		11,542	107,044	10.8	XTBL_L3_TIMES
ENCOUNTER	DISCHARGE_DATE	IP or EI	426	21,666	2.0	ENC_L3_ENCTYPE_DDATE_YM
ENCOUNTER	DISCHARGE_TIME		105,989	107,044	99.0	XTBL_L3_TIMES
ENCOUNTER	ENC_TYPE		10,460	107,044	9.8	ENC_L3_ENCTYPE
ENCOUNTER	PROVIDERID		1,106	107,044	1.0	ENC_L3_N
ENCOUNTER	FACILITYID		102,973	107,044	96.2	ENC_L3_N
ENCOUNTER	DISCHARGE_DISPOSITION	IP or EI	21,549	21,666	**99.5**	ENC_L3_ENCTYPE_DISDISP
ENCOUNTER	DISCHARGE_STATUS	IP or EI	21,475	21,666	99.1	ENC_L3_ENCTYPE_DISSTAT

Sample data

Table excludes records with values outside of CDM specifications.
The four 'flavors of null' defined in the CDM are combined here but details are available in the source tables.
The denominator is derived from the applicable _N query (ALL_N + NULL_N for TAG=PATID) or the specific query for measures with encounter type constraints.

Table IVC. Missing or Unknown Values, Required Tables (continued - page 2 of 3)

This table includes fields in the DEMOGRAPHIC, ENROLLMENT, ENCOUNTER, DIAGNOSIS, and PROCEDURES tables where the field is included in the query results and is not required to be populated. The table depicts the percentage of records with missing or unknown values. Results support Data Check 3.03 (more than 10% of records have missing or unknown values) for the following fields: BIRTH_DATE, SEX, DISCHARGE_DATE (IP/EI encounters only), DISCHARGE_DISPOSITION (IP/EI encounters only), DX_ORIGIN, DX_SOURCE, DX_TYPE, DIAGNOSIS.ENCOUNTERID, PX_DATE, PX_SOURCE, PX_TYPE, and PROCEDURES.ENCOUNTERID. Data check exceptions are highlighted in blue and should be investigated and explained in the ETL ADD.

			Records with missing, NI, UN, or OT values			
Table	Field	Encounter Type Constraint	Numerator	Denominator	%	Source table
ENCOUNTER	DRG	IP or EI	21,514	21,666	99.3	ENC_L3_ENCTYPE_DRG
ENCOUNTER	ADMITTING_SOURCE	IP or EI	2,615	21,666	12.1	ENC_L3_ENCTYPE_ADMSRC
ENCOUNTER	PAYER_TYPE_PRIMARY		5,008	107,044	4.7	ENC_L3_PAYERTYPE1
ENCOUNTER	PAYER_TYPE_SECONDARY		5,028	107,044	4.7	ENC_L3_PAYERTYPE2
ENCOUNTER	FACILITY_TYPE		6,926	107,044	6.5	ENC_L3_FACILITYTYPE
ENCOUNTER	FACILITY_LOCATION		0	107,044		ENC_L3_FACILITYLOC
DIAGNOSIS	DX_DATE		648	32,031	2.0	XTBL_L3_DATES
DIAGNOSIS	DX_TYPE		2,714	32,031	8.5	DIA_L3_DXTYPE
DIAGNOSIS	DX_SOURCE		2,540	32,031	7.9	DIA_L3_DXSOURCE
DIAGNOSIS	DX_ORIGIN		3,990	32,031	**12.5**	DIA_L3_ORIGIN
DIAGNOSIS	PDX	IP or EI	2,862	6,193	46.2	DIA_L3_PDX_ENCTYPE
DIAGNOSIS	ENCOUNTERID		0	32,031		DIA_L3_N
DIAGNOSIS	ADMIT_DATE		675	32,031	2.1	XTBL_L3_DATES
DIAGNOSIS	ENC_TYPE		3,966	32,031	12.4	DIA_L3_ENCTYPE
DIAGNOSIS	DX_POA		14,231	32,031	44.4	DIA_L3_DXPOA
DIAGNOSIS	PROVIDERID		313	32,031	1.0	DIA_L3_N
PROCEDURES	PX_DATE		3,283	186,105	1.8	XTBL_L3_DATES
PROCEDURES	PX_TYPE		47,193	186,105	**25.4**	PRO_L3_PXTYPE
PROCEDURES	PX_SOURCE		7,507	186,105	4.0	PRO_L3_PXSOURCE
PROCEDURES	ENCOUNTERID		0	186,105		PRO_L3_N
PROCEDURES	PPX		124,245	186,105	66.8	PRO_L3_PPX
PROCEDURES	PROVIDERID		1,905	186,105	1.0	PRO_L3_N

Sample data

Table excludes records with values outside of CDM specifications.
The four 'flavors of null' defined in the CDM are combined here but details are available in the source tables.
The denominator is derived from the applicable _N query (ALL_N + NULL_N for TAG=PATID) or the specific query for measures with encounter type constraints.

Table IVC. Missing or Unknown Values, Required Tables (continued - page 3 of 3)

This table includes fields in the DEMOGRAPHIC, ENROLLMENT, ENCOUNTER, DIAGNOSIS, and PROCEDURES tables where the field is included in the query results and is not required to be populated. The table depicts the percentage of records with missing or unknown values. Results support Data Check 3.03 (more than 10% of records have missing or unknown values) for the following fields: BIRTH_DATE, SEX, DISCHARGE_DATE (IP/EI encounters only), DISCHARGE_DISPOSITION (IP/EI encounters only), DX_ORIGIN, DX_SOURCE, DX_TYPE, DIAGNOSIS.ENCOUNTERID, PX_DATE, PX_SOURCE, PX_TYPE, and PROCEDURES.ENCOUNTERID. Data check exceptions are highlighted in blue and should be investigated and explained in the ETL ADD.

			Records with missing, NI, UN, or OT values			
Table	Field	Encounter Type Constraint	Numerator	Denominator	%	Source table
PROCEDURES	ADMIT_DATE		3,212	186,105	1.7	XTBL_L3_DATES
PROCEDURES	ENC_TYPE		22,765	186,105	12.2	PRO_L3_ENCTYPE

Sample data

Table excludes records with values outside of CDM specifications.
The four 'flavors of null' defined in the CDM are combined here but details are available in the source tables.
The denominator is derived from the applicable _N query (ALL_N + NULL_N for TAG=PATID) or the specific query for measures with encounter type constraints.

Table IVD. Missing or Unknown Values, Optional Tables

This table includes most of the fields in the VITAL, DEATH, LAB_RESULT_CM, PRESCRIBING, DISPENSING, CONDITION, DEATH_CAUSE, PROVIDER, MED_ADMIN, OBS_CLIN and OBS_GEN tables where the field is included in the query results and is not required to be populated. Vital measures (e.g. HEIGHT) are not included because the table structure does not support missingness assessment. The table depicts the percentage of records with missing or unknown values. Results support Data Check 3.03 (more than 10% of records have missing or unknown values) for the following fields: RX_ORDER_DATE, VX_RECORD_DATE, DISPENSE_SUP, DEATH_SOURCE, DISPENSE_SOURCE, CONDITION_SOURCE, LAB_RESULT_SOURCE, MEDADMIN_SOURCE, RX_SOURCE, VITAL_SOURCE, VX_SOURCE, VITAL.ENCOUNTERID, MED_ADMIN.ENCOUNTERID, LAB_RESULT_CM.ENCOUNTERID, MEDADMIN_CODE, MEDADMIN_TYPE, OBSCLIN_CODE, OBSCLIN_TYPE, OBSGEN_CODE, OBSGEN_TYPE. Exceptions are highlighted in blue and should be investigated and explained in the ETL ADD.

		Records with missing, NI, UN, or OT values			
Table	Field	Numerator	Denominator	%	Source table
VITAL	ENCOUNTERID	0	3,765		VIT_L3_N
VITAL	VITAL_SOURCE	553	3,765	**14.7**	VIT_L3_VITAL_SOURCE
VITAL	MEASURE_TIME	420	3,765	11.2	XTBL_L3_TIMES
DEATH	DEATH_DATE_IMPUTE	138	1,216	11.3	DEATH_L3_IMPUTE
DEATH	DEATH_MATCH_CONFIDENCE	341	1,216	28.0	DEATH_L3_MATCH
DEATH	DEATH_DATE	25	1,216	2.1	XTBL_L3_DATES
DEATH	DEATH_SOURCE	98	1,216	8.1	DEATH_L3_SOURCE
LAB_RESULT_CM	ENCOUNTERID	10,508	10,508	**100.0**	LAB_L3_N
LAB_RESULT_CM	SPECIMEN_SOURCE	10	10,508	0.1	LAB_L3_SOURCE
LAB_RESULT_CM	LAB_LOINC	0	10,508		LAB_L3_LOINC
LAB_RESULT_CM	PRIORITY	390	10,508	3.7	LAB_L3_PRIORITY
LAB_RESULT_CM	RESULT_LOC	413	10,508	3.9	LAB_L3_LOC
LAB_RESULT_CM	LAB_PX_TYPE	1,297	10,508	12.3	LAB_L3_PX_TYPE
LAB_RESULT_CM	LAB_PX	468	10,508	4.5	LAB_L3_PX_PXTYPE
LAB_RESULT_CM	LAB_ORDER_DATE	200	10,508	1.9	XTBL_L3_DATES
LAB_RESULT_CM	SPECIMEN_DATE	189	10,508	1.8	XTBL_L3_DATES
LAB_RESULT_CM	SPECIMEN_TIME	396	10,508	3.8	XTBL_L3_TIMES
LAB_RESULT_CM	RESULT_TIME	475	10,508	4.5	XTBL_L3_TIMES

Table excludes records with values outside of CDM specifications.
The four 'flavors of null' defined in the CDM are combined here but details are available in the source tables.
The denominator is derived from the applicable _N query (ALL_N + NULL_N for TAG=PATID).

Sample data

Table IVD. Missing or Unknown Values, Optional Tables (continued - page 2 of 6)
This table includes most of the fields in the VITAL, DEATH, LAB_RESULT_CM, PRESCRIBING, DISPENSING, CONDITION, DEATH_CAUSE, PROVIDER, MED_ADMIN, OBS_CLIN and OBS_GEN tables where the field is included in the query results and is not required to be populated. Vital measures (e.g. HEIGHT) are not included because the table structure does not support missingness assessment. The table depicts the percentage of records with missing or unknown values. Results support Data Check 3.03 (more than 10% of records have missing or unknown values) for the following fields: RX_ORDER_DATE, VX_RECORD_DATE, DISPENSE_SUP, DEATH_SOURCE, DISPENSE_SOURCE, CONDITION_SOURCE, LAB_RESULT_SOURCE, MEDADMIN_SOURCE, RX_SOURCE, VITAL_SOURCE, VX_SOURCE, VITAL.ENCOUNTERID, MED_ADMIN.ENCOUNTERID, LAB_RESULT_CM.ENCOUNTERID, MEDADMIN_CODE, MEDADMIN_TYPE, OBSCLIN_CODE, OBSCLIN_TYPE, OBSGEN_CODE, OBSGEN_TYPE. Exceptions are highlighted in blue and should be investigated and explained in the ETL ADD.

		Records with missing, NI, UN, or OT values			
Table	Field	Numerator	Denominator	%	Source table
LAB_RESULT_CM	RESULT_QUAL	109	10,508	1.0	LAB_L3_QUAL
LAB_RESULT_CM	RESULT_NUM	4,049	10,508	38.5	LAB_L3_LOINC_RESULT_NUM
LAB_RESULT_CM	RESULT_MODIFIER	905	10,508	8.6	LAB_L3_MOD
LAB_RESULT_CM	RESULT_UNIT	6,614	10,508	62.9	LAB_L3_UNIT
LAB_RESULT_CM	NORM_MODIFIER_LOW	1,412	10,508	13.4	LAB_L3_LOW
LAB_RESULT_CM	NORM_MODIFIER_HIGH	741	10,508	7.1	LAB_L3_HIGH
LAB_RESULT_CM	ABN_IND	886	10,508	8.4	LAB_L3_ABN
LAB_RESULT_CM	LAB_LOINC_SOURCE	1,280	10,508	12.2	LAB_L3_LSOURCE
LAB_RESULT_CM	LAB_RESULT_SOURCE	0	10,508		LAB_L3_RSOURCE
LAB_RESULT_CM	RESULT_SNOMED	1,471	10,508	14.0	LAB_L3_SNOMED
PRESCRIBING	ENCOUNTERID	3,731	7,574	**49.3**	PRES_L3_N
PRESCRIBING	RX_PROVIDERID	336	7,574	4.4	PRES_L3_N
PRESCRIBING	RX_ORDER_DATE	141	7,574	1.9	XTBL_L3_DATES
PRESCRIBING	RX_ORDER_TIME	291	7,574	3.8	XTBL_L3_TIMES
PRESCRIBING	RX_START_DATE	96	7,574	1.3	XTBL_L3_DATES
PRESCRIBING	RX_END_DATE	168	7,574	2.2	XTBL_L3_DATES
PRESCRIBING	RX_DAYS_SUPPLY	75	7,574	1.0	PRES_L3_SUPDIST2
PRESCRIBING	RX_FREQUENCY	997	7,574	13.2	PRES_L3_FREQ

Sample data

Table excludes records with values outside of CDM specifications.
The four 'flavors of null' defined in the CDM are combined here but details are available in the source tables.
The denominator is derived from the applicable _N query (ALL_N + NULL_N for TAG=PATID).

Table IVD. Missing or Unknown Values, Optional Tables (continued - page 3 of 6)

This table includes most of the fields in the VITAL, DEATH, LAB_RESULT_CM, PRESCRIBING, DISPENSING, CONDITION, DEATH_CAUSE, PROVIDER, MED_ADMIN, OBS_CLIN and OBS_GEN tables where the field is included in the query results and is not required to be populated. Vital measures (e.g. HEIGHT) are not included because the table structure does not support missingness assessment. The table depicts the percentage of records with missing or unknown values. Results support Data Check 3.03 (more than 10% of records have missing or unknown values) for the following fields: RX_ORDER_DATE, VX_RECORD_DATE, DISPENSE_SUP, DEATH_SOURCE, DISPENSE_SOURCE, CONDITION_SOURCE, LAB_RESULT_SOURCE, MEDADMIN_SOURCE, RX_SOURCE, VITAL_SOURCE, VX_SOURCE, VITAL.ENCOUNTERID, MED_ADMIN.ENCOUNTERID, LAB_RESULT_CM.ENCOUNTERID, MEDADMIN_CODE, MEDADMIN_TYPE, OBSCLIN_CODE, OBSCLIN_TYPE, OBSGEN_CODE, OBSGEN_TYPE. Exceptions are highlighted in blue and should be investigated and explained in the ETL ADD.

		Records with missing, NI, UN, or OT values			
Table	Field	Numerator	Denominator	%	Source table
PRESCRIBING	RX_BASIS	911	7,574	12.0	PRES_L3_BASIS
PRESCRIBING	RXNORM_CUI	165	7,574	2.2	PRES_L3_RXCUI
PRESCRIBING	RX_QUANTITY	78	7,574	1.0	PRES_L3_RXQTY_DIST
PRESCRIBING	RX_REFILLS	134	7,574	1.8	PRES_L3_RXREFILL_DIST
PRESCRIBING	RX_DOSE_ORDERED	75	7,574	1.0	PRES_L3_RXDOSEODR_DIST
PRESCRIBING	RX_DOSE_ORDERED_UNIT	273	7,574	3.6	PRES_L3_RXDOSEODRUNIT
PRESCRIBING	RX_DOSE_FORM	394	7,574	5.2	PRES_L3_RXDOSEFORM
PRESCRIBING	RX_PRN_FLAG	2,619	7,574	34.6	PRES_L3_PRNFLAG
PRESCRIBING	RX_ROUTE	376	7,574	5.0	PRES_L3_ROUTE
PRESCRIBING	RX_SOURCE	4,974	7,574	**65.7**	PRES_L3_SOURCE
PRESCRIBING	RX_DISPENSE_AS_WRITTEN	4,971	7,574	65.6	PRES_L3_DISPASWRTN
DISPENSING	PRESCRIBINGID	12,323	12,323	100.0	DISP_L3_N
DISPENSING	DISPENSE_SUP	131	12,323	1.1	DISP_L3_SUPDIST2
DISPENSING	DISPENSE_DOSE_DISP	111	12,323	0.9	DISP_L3_DOSE_DIST
DISPENSING	DISPENSE_DOSE_DISP_UNIT	399	12,323	3.2	DISP_L3_DOSEUNIT
DISPENSING	DISPENSE_ROUTE	587	12,323	4.8	DISP_L3_ROUTE
DISPENSING	DISPENSE_SOURCE	1,490	12,323	**12.1**	DISP_L3_SOURCE
DISPENSING	DISPENSE_AMT	114	12,323	0.9	DISP_L3_DISPAMT_DIST

Sample data

Table excludes records with values outside of CDM specifications.
The four 'flavors of null' defined in the CDM are combined here but details are available in the source tables.
The denominator is derived from the applicable _N query (ALL_N + NULL_N for TAG=PATID).

Table IVD. Missing or Unknown Values, Optional Tables (continued - page 4 of 6)

This table includes most of the fields in the VITAL, DEATH, LAB_RESULT_CM, PRESCRIBING, DISPENSING, CONDITION, DEATH_CAUSE, PROVIDER, MED_ADMIN, OBS_CLIN and OBS_GEN tables where the field is included in the query results and is not required to be populated. Vital measures (e.g. HEIGHT) are not included because the table structure does not support missingness assessment. The table depicts the percentage of records with missing or unknown values. Results support Data Check 3.03 (more than 10% of records have missing or unknown values) for the following fields: RX_ORDER_DATE, VX_RECORD_DATE, DISPENSE_SUP, DEATH_SOURCE, DISPENSE_SOURCE, CONDITION_SOURCE, LAB_RESULT_SOURCE, MEDADMIN_SOURCE, RX_SOURCE, VITAL_SOURCE, VX_SOURCE, VITAL.ENCOUNTERID, MED_ADMIN.ENCOUNTERID, LAB_RESULT_CM.ENCOUNTERID, MEDADMIN_CODE, MEDADMIN_TYPE, OBSCLIN_CODE, OBSCLIN_TYPE, OBSGEN_CODE, OBSGEN_TYPE. Exceptions are highlighted in blue and should be investigated and explained in the ETL ADD.

		Records with missing, NI, UN, or OT values			
Table	Field	Numerator	Denominator	%	Source table
PROVIDER	PROVIDER_NPI	8,822	10,670	82.7	PROV_L3_N
PROVIDER	PROVIDER_NPI_FLAG	0	10,670		PROV_L3_NPIFLAG
PROVIDER	PROVIDER_SPECIALTY_PRIMARY	350	10,670	3.3	PROV_L3_SPECIALTY
PROVIDER	PROVIDER_SEX	1,307	10,670	12.2	PROV_L3_SEX
MED_ADMIN	ENCOUNTERID	728	4,349	**16.7**	MEDADM_L3_N
MED_ADMIN	PRESCRIBINGID	482	4,349	11.1	MEDADM_L3_N
MED_ADMIN	MEDADMIN_PROVIDERID	739	4,349	17.0	MEDADM_L3_N
MED_ADMIN	MEDADMIN_DOSE_ADMIN	46	4,349	1.1	MEDADM_L3_DOSEADM
MED_ADMIN	MEDADMIN_DOSE_ADMIN_UNIT	122	4,349	2.8	MEDADM_L3_DOSEADMUNIT
MED_ADMIN	MEDADMIN_ROUTE	259	4,349	6.0	MEDADM_L3_ROUTE
MED_ADMIN	MEDADMIN_SOURCE	563	4,349	**12.9**	MEDADM_L3_SOURCE
MED_ADMIN	MEDADMIN_CODE	940	4,349	**21.6**	MEDADM_L3_CODE_TYPE
MED_ADMIN	MEDADMIN_TYPE	568	4,349	**13.1**	MEDADM_L3_TYPE
OBS_CLIN	ENCOUNTERID	3,354	3,354	**100.0**	OBSCLIN_L3_N
OBS_CLIN	OBSCLIN_PROVIDERID	584	3,354	17.4	OBSCLIN_L3_N
OBS_CLIN	OBSCLIN_START_TIME	139	3,354	4.1	XTBL_L3_TIMES
OBS_CLIN	OBSCLIN_STOP_DATE	72	3,354	2.1	XTBL_L3_DATES
OBS_CLIN	OBSCLIN_STOP_TIME	122	3,354	3.6	XTBL_L3_TIMES

Sample data

Table excludes records with values outside of CDM specifications.
The four 'flavors of null' defined in the CDM are combined here but details are available in the source tables.
The denominator is derived from the applicable _N query (ALL_N + NULL_N for TAG=PATID).

Table IVD. Missing or Unknown Values, Optional Tables (continued - page 5 of 6)

This table includes most of the fields in the VITAL, DEATH, LAB_RESULT_CM, PRESCRIBING, DISPENSING, CONDITION, DEATH_CAUSE, PROVIDER, MED_ADMIN, OBS_CLIN and OBS_GEN tables where the field is included in the query results and is not required to be populated. Vital measures (e.g. HEIGHT) are not included because the table structure does not support missingness assessment. The table depicts the percentage of records with missing or unknown values. Results support Data Check 3.03 (more than 10% of records have missing or unknown values) for the following fields: RX_ORDER_DATE, VX_RECORD_DATE, DISPENSE_SUP, DEATH_SOURCE, DISPENSE_SOURCE, CONDITION_SOURCE, LAB_RESULT_SOURCE, MEDADMIN_SOURCE, RX_SOURCE, VITAL_SOURCE, VX_SOURCE, VITAL.ENCOUNTERID, MED_ADMIN.ENCOUNTERID, LAB_RESULT_CM.ENCOUNTERID, MEDADMIN_CODE, MEDADMIN_TYPE, OBSCLIN_CODE, OBSCLIN_TYPE, OBSGEN_CODE, OBSGEN_TYPE. Exceptions are highlighted in blue and should be investigated and explained in the ETL ADD.

		Records with missing, NI, UN, or OT values			
Table	Field	Numerator	Denominator	%	Source table
OBS_CLIN	OBSCLIN_CODE	762	3,354	**22.7**	OBSCLIN_L3_CODE_TYPE
OBS_CLIN	OBSCLIN_TYPE	762	3,354	**22.7**	OBSCLIN_L3_TYPE
OBS_CLIN	OBSCLIN_RESULT_QUAL	163	3,354	4.9	OBSCLIN_L3_QUAL
OBS_CLIN	OBSCLIN_RESULT_MODIFIER	115	3,354	3.4	OBSCLIN_L3_MOD
OBS_CLIN	OBSCLIN_RESULT_UNIT	2,347	3,354	70.0	OBSCLIN_L3_RUNIT
OBS_CLIN	OBSCLIN_ABN_IND	138	3,354	4.1	OBSCLIN_L3_ABN
IMMUNIZATION	ENCOUNTERID	1	166,567	0.0	IMMUNE_L3_N
IMMUNIZATION	PROCEDURESID	0	166,567		IMMUNE_L3_N
IMMUNIZATION	VX_PROVIDERID	1,628	166,567	1.0	IMMUNE_L3_N
IMMUNIZATION	VX_RECORD_DATE	3,577	166,567	2.1	XTBL_L3_DATES
IMMUNIZATION	VX_ADMIN_DATE	3,681	166,567	2.2	XTBL_L3_DATES
IMMUNIZATION	VX_CODE_TYPE	0	166,567		IMMUNE_L3_CODETYPE
IMMUNIZATION	VX_STATUS	4,078	166,567	2.4	IMMUNE_L3_STATUS
IMMUNIZATION	VX_STATUS_REASON	4,078	166,567	2.4	IMMUNE_L3_STATUSREASON
IMMUNIZATION	VX_SOURCE	6,734	166,567	4.0	IMMUNE_L3_SOURCE
IMMUNIZATION	VX_DOSE	83,325	166,567	50.0	IMMUNE_L3_DOSE_DIST
IMMUNIZATION	VX_DOSE_UNIT	5,438	166,567	3.3	IMMUNE_L3_DOSEUNIT
IMMUNIZATION	VX_ROUTE	9,510	166,567	5.7	IMMUNE_L3_ROUTE

Sample data

Table excludes records with values outside of CDM specifications.
The four 'flavors of null' defined in the CDM are combined here but details are available in the source tables.
The denominator is derived from the applicable _N query (ALL_N + NULL_N for TAG=PATID).

Table IVD. Missing or Unknown Values, Optional Tables (continued - page 6 of 6)

This table includes most of the fields in the VITAL, DEATH, LAB_RESULT_CM, PRESCRIBING, DISPENSING, CONDITION, DEATH_CAUSE, PROVIDER, MED_ADMIN, OBS_CLIN and OBS_GEN tables where the field is included in the query results and is not required to be populated. Vital measures (e.g. HEIGHT) are not included because the table structure does not support missingness assessment. The table depicts the percentage of records with missing or unknown values. Results support Data Check 3.03 (more than 10% of records have missing or unknown values) for the following fields: RX_ORDER_DATE, VX_RECORD_DATE, DISPENSE_SUP, DEATH_SOURCE, DISPENSE_SOURCE, CONDITION_SOURCE, LAB_RESULT_SOURCE, MEDADMIN_SOURCE, RX_SOURCE, VITAL_SOURCE, VX_SOURCE, VITAL.ENCOUNTERID, MED_ADMIN.ENCOUNTERID, LAB_RESULT_CM.ENCOUNTERID, MEDADMIN_CODE, MEDADMIN_TYPE, OBSCLIN_CODE, OBSCLIN_TYPE, OBSGEN_CODE, OBSGEN_TYPE. Exceptions are highlighted in blue and should be investigated and explained in the ETL ADD.

		Records with missing, NI, UN, or OT values			
Table	Field	Numerator	Denominator	%	Source table
IMMUNIZATION	VX_BODY_SITE	4,240	166,567	2.5	IMMUNE_L3_BODYSITE
IMMUNIZATION	VX_MANUFACTURER	12,219	166,567	7.3	IMMUNE_L3_MANUFACTURER
IMMUNIZATION	VX_LOT_NUM	0	166,567		IMMUNE_L3_LOTNUM
IMMUNIZATION	VX_EXP_DATE	3,337	166,567	2.0	XTBL_L3_DATES

Sample data

Table excludes records with values outside of CDM specifications.
The four 'flavors of null' defined in the CDM are combined here but details are available in the source tables.
The denominator is derived from the applicable _N query (ALL_N + NULL_N for TAG=PATID).

Table IVE. Principal Diagnoses for Institutional Encounters

This table shows principal diagnosis code data availability for institutional encounters. Results support Data Check 3.06 (More than 10% of IP (inpatient) or ED to inpatient (EI) encounters with any diagnosis from a known DX_ORIGIN don't have a principal diagnosis from that source) and Data Check 2.07 (the average number of principal diagnoses per known DX_ORIGIN per encounter is above threshold [2.0 for inpatient (IP) and ED to inpatient (EI]). For data check 3.06, exceptions are triggered when the percentage exceeds 10% or when 0 records have a principal diagnosis. Exceptions are highlighted in blue and should be investigated and explained in the ETL ADD.

Encounter Type	DX_ORIGIN	Distinct encounter IDs with a principal diagnosis	Distinct encounter IDs without a principal diagnosis	% of encounters without a principal diagnosis	Principal diagnoses	Principal diagnoses per encounter with any principal diagnosis	Source table
EI (ED to IP Stay)	BI (billing)	695	0	0.0	695	1.0	DIA_L3_PDX_ENCTYPE
	CL (claim)	684	0	0.0	684	1.0	DIA_L3_PDXGRP_ENCTYPE
	DR (derived)	16	667	**97.7**	16	1.0	
	OD (Order/EHR)	19	666	**97.2**	19	1.0	
IP (Inpatient Hospital Stay)	BI (billing)	665	0	0.0	665	1.0	DIA_L3_PDX_ENCTYPE
	CL (claim)	709	0	0.0	709	1.0	DIA_L3_PDXGRP_ENCTYPE
	DR (derived)	17	677	**97.6**	17	1.0	
	OD (Order/EHR)	14	600	**97.7**	14	1.0	
IS (Non-acute Institutional Stay)	BI (billing)	694	0	0.0	694	1.0	DIA_L3_PDX_ENCTYPE
	CL (claim)	669	0	0.0	669	1.0	DIA_L3_PDXGRP_ENCTYPE
	DR (derived)	19	710	97.4	19	1.0	
	OD (Order/EHR)	25	713	96.6	25	1.0	
OS (Observation Stay)	BI (billing)	695	0	0.0	695	1.0	DIA_L3_PDX_ENCTYPE
	CL (claim)	691	0	0.0	691	1.0	DIA_L3_PDXGRP_ENCTYPE
	DR (derived)	20	619	96.9	20	1.0	
	OD (Order/EHR)	14	679	98.0	14	1.0	

Sample data

Table excludes records with values outside of CDM specifications.
Percentage is calculated by dividing the number of encounters (i.e. distinct ENCOUNTERIDs) without a principal diagnosis by the total number of encounters in the DIAGNOSIS table.
The number of encounters without a principal diagnosis is obtained by identifying distinct encounterids which are not in a list of distinct encounterIDs where PDX=P.
Number of principal diagnoses per encounter with any principal diagnosis is calculated by dividing the number of principal diagnoses by the number of encounters with a principal diagnosis.

Table IVF. Data Latency and Completeness of Encounter, Diagnoses, and Procedures, Past 2 Years

This table includes ENCOUNTER, DIAGNOSIS, and PROCEDURES from the most recent 24 month period; month -0 is the month the data curation query was run. Data completeness is determined by comparing the actual volume to the expected volume in each month. Expected volume is determined by taking the average volume during the benchmark period of months -12 to month -23. Data completeness is reported as a percentage of the benchmark average. Temporal differences may be affected by data availability, ETL processes, date shifting, secular trends, and/or changes in data provenance.

These data support Data Check 3.07 (encounters, diagnoses, or procedures in an ambulatory (AV), telehealth (TH), emergency department (ED), ED to inpatient (EI), or inpatient (IP) setting are less than 75% complete two months prior to the current month). Data check exceptions occur if the month -2 result is <75% of the benchmark average or 0 records. Data check exceptions are highlighted in blue. Data check exceptions and unexpected results (e.g. significant discrepancies in data completeness between the tables) should be investigated and explained in the ETL ADD.

		Ambulatory, Teleheath, ED, Inpatient or ED-to-Inpatient encounters		Ambulatory, Teleheath, ED, Inpatient or ED-to-Inpatient diagnoses		Ambulatory, Teleheath, ED, Inpatient or ED-to-Inpatient procedures	
Month	Calendar Month	Records	Percent of benchmark average	Records	Percent of benchmark average	Records	Percent of benchmark average
Month -0	01/2025	0		0		0	
Month -1	12/2024	641	78.8	182	76.5	933	77.1
Month -2	11/2024	619	76.1	186	78.2	897	**74.1**
Month -3	10/2024	686	84.4	194	81.5	979	80.9
Month -4	09/2024	673	82.8	168	70.6	999	82.6
Month -5	08/2024	672	82.7	0		978	80.8
Month -6	07/2024	636	78.2	0		1,004	83.0
Month -7	06/2024	671	82.5	184	77.3	908	75.0
Month -8	05/2024	655	80.6	200	84.0	981	81.1
Month -9	04/2024	616	75.8	187	78.6	947	78.3
Month -10	03/2024	680	83.6	163	68.5	961	79.4
Month -11	02/2024	591	72.7	194	81.5	895	74.0

Sample data

Table IVF. Data Latency and Completeness of Encounter, Diagnoses, and Procedures, Past 2 Years (continued)

This table includes ENCOUNTER, DIAGNOSIS, and PROCEDURES from the most recent 24 month period; month -0 is the month the data curation query was run. Data completeness is determined by comparing the actual volume to the expected volume in each month. Expected volume is determined by taking the average volume during the benchmark period of months -12 to month -23. Data completeness is reported as a percentage of the benchmark average. Temporal differences may be affected by data availability, ETL processes, date shifting, secular trends, and/or changes in data provenance.

These data support Data Check 3.07 (encounters, diagnoses, or procedures in an ambulatory (AV), telehealth (TH), emergency department (ED), ED to inpatient (EI), or inpatient (IP) setting are less than 75% complete two months prior to the current month). Data check exceptions occur if the month -2 result is <75% of the benchmark average or 0 records. Data check exceptions are highlighted in blue. Data check exceptions and unexpected results (e.g. significant discrepancies in data completeness between the tables) should be investigated and explained in the ETL ADD.

		Ambulatory, ED, Inpatient or ED-to-Inpatient encounters		Ambulatory, ED, Inpatient or ED-to-Inpatient diagnoses		Ambulatory, ED, Inpatient or ED-to-Inpatient procedures	
Month	Calendar Month	Records	Percent of benchmark average	Records	Percent of benchmark average	Records	Percent of benchmark average
Benchmark Period							
Month -12	01/2024	638	78.5	188	79.0	983	81.2
Month -13	12/2023	652	80.2	213	89.5	972	80.3
Month -14	11/2023	647	79.6	185	77.7	1,005	83.1
Month -15	10/2023	640	78.7	176	73.9	1,024	84.6
Month -16	09/2023	649	79.8	189	79.4	900	74.4
Month -17	08/2023	678	83.4	193	81.1	980	81.0
Month -18	07/2023	673	82.8	193	81.1	1,014	83.8
Month -19	06/2023	620	76.3	211	88.7	890	73.6
Month -20	05/2023	687	84.5	187	78.6	979	80.9
Month -21	04/2023	1,036	127.4	263	110.5	1,503	124.2
Month -22	03/2023	1,508	185.5	442	185.7	2,204	182.1
Month -23	02/2023	1,328	163.3	415	174.4	2,060	170.2
Benchmark average		813		238		1,210	
Source table		ENC_L3_ ENCTYPE_ ADATE_YM		DIA_L3_ ENCTYPE_ ADATE_YM		PRO_L3_ ENCTTPE_ ADATE_YM	

Sample data

Table IVG. Data Latency and Completeness of Vital, Prescribing, and Lab Data, Past 2 Years

This table includes VITAL, PRESCRIBING, and LAB_RESULT_CM data from the most recent 24 month period; month -0 is the month the data curation query was run. Data completeness is determined by comparing the actual volume to the expected volume in each month. Expected volume is determined by taking the average volume during the benchmark period of months -12 to month -23. Data completeness is reported as a percentage of the benchmark average. Temporal differences may be affected by data availability, ETL processes, date shifting, secular trends, and/or changes in data provenance.

These data support Data Check 3.11 (vital, prescribing, or laboratory records are less than 75% complete three months prior to the current month). Data check exceptions occur if the month -3 result is <75% of the benchmark average or 0 records. Data check exceptions are highlighted in blue. Data check exceptions and unexpected results should be investigated and explained in the ETL ADD.

		Vitals		Prescriptions		Labs	
Month	Calendar Month	Records	Percent of benchmark average	Records	Percent of benchmark average	Records	Percent of benchmark average
Month -0	01/2025	0		0		0	
Month -1	12/2024	52	89.7	78	68.4	127	83.0
Month -2	11/2024	44	75.9	97	85.1	138	90.2
Month -3	10/2024	33	**56.9**	88	77.2	141	92.2
Month -4	09/2024	44	75.9	77	67.5	128	83.7
Month -5	08/2024	31	53.4	88	77.2	130	85.0
Month -6	07/2024	47	81.0	93	81.6	123	80.4
Month -7	06/2024	36	62.1	93	81.6	124	81.0
Month -8	05/2024	49	84.5	78	68.4	119	77.8
Month -9	04/2024	41	70.7	91	79.8	128	83.7
Month -10	03/2024	45	77.6	78	68.4	144	94.1
Month -11	02/2024	40	69.0	82	71.9	103	67.3

Sample data

Table IVG. Data Latency and Completeness of Vital, Prescribing, and Lab Data, Past 2 Years (continued)

This table includes VITAL, PRESCRIBING, and LAB_RESULT_CM data from the most recent 24 month period; month -0 is the month the data curation query was run. Data completeness is determined by comparing the actual volume to the expected volume in each month. Expected volume is determined by taking the average volume during the benchmark period of months -12 to month -23. Data completeness is reported as a percentage of the benchmark average. Temporal differences may be affected by data availability, ETL processes, date shifting, secular trends, and/or changes in data provenance.

These data support Data Check 3.11 (vital, prescribing, or laboratory records are less than 75% complete three months prior to the current month). Data check exceptions occur if the month -3 result is <75% of the benchmark average or 0 records. Data check exceptions are highlighted in blue. Data check exceptions and unexpected results should be investigated and explained in the ETL ADD.

		Vitals		Prescriptions		Labs	
Month	Calendar Month	Records	Percent of benchmark average	Records	Percent of benchmark average	Records	Percent of benchmark average
Benchmark Period							
Month -12	01/2024	47	81.0	101	88.6	128	83.7
Month -13	12/2023	38	65.5	93	81.6	146	95.4
Month -14	11/2023	48	82.8	92	80.7	109	71.2
Month -15	10/2023	34	58.6	104	91.2	120	78.4
Month -16	09/2023	48	82.8	83	72.8	128	83.7
Month -17	08/2023	62	106.9	100	87.7	129	84.3
Month -18	07/2023	48	82.8	90	78.9	122	79.7
Month -19	06/2023	50	86.2	105	92.1	120	78.4
Month -20	05/2023	44	75.9	95	83.3	132	86.3
Month -21	04/2023	58	100.0	145	127.2	168	109.8
Month -22	03/2023	109	187.9	193	169.3	285	186.3
Month -23	02/2023	112	193.1	166	145.6	250	163.4
Benchmark average		58		114		153	
Source table		VIT_L3_ MDATE_YM		PRES_L3_ ODATE_YM		LAB_L3_ RDATE_YM	

Sample data

Table IVH. RXNORM Term Type Mapping

This table shows the number of records in the PRESCRIBING and MED_ADMIN tables by RXNORM Term Type tier. Guidance on mapping prescribing orders to RXNORM is provided in the CDM specifications. These data support Data Check 3.08 (less than 80% of prescribing orders are mapped to a RXNORM_CUI which fully specifies the ingredient, strength and dose form) and Data Check 3.15 (less than 80% of medication administrations mapped to RXNORM are mapped to a RXNORM_CUI that fully specifies the ingredient, strength and dose form). The numerator is the number of records per RXNORM tier; the denominator is the total number of records in the table for the specified time period. Data check results are shown for all years and for the most recent 5 years. Data check exceptions occur if the Tier 1 percentage is <80% or the numerator is 0. Data check exceptions are highlighted in blue and should be investigated and explained in the ETL ADD. Data check exceptions for the most recent 5 year period for prescribing orders will be used to assess compliance with the terms of the CRN Scope of Work. The percentage of prescribing orders mapped to Tier 1 brand drugs (a subset of the Tier 1 percentage) is also displayed.

Table	Term Type Tier	Term Type Tier Description	Term Types	Numerator	Percentage	Source table
PRESCRIBING	Tier 1	RXNORM_CUI encodes ingredient(s), strength and dose form	SCD, SBD, BPCK, and GPCK	1,088	**14.36**	PRES_L3_ RXCUI_TIER
	Tier 1 brand	RXNORM_CUI encodes ingredient(s), strength, dose form and brand	SBD, BPCK	269	24.72	PRES_L3_ RXCUI
	Tier 2	RXNORM_CUI encodes ingredient(s) and potentially strength or dose form. Can still represent medications with multiple ingredients with a single RXCUI.	SBDF, SBDFP, SCDF, SCDFP, SBDG, SCDG, SCDGP, SBDC, BN, and MIN	3,383	44.67	PRES_L3_ RXCUI_TIER
	Tier 3	Requires more than one RXNORM_CUI to represent medications with multiple ingredients.	SCDC, PIN, and IN	2,785	36.77	PRES_L3_ RXCUI_TIER
	Tier 4	RXNORM_CUI does not encode any ingredient information.	DF and DFG	0	0.00	PRES_L3_ RXCUI_TIER
	Unknown	RXNORM_CUI was not populated or could not be matched to the reference table	n/a	318	4.20	PRES_L3_ RXCUI_TIER

Sample data

Term types were obtained from the rxnorm_cui_ref table. This table was created from the RxNorm 10/07/2024 Full Update Release. It includes all RXCUIs from the RXNORM terminology (i.e., SAB=RXNORM) with one of the following term types: BN (Brand Name), BPCK (Brand Name Pack), DF (Dose Form), DFG (Dose Form Group), GPCK (Generic Pack), IN (Ingredient), MIN (Multiple Ingredients), SBD (Semantic Branded Drug), SBDC (Semantic Branded Drug Component), SBDF (Semantic Branded Drug Form), SBDFP (Semantic Branded Drug Form Precise), SBDG (Semantic Branded Dose Form Group), SCD (Semantic Clinical Drug), SCDC (Semantic Clinical Drug Component), SCDF (Semantic Clinical Drug Form), SCDFP (Semantic Clinical Drug Form Precise), SCDG (Semantic Clinical Dose Form Group), SCDGP (Semantic Clinical Dose Form Group Precise), or PIN (Precise Ingredient).

Table IVH. RXNORM Term Type Mapping (continued)

This table shows the number of records in the PRESCRIBING and MED_ADMIN tables by RXNORM Term Type tier. Guidance on mapping prescribing orders to RXNORM is provided in the CDM specifications. These data support Data Check 3.08 (less than 80% of prescribing orders are mapped to a RXNORM_CUI which fully specifies the ingredient, strength and dose form) and Data Check 3.15 (less than 80% of medication administrations mapped to RXNORM are mapped to a RXNORM_CUI that fully specifies the ingredient, strength and dose form). The numerator is the number of records per RXNORM tier; the denominator is the total number of records in the table for the specified time period. Data check results are shown for all years and for the most recent 5 years. Data check exceptions occur if the Tier 1 percentage is <80% or the numerator is 0. Data check exceptions are highlighted in blue and should be investigated and explained in the ETL ADD. Data check exceptions for the most recent 5 year period for prescribing orders will be used to assess compliance with the terms of the CRN Scope of Work. The percentage of prescribing orders mapped to Tier 1 brand drugs (a subset of the Tier 1 percentage) is also displayed.

Table	Term Type Tier	Term Type Tier Description	Term Types	Numerator	Percentage	Source table
MED_ADMIN	Tier 1	MEDADMIN_CODE encodes ingredient(s), strength and dose form	SCD, SBD, BPCK, and GPCK	217	**13.40**	MEDADM_ L3_RXCUI_ TIER
	Tier 2	MEDADMIN_CODE encodes ingredient(s) and potentially strength or dose form. Can still represent medications with multiple ingredients with a single RXCUI.	SBDF, SBDFP, SCDF, SCDFP, SBDG, SCDG, SCDGP, SBDC, BN, and MIN	755	46.80	MEDADM_ L3_RXCUI_ TIER
	Tier 3	Requires more than one MEDADMIN_CODE to represent medications with multiple ingredients.	SCDC, PIN, and IN	614	38.00	MEDADM_ L3_RXCUI_ TIER
	Tier 4	MEDADMIN_CODE does not encode any ingredient information.	DF and DFG	0	0.00	MEDADM_ L3_RXCUI_ TIER
	Unknown	MEDADMIN_CODE was not populated or could not be matched to the reference table	n/a	28	1.70	MEDADM_ L3_RXCUI_ TIER

Sample data

Term types were obtained from the rxnorm_cui_ref table. This table was created from the RxNorm 10/07/2024 Full Update Release. It includes all RXCUIs from the RXNORM terminology (i.e., SAB=RXNORM) with one of the following term types: BN (Brand Name), BPCK (Brand Name Pack), DF (Dose Form), DFG (Dose Form Group), GPCK (Generic Pack), IN (Ingredient), MIN (Multiple Ingredients), SBD (Semantic Branded Drug), SBDC (Semantic Branded Drug Component), SBDF (Semantic Branded Drug Form), SBDFP (Semantic Branded Drug Form Precise), SBDG (Semantic Branded Dose Form Group), SCD (Semantic Clinical Drug), SCDC (Semantic Clinical Drug Component), SCDF (Semantic Clinical Drug Form), SCDFP (Semantic Clinical Drug Form Precise), SCDG (Semantic Clinical Dose Form Group), SCDGP (Semantic Clinical Dose Form Group Precise), or PIN (Precise Ingredient).

Table IVI. Laboratory and Clinical Observation Result Data Completeness

This table shows the level of data completeness for LAB_RESULT_CM and OBS_CLIN records and supports Data Check 3.09 (less than 80% of laboratory results are mapped to LAB_LOINC), Data Check 3.10 (less than 80% of quantitative results for tests mapped to LAB_LOINC fully specify the normal range), Data Check 3.12 (less than 80% of quantitative results for tests mapped to LAB_LOINC fully specify the result unit), Data Check 3.16 (less than 80% of clinical observations are mapped to an OBSCLIN_CODE and have a quantitative, qualitative, or narrative result), and Data Check 3.17 (less than 80% of quantitative results for tests mapped to OBSCLIN_CODE fully specify the RESULT_UNIT). Data check exceptions occur if the percentages are <80% or the numerator is 0. Data check results are shown for all years and for the most recent 5 years. Data check exceptions are highlighted in blue and should be investigated and explained in the ETL ADD. Data check exceptions for the most recent 5 year period will be used to assess compliance with the terms of the CRN Scope of Work.

Table	Data Check	Description	Numerator	Denominator	Percentage	Source table
LAB_RESULT_CM	n/a	Number of distinct LAB_LOINCs	9			LAB_L3_LOINC
	n/a	Results mapped to a known LAB_LOINC	10,508	10,508	100.00	LAB_L3_RECORDC; LAB_L3_N
	3.09	Results mapped to a known LAB_LOINC with a known result	10,468	10,508	99.62	LAB_L3_RECORDC
	n/a	Quantitative results	6,368			LAB_L3_RECORDC
	3.10	Quantitative results which fully specify the normal range	**0**	6,368	**0.00**	
	3.12	Quantitative results which specify the result unit	**0**	6,368	**0.00**	
OBS_CLIN	n/a	Number of distinct OBSCLIN_CODEs	1,595			OBSCLIN_L3_CODE_TYPE
	n/a	Results mapped to a known OBSCLIN_CODE	2,592	3,354	77.28	OBSCLIN_L3_RECORDC; OBSCLIN_L3_N
	3.16	Results mapped to a known OBSCLIN_CODE with a known result	2,558	3,354	**76.27**	OBSCLIN_L3_RECORDC; OBSCLIN_L3_N
	n/a	Quantitative results	1,837			OBSCLIN_L3_RECORDC
	3.17	Quantitative results which specify the result unit	**0**	1,837	**0.00**	OBSCLIN_L3_RECORDC

Sample data

The denominator for the percentage of results mapped to a known LAB_LOINC or OBSCLIN_CODE is the total number of records in the table (ALL_N + NULL_N for the LAB_RESULT_CM_ID or OBSCLIN_ID field). The criteria for each measurement are described in table IVI_REF.

Table IVI_Ref. Laboratory and Clinical Observation Result Data Completeness Definitions

This table shows the definitions for each of the numerator values in Table IVI.

Description	Source Table Row	CODE criteria	RESULT_ NUM criteria	RESULT_MODIFIER criteria	Other criteria
Number of distinct codes		Not null			
Results mapped to a known code	KNOWN_TEST	Not null			
Results mapped to a known code with a known result	KNOWN_TEST_ RESULT	Not null			[RESULT_NUM is not null and RESULT_MODIFIER is not in (NI, UN, OT, null)] or RESULT_QUAL is not in (NI, UN, OT, null) or RESULT_TEXT is not null
Quantitative results	KNOWN_TEST_ RESULT_NUM	Not null	Not null	not in (NI, UN, OT, null)	
Quantitative results which specify the specimen source	KNOWN_TEST_ RESULT_NUM_ SOURCE	Not null	Not null	not in (NI, UN, OT, null)	SPECIMEN_SOURCE is not in ('NI','UN','OT', 'UNK_SUB', 'SMPLS', 'SPECIMEN', null).
Quantitative results which specify the result unit	KNOWN_TEST_ RESULT_NUM_ UNIT	Not null	Not null	not in (NI, UN, OT, null)	RESULT_UNIT is not in ('NI','UN','OT', null)
Quantitative results which fully specify the normal range.	KNOWN_TEST_ NUM_RESULT_ RANGE	Not null	Not null	not in (NI, UN, OT, null)	[NORM_MODIFIER_LOW='EQ' and NORM_MODIFIER_HIGH='EQ' and NORM_RANGE_LOW is not null and NORM_RANGE_HIGH is not null] or [NORM_MODIFIER_LOW in ('GT','GE') and NORM_MODIFER_HIGH='NO' and NORM_RANGE_LOW is not null and NORM_RANGE_HIGH is null] or [NORM_MODIFIER_HIGH in ('LE','LT') and NORM_MODIFIER_LOW='NO' and NORM_RANGE_HIGH is not null and NORM_RANGE_LOW is null].

Sample data

Table IVJ. Data Latency and Completeness of Medication Administration, Dispensing and Clinical Observation Data, Past 2 Years

This table includes MED_ADMIN, DISPENSING, and OBS_CLIN data from the most recent 24 month period; month -0 is the month the data curation query was run. Data completeness is determined by comparing the actual volume to the expected volume in each month. Expected volume is determined by taking the average volume during the benchmark period of months -12 to month -23. Data completeness is reported as a percentage of the benchmark average. Temporal differences may be affected by data availability, ETL processes, date shifting, secular trends, and/or changes in data provenance.

These data support Data Check 3.14 (medication administration, dispensing, or clinical observation records are less than 75% complete three months prior to the current month). Data check exceptions occur if the month -3 result is <75% of the benchmark average or 0 records. Data check exceptions are highlighted in blue. Data check exceptions and unexpected results should be investigated and explained in the ETL ADD.

		MED_ADMIN		DISPENSING		OBS_CLIN	
Month	Calendar Month	Records	Percent of benchmark average	Records	Percent of benchmark average	Records	Percent of benchmark average
Month -0	01/2025	0		0		0	
Month -1	12/2024	61	95.3	0		45	90.0
Month -2	11/2024	54	84.4	0		39	78.0
Month -3	10/2024	50	78.1	**0**		45	90.0
Month -4	09/2024	66	103.1	0		27	54.0
Month -5	08/2024	56	87.5	0		37	74.0
Month -6	07/2024	54	84.4	0		47	94.0
Month -7	06/2024	67	104.7	174	86.6	42	84.0
Month -8	05/2024	51	79.7	183	91.0	37	74.0
Month -9	04/2024	63	98.4	157	78.1	38	76.0
Month -10	03/2024	56	87.5	155	77.1	49	98.0
Month -11	02/2024	64	100.0	170	84.6	34	68.0

Sample data

Table IVJ. Data Latency and Completeness of Medication Administration, Dispensing and Clinical Observation Data, Past 2 Years (continued)

This table includes MED_ADMIN, DISPENSING, and OBS_CLIN data from the most recent 24 month period; month -0 is the month the data curation query was run. Data completeness is determined by comparing the actual volume to the expected volume in each month. Expected volume is determined by taking the average volume during the benchmark period of months -12 to month -23. Data completeness is reported as a percentage of the benchmark average. Temporal differences may be affected by data availability, ETL processes, date shifting, secular trends, and/or changes in data provenance.

These data support Data Check 3.14 (medication administration, dispensing, or clinical observation records are less than 75% complete three months prior to the current month). Data check exceptions occur if the month -3 result is <75% of the benchmark average or 0 records. Data check exceptions are highlighted in blue. Data check exceptions and unexpected results should be investigated and explained in the ETL ADD.

		MED_ADMIN		DISPENSING		OBS_CLIN	
Month	Calendar Month	Records	Percent of benchmark average	Records	Percent of benchmark average	Records	Percent of benchmark average
Benchmark Period							
Month -12	01/2024	52	81.3	149	74.1	45	90.0
Month -13	12/2023	49	76.6	178	88.6	37	74.0
Month -14	11/2023	62	96.9	158	78.6	46	92.0
Month -15	10/2023	63	98.4	147	73.1	37	74.0
Month -16	09/2023	47	73.4	165	82.1	38	76.0
Month -17	08/2023	47	73.4	163	81.1	48	96.0
Month -18	07/2023	61	95.3	158	78.6	45	90.0
Month -19	06/2023	45	70.3	145	72.1	36	72.0
Month -20	05/2023	56	87.5	160	79.6	38	76.0
Month -21	04/2023	76	118.8	249	123.9	55	110.0
Month -22	03/2023	106	165.6	383	190.5	92	184.0
Month -23	02/2023	108	168.8	357	177.6	85	170.0
Benchmark average		64		201		50	
Source table		MEDADM_L3_ SDATE_YM		DISP_L3_ DDATE_YM		OBSCLIN_L3_ SDATE_YM	

Sample data

Table VA. Changes in Tables

This table shows changes in key DataMart attributes between the most recent approved DataMart refresh and the current DataMart refresh and supports Data Check 4.01 (more than a 5% decrease in the number of patients or records in a CDM table). Data check exceptions are highlighted in blue and should be investigated and explained in the ETL ADD.

Table	Records			Patients			Source table
	Previous Refresh	Current Refresh	Percent Change	Previous Refresh	Current Refresh	Percent Change	
DEMOGRAPHIC	3,644	3,644	0.0	3,644	3,644	0.0	DEM_L3_N
ENROLLMENT	0	0		0	0		ENR_L3_N
ENCOUNTER	107,044	107,044	0.0	4,039	4,039	0.0	ENC_L3_N
DIAGNOSIS	33,537	32,031	-4.5	3,176	3,138	-1.2	DIA_L3_N
PROCEDURES	168,793	186,105	10.3	3,988	4,005	0.4	PRO_L3_N
VITAL	3,765	3,765	0.0	2,299	2,299	0.0	VIT_L3_N
DEATH	1,216	1,216	0.0	1,216	1,216	0.0	DEATH_L3_N
PRESCRIBING	9,573	7,574	**-20.9**	3,365	3,365	0.0	PRES_L3_N
DISPENSING	12,323	12,323	0.0	4,557	4,557	0.0	DISP_L3_N
LAB_RESULT_CM	10,508	10,508	0.0	4,330	4,330	0.0	LAB_L3_N
CONDITION	0	0		0	0		COND_L3_N
DEATH_CAUSE	0	0		0	0		DEATHC_L3_N
PRO_CM	0	0		0	0		PROCM_L3_N
PROVIDER	10,670	10,670	0.0	---	0		PROV_L3_N
MED_ADMIN	5,848	4,349	**-25.6**	4,076	4,076	0.0	MEDADM_L3_N
OBS_CLIN	3,354	3,354	0.0	3,192	3,192	0.0	OBSCLIN_L3_N
OBS_GEN	0	0		0	0		OBSGEN_L3_N
HASH_TOKEN	5,720	5,720	0.0	4,720	4,720	0.0	HASH_L3_N
IMMUNIZATION	166,567	166,567	0.0	3,967	3,967	0.0	IMMUNE_L3_N
LDS_ADDRESS_ HISTORY	0	0		0	0		LDSADRS_L3_N

Percent change is calculated as current minus previous divided by previous multipled by 100.

Sample data

Table VB. Changes in Selected Encounter Types and Domains

This table shows changes in key DataMart attributes between the most recent approved DataMart refresh and the current DataMart refresh and supports Data Check 4.02 [more than a 5% decrease in the number of patients or records for diagnosis, procedures, labs or prescriptions during an ambulatory (AV), telehealth (TH), other ambulatory (OA), emergency department (ED), or inpatient (IP) encounter]. Data check exceptions are highlighted in blue and should be investigated and explained in the ETL ADD.

	Records			Patients			
Encounter Type	Previous Refresh	Current Refresh	Percent Change	Previous Refresh	Current Refresh	Percent Change	Source table
Ambulatory Visit (AV)							
Diagnoses	3,213	3,077	-4.2	1,340	1,317	-1.7	DIA_L3_ENCTYPE
Procedures	16,425	18,114	10.3	2,851	2,914	2.2	PRO_L3_ENCTYPE
Labs	0	0		0	0		XTBL_L3_LAB_ENCTYPE
Prescriptions	456	361	**-20.8**	318	318	0.0	XTBL_L3_PRES_ENCTYPE
Emergency Department (ED)							
Diagnoses	3,302	3,146	-4.7	1,435	1,397	-2.6	DIA_L3_ENCTYPE
Procedures	16,432	18,156	10.5	2,844	2,915	2.5	PRO_L3_ENCTYPE
Labs	0	0		0	0		XTBL_L3_LAB_ENCTYPE
Prescriptions	433	328	**-24.2**	284	284	0.0	XTBL_L3_PRES_ENCTYPE
Inpatient (IP)							
Diagnoses	3,203	3,041	**-5.1**	1,352	1,308	-3.3	DIA_L3_ENCTYPE
Procedures	16,420	18,099	10.2	2,870	2,959	3.1	PRO_L3_ENCTYPE
Labs	0	0		0	0		XTBL_L3_LAB_ENCTYPE
Prescriptions	424	325	**-23.3**	284	284	0.0	XTBL_L3_PRES_ENCTYPE
Other Ambulatory (OA)							
Diagnoses	3,199	3,066	-4.2	1,351	1,325	-1.9	DIA_L3_ENCTYPE
Procedures	16,438	18,124	10.3	2,816	2,897	2.9	PRO_L3_ENCTYPE
Labs	0	0		0	0		XTBL_L3_LAB_ENCTYPE
Prescriptions	394	308	**-21.8**	271	271	0.0	XTBL_L3_PRES_ENCTYPE
Telehealth (TH) encounters							
Diagnoses	3,327	3,181	-4.4	1,391	1,355	-2.6	DIA_L3_ENCTYPE
Procedures	16,419	18,094	10.2	2,865	2,936	2.5	PRO_L3_ENCTYPE
Labs	0	0		0	0		XTBL_L3_LAB_ENCTYPE
Prescriptions	412	321	**-22.1**	295	295	0.0	XTBL_L3_PRES_ENCTYPE

Sample data

Percent change is calculated as current minus previous divided by previous multipled by 100.

Table VC. Changes in Selected Code Types

This table shows changes in key DataMart attributes between the most recent approved DataMart refresh and the current DataMart refresh and supports Data Check 4.03 (more than a 5% decrease in the number of records or distinct codes for CPT/HCPCS, CVX, ICD10, NDC, or RXCUI codes). The data check is not applied to ICD9 codes because these codes will decrease between refreshes because of the 10 year lookback. Data check exceptions are highlighted in blue and should be investigated and explained in the ETL ADD.

	Records			Distinct Codes			
	Previous Refresh	Current Refresh	Percent Change	Previous Refresh	Current Refresh	Percent Change	Source table
DIAGNOSIS							
09	28,799	28,057	-2.6	3,582	3,541	-1.1	DIA_L3_DX_DXTYPE
10	669	**0**	**-100.0**	415	**0**	**-100.0**	DIA_L3_DX_DXTYPE
PROCEDURES							
09	18,470	18,470	0.0	2,025	2,025	0.0	PRO_L3_PX_PXTYPE
10	19,375	19,375	0.0	1,651	1,651	0.0	PRO_L3_PX_PXTYPE
CH	---	17,312		---	1,620		PRO_L3_PX_PXTYPE
ND	19,069	19,069	0.0	1,676	1,676	0.0	PRO_L3_PX_PXTYPE
DISPENSING							
ND	12,323	12,323	0.0	12,323	12,323	0.0	DISP_L3_NDC
IMMUNIZATION							
CH	41,523	41,523	0.0	10	10	0.0	IMMUNE_L3_CODE_CODETYPE
CX	41,791	41,791	0.0	10	10	0.0	IMMUNE_L3_CODE_CODETYPE
ND	41,747	41,747	0.0	10	10	0.0	IMMUNE_L3_CODE_CODETYPE
RX	41,506	41,506	0.0	10	10	0.0	IMMUNE_L3_CODE_CODETYPE
MED_ADMIN							
ND	2,444	1,795	**-26.6**	1,795	1,795	0.0	MEDADM_L3_CODE_TYPE
RX	2,156	1,614	**-25.1**	50	50	0.0	MEDADM_L3_CODE_TYPE
PRESCRIBING							
RX	9,364	7,409	**-20.9**	50	50	0.0	PRES_L3_RXCUI

Percent change is calculated as current minus previous divided by previous multipled by 100.

Sample data
