# Supplementary material for: Assessing and improving research readiness in PCORnet®
Source: J Clin Transl Sci. 2025 Dec 17;9(1):e279. doi: 10.1017/cts.2025.10207 (PMC12722095; doi:10.1017/cts.2025.10207)
Supplement: Marsolo et al. supplementary material 2 — Marsolo et al. supplementary material [file S2059866125102070sup002.docx]

**Supplementary Material**

**Title:**

Assessing and Improving Research Readiness in PCORnet®

**Journal**

Journal of Clinical and Translational Science

**Authors:**

Keith Marsolo, PhD^1,2^; Laura Goettinger Qualls, MS^1^; Darcy Louzao, PhD^1^; Thomas A Phillips, MA^1^; Adrian F Hernandez, MD, MHS^1,3^; Lesley H Curtis, PhD^1,2^

^1^ Duke Clinical Research Institute, Duke University School of Medicine, Durham, NC, USA

^2^ Department of Population Health Sciences, Duke University School of Medicine, Durham, NC, USA

^3^ Department of Medicine, Duke University School of Medicine, Durham, NC, USA

**Corresponding Author:**

Keith Marsolo

300 W Morgan St, Suite 636, Durham, NC 27701

E-mail: keith.marsolo@duke.edu

Work: +1 919 668 8633

**Table Summaries:**

The following table provides a general overview of the different tables in the PCORnet® Common Data Model. The name of each table is provided in the Table Name column. A summary of the information stored in each table is provided in the “Domain Description” column. Any table-level implementation guidance (i.e., guidance that applies to many fields and/or records in each table) is shown in the “Table Implementation Guidance” column. This version of the table is reflective of the specifications of PCORnet® CDM version 7.0. Please see the full PCORnet® CDM specifications for more information (<https://pcornet.org/data/common-data-model/>).

| **Table Name** | **Domain Description** | **Table Implementation Guidance** |
| --- | --- | --- |
| DEMOGRAPHIC | Demographics record the direct attributes of individual patients | - The most recently available information should be populated for BIRTH_DATE, SEX, and other characteristics. If these attributes have been updated in the patient record, use the most recent value. |
| ENROLLMENT | Enrollment is a concept that defines a period of time during which a person is expected to have complete data capture. This concept is often insurance-based, but other methods of defining enrollment are possible. | - For partners that do not have insurance-based enrollment information for some of their patients, other approaches can be used to identify periods during which complete medical capture is expected. - Members with medical insurance coverage, with or without drug coverage, or should be included. If a patient has both medical and drug coverage, create the appropriate enrollment records for each. - A break in insurance coverage of at least one day or a change in the chart abstraction flag should generate a new record. - The ENROLLMENT table provides an important analytic basis for identifying periods during which medical care should be observed, for calculating person-time, and for inferring the meaning of unobserved care (i.e., if care is not observed, it likely did not happen). The most recently available information should be populated for BIRTH_DATE, SEX, and other characteristics. If these attributes have been updated in the patient record, please use the most recent value. |
| ENCOUNTER | Encounters are interactions between patients and providers within the context of healthcare delivery. | - Each ENCOUNTERID will generally reflect a unique combination of PATID, ADMIT_DATE, PROVIDERID and ENC_TYPE. - Every diagnosis and procedure recorded during the encounter should have a separate record in the DIAGNOSIS or PROCEDURES Tables. - Multiple visits to the **same** provider on the same day may be considered one encounter, especially if defined by a reimbursement basis; if so, the ENCOUNTER record should be associated with all diagnoses and procedures that were recorded during those visits. - Visits to **different** providers for different encounter types on the same day, however, such as a physician appointment that leads to a hospitalization, would generally correspond to multiple encounters within the ENCOUNTER table. - Rollback or voided transactions and other adjustments should be processed before populating this table. - Although “Expired” is represented in both DISCHARGE_DISPOSITION and DISCHARGE_STATUS, this overlap represents the reality that both fields are captured in hospital data systems but with variation in how each field is populated. - Do not include scheduled encounters. - Partners should ensure that “administrative” encounters (e.g., e-mail, phone, documentation-only), are coded to the appropriate encounter type, which is typically “OA” for outpatient visits. |
| DIAGNOSIS | Diagnosis codes indicate the results of diagnostic processes and medical coding within healthcare delivery. Data in this table are expected to be from healthcare-mediated processes and reimbursement drivers. | - This table should capture all uniquely recorded diagnoses for all encounters, with the exception of problem list entries. If partners have access to multiple versions of each diagnosis within a given encounter (e.g., admitting, interim, final), the preference is to prioritize final or discharge diagnoses. A value should be specified in DX_SOURCE to indicate the classification of the diagnosis. - Diagnoses from problem lists will be captured in the CONDITION table. - If a patient has multiple diagnoses associated with one encounter, then there would be one record in this table for each diagnosis. - ENCOUNTERID should be populated for DIAGNOSIS and PROCEDURES. The definitions of the DIAGNOSIS and PROCEDURES tables are dependent upon a healthcare context; therefore, the encounter basis is necessary and the ENCOUNTERID, PROVIDERID, ENCOUNTER_TYPE, and ADMIT_DATE from the associated ENCOUNTER record should be included. While not desirable, a low percentage of orphan records is permissible to accommodate instances in which the associated ENCOUNTER details are missing from the source data. - Data in this table are expected to be from healthcare-mediated processes and reimbursement drivers, including technical/facility billing, professional billing and other data streams. **Do not omit** billing data unless it is unavailable from the source system or the partner is certain that the diagnoses loaded from the non-billing system (e.g., the EHR) represents completely the diagnosis data available from the billing system. Data from these different streams have different analytical utility so there is a benefit to including both if available. - Diagnoses are often only related to the **treatment** of the patient during the specific encounter. Chronic conditions that are not be pertinent to the treatment of a specific encounter, for example, would not be expected to be present. - If a local vocabulary is used, but *cannot* be mapped to a standard vocabulary such as ICD-9-CM, DX_TYPE should be populated as “Other” and the local value stored in DX. If the local value can be mapped to a standard vocabulary, follow the guidance around the population of Raw fields (General Guidance #1). - Partners should continue to populate ADMIT_DATE, even if they are populating DX_DATE. Analyses may leverage either date, or both. DX_DATE can be particularly useful for identifying diagnoses or conditions that might have developed over the course of a long inpatient encounter. |
| PROCEDURES | Procedure codes indicate the discreet medical interventions and diagnostic testing, such as surgical procedures and lab orders, delivered within a healthcare context. | - This table should capture all uniquely recorded procedures for all encounters, including office or evaluation and management visits, diagnostic testing, laboratory test orders, medication administrations, or other services rendered by a clinician. - If a patient has multiple procedures ordered during one encounter, then there would be one record in this table for each procedure. - ENCOUNTERID should be populated for DIAGNOSIS and PROCEDURES. The definitions of the DIAGNOSIS and PROCEDURES tables are dependent upon a healthcare context; therefore, the encounter basis is necessary and the ENCOUNTERID, PROVIDERID, ENCOUNTER_TYPE, and ADMIT_DATE from the associated ENCOUNTER record should be included. While not desirable, a low percentage of orphan records is permissible to accommodate instances in which the associated ENCOUNTER details are missing from the source data. - Data in this table are expected to be from healthcare-mediated processes and reimbursement drivers, including technical/facility billing, professional billing and other data streams. **Do not omit** billing data unless it is unavailable from the source system or the partner is certain that the procedures loaded from the non-billing system (e.g., the EHR) represents completely the procedure data available from the billing system - If a local vocabulary is used, but cannot be mapped to a standard vocabulary such as ICD-9-CM, PX_TYPE should be populated as “Other” and the local value stored in PX. If the local value can be mapped to a standard vocabulary, follow the guidance around the population of Raw fields (General Guidance #1). - Evidence of medications administered in outpatient settings should be present in the PROCEDURES table if that information is included with other billed/ordered PROCEDURES. - Evidence of inpatient administrations should be present in the PROCEDURES table if that information is included with other billed/ordered PROCEDURES. - **DO NOT** include records from medication administration sources (e.g., electronic medication administration records) in this table. - If possible to determine from the source data, only include procedures that have actually occurred. - **Inclusion of laboratory orders** – If possible, partners should include laboratory orders within the PROCEDURES table to support potential studies of appropriate laboratory monitoring. This includes those orders without a corresponding result in the LAB_RESULT_CM table. Do not include canceled orders. |
| VITAL | Vital signs (such as height, weight, and blood pressure) directly measure an individual’s current state of attributes. | - The deprecation of the VITAL table has been postponed. Partners should continue to populate VITAL with the relevant observations. Vital measures can also be stored in OBS_CLIN. - This table includes measurements recorded in both healthcare and non-healthcare settings. - The VITAL table contains one record per result/entry. Multiple measurements may exist in source data (for example, 3 blood pressure readings on the same day); in this case, each measurement would be a separate record. If multiple vitals are collected at the same time (e.g., height, weight and blood pressure recorded at the start of an encounter), it is permissible to store these values in a single record. This table should be populated with all available measures, with the possible exception(s) noted below. - If a partner has access to vital signs that are sourced from a device feed, they should make an assessment about data volume before including these measures, particularly if multiple readings per day are present for a large percentage of their population. Measures should not be averaged or aggregated.   - For healthcare device data sources: If multiple readings are available and the volume of data is judged by the data partner to be too burdensome for inclusion, using the set of values that were recorded directly in the medical record is preferred over any algorithmic selection process. - For personal device data sources: If multiple readings are available and the volume of data is judged by the data partner to be too high for inclusion, the project/study leadership should define a method for selecting individual measurements. ~~in the ETL ADD.~~ |
| DISPENSING | Prescriptions filled through a community, mail-order or hospital pharmacy. Outpatient dispensing may not be directly captured within healthcare systems. | - Each record represents an outpatient pharmacy dispensing. - This domain is commonly available in claims data, but may not be available in many EHR data sources. - Dispensing records are different from medication orders or prescribing records, data from medication administration activities, as well as the medication reconciliation of the active medication list. - Administered medications should NOT be stored in this table. They should be stored in the MED_ADMIN table. Evidence of medications administered in outpatient settings, such as infusions given in medical practices, or those administered in an inpatient setting may be present in the PROCEDURES table if that level of detail is available in the source procedure data. - Rollback transactions and other adjustments that are indicative of a dispensing being canceled or not picked up by the member should be processed (removed) before populating this table. This may be handled differently by Data Partners and may be affected by billing cycles. - In the uncommon situation where one NDC is dispensed more than once for a given patient on a given day, it is acceptable to combine the values from the multiple dispensings for days supply and number of units |
| LAB_RESULT_CM | This table is used to store quantitative and qualitative measurements from blood and other body specimens. | - Only records with actual lab results should be included in this table. If the result suggests that the test was run (e.g., result is "borderline" or "inconclusive") include it. But if the test is not resulted for any reason (specimen not sufficient, patient did not show), then do not include it. - If lab results are stored using local or custom codes, partners should ensure that the assigned LOINC code has been validated by a subject matter expert or similar process - If a LOINC code is available for a given result, the LAB_LOINC field should be populated. If a LOINC code is available for the *order*, that value can be used to populate the LAB_PX field. Note that one order can correspond to many different results. Each result should have its own record in the LAB_RESULT_CM table. If the same LOINC code is used to populate both the order and the result, partners should ensure that the LAB_LOINC field is populated. - **Inclusion of additional lab results -** Partners should include *all* available laboratory results within their LAB_RESULT_CM table. If the result has a *validated* LOINC code, the LAB_LOINC field should be populated. Otherwise, the LAB_LOINC field should be blank. The RAW_LAB_NAME field can be used to keep track of the various lab results until the appropriate LOINC code is assigned. Lab results beyond the 11 originally included in the PCORnet^®^ CDM are being requested in order to establish a denominator of potentially available lab results. Over time, the number of unmapped results is expected to decrease. Results for labs performed as a service for outside institutions do not need to be included. Results from external vendors (e.g., LabCorp, Quest) should be included when available. - **Clinical LOINC Concepts** – Only include Laboratory LOINC concepts in this table. Do not include clinical LOINC concepts (e.g., EKG results). These records may be stored in the OBS_CLIN table. - **Standing orders** - Partners should populate the date fields to the best of their ability. For results that are tied to standing laboratory orders, even if LAB_ORDER_DATE reflects the date of the original standing order, SPECIMEN_DATE and/or RESULT_DATE would be expected to correspond to the time when the sample was collected/resulted. Analyses will take both dates into consideration. - **Units of measure** – A given LOINC code may have many acceptable units of measure. If the RESULT_UNIT field is not populated, it may not be possible to use a result analytically. - **Verifying LOINC mappings** – At most health systems, laboratory results are typically not associated with a LOINC code at the time they are generated but are assigned a code after the fact. In order to verify that the LOINC code has been appropriately assigned, the ~~PCORnet~~ Coordinating Center will verify that the metadata associated with the result, such as SPECIMEN_SOURCE and RESULT_UNIT, are valid options. Partners should ensure that these fields are populated. **Do not derive these values based on metadata associated with the selected LOINC code.** - **Titers** – titer results that are returned as ratios (e.g., 1:4) should be stored in the RESULT_QUAL field even if these ratios are not explicitly enumerated in the QUAL valueset. Beginning in PCORnet^®^ CDMv6.1, data model conformance checks for RESULT_QUAL will include the enumerated list and text strings of [X]:[Y]. |
| CONDITION | A condition represents a patient’s diagnosed and self-reported health conditions and diseases. The patient’s medical history and current state may both be represented. | - This table includes both healthcare and non-healthcare settings. - Rollback or voided transactions and other adjustments should be processed (removed) before populating this table. - These records should NOT be duplicated in the DIAGNOSIS table. |
| PRO_CM | This table is used to store responses to patient-reported outcome measures (PROs), surveys, and questionnaires. This table can be used to store item-level responses as well as the overall score for each measure. | - The PRO_CM table can be used to store both individual item-level responses, as well as the overall score for the measure/instrument. In PCORnet^®^ CDM v7.0, the structure of the PRO_CM table was updated so that item responses and measure/instrument scores are each stored as separate records. If Network Partners find that a measure/instrument score does not have a corresponding identifier code in its respective terminology (e.g., PROMIS, LOINC), they should contact the Coordinating Center for assistance in choosing an appropriate code. - For the PRO_CM fields with variable field lengths, partners should choose an appropriate field length based on the characteristics of the data are loading into the table. As we use these tables analytically as part of PCORnet studies, we will determine whether it is more efficient to define specific field lengths. - If a patient completes a survey, but skips a question, create a record in the PRO_CM table as you would for other items in the survey (i.e., include the appropriate date/time fields and other relevant metadata). Then leave PRO_RESPONSE_TEXT and PRO_RESPONSE_NUM blank, as these fields are not required. Do not create empty records if the patient did not actually see the question. - The PRO_CM table can be used to store the results from questionnaires where the provider or caregiver are providing their interpretation or assessment of the patient’s status. Despite the name, it is not restricted solely to patient-reported outcomes. The table is designed to represent survey-type responses. General observations about patients, however, like pain scores recorded in an inpatient or surgical setting, should be stored in the OBS_CLIN table. See General Guidance #15 for additional details or contact the Coordinating Center with questions. - In general, patient-reported social determinants of health (e.g., food instability) would be expected to be found in this table. However, if partners who are participating in initiatives like the National COVID Cohort Collaborative have already loaded those records into OBS_GEN, it is acceptable for them to remain there. - For responses that can be represented as either a number or a text (e.g., a numeric value of 2 corresponds to “rarely” in a value set), the expectation is to store whatever is recorded in the source system. Both should be populated if present, but partners are not expected to derive the other if not. If values are represented based on a sequence number, store the actual value, not the sequence number. - The measure-related T-SCORE, STANDARD_ERROR and THETA fields have been deprecated as of PCORnet^®^ CDM v7.0. LOINC codes exist for these variables for some PRO instruments and can be used to store those values in this table. These variables are primarily found in the PROs that have been scored using a measure-specific Scoring Manual or the HealthMeasures Scoring Service, and are less likely to be present in the results of PROs captured in a health system setting, which may simply store the raw score for a measure. If assistance is needed on how to store these additional variables in the PRO_CM table, contact the Coordinating Center for assistance. |
| PRESCRIBING | Provider orders for medication dispensing and/or administration. These orders may take place in any setting, including the inpatient or outpatient basis. | - If a medication cannot be mapped to RxNorm, it should still be present and RAW_RX_MED_NAME should be populated. - This table can be used to store all medication orders, regardless of encounter type (e.g., inpatient, outpatient, ED) and can include orders for medications that are to be dispensed as well as for those that are to be administered. - If including orders derived through natural language processing (NLP), make sure that RX_SOURCE has been populated for all records. - See Reference Table 4 for the ordering strategy for RxNorm Term Types. - Do not populate PCORnet^®^ CDM fields with information derived from the RXCUI (e.g., RX_DOSE_ORDERED, RX_DOSE_FORM). Populate fields only if data are captured in the source system as a discrete value. - Populate records with the RXCUI as it existed at the time the order was entered, even if the RXCUI is no longer active. Do not attempt to update inactive RXCUIs with a more recent value. - Medications with approved formulations should have an RXCUI that can adequately represent all ingredients with a single code (e.g., SBD, SCD, MIN).  For medication mixtures that lack RXCUIs that can represent all of the component ingredients (e.g., IV mixtures prepared at an inpatient or compounding pharmacy), each individual medication from the order set should be included as a separate record with a unique PRESCRIBINGID. If partners wish to preserve the fact that the records belong to the same order, they do so by creating and populating a new *optional* ORDERID field. Medications with a 1:1 correspondence between the order and RXCUI could have the PRESCRIBINGID stored in the ORDERID. Orders with a 1:many RXCUI relationship would have different PRESCRIBINGIDs but the same ORDERID. Future versions of the PCORnet^®^ CDM may formalize this guidance. |
| PCORNET_TRIAL | Patients who are enrolled in PCORnet clinical trials and PCORnet studies. | - Partners may use the PCORNET_TRIAL table to maintain mappings between PCORnet^®^ CDM PATIDs and external trial or study IDs. - Partners wishing to use this table will need to register their TRIALID with the Coordinating Center. Please contact the Coordinating Center if you plan to utilize this table. - TRIALIDs that start with “PT_” and “PS_” are reserved for PCORnet Trials and PCORnet Studies. Partners should refrain from using TRIALIDs that start with these characters. - One patient participating in multiple trials or studies will have multiple records - Each PCORnet trial or study will define its parameters for study or trial participation, and will provide study-specific instructions on how to populate the fields in this table. - Patients who decline to participate in a trial or study or do not meet eligibility criteria should not be included in this table - Patients who enroll in a trial or study but later withdraw should be included in this table, with a date in the TRIAL_WITHDRAW_DATE field. - In most cases, trials will be expected to have a trial database that is separate from the PCORnet^®^ CDM - Randomization assignment is not included in this table due to the potential for unblinding. - PATID is not generally appropriate for use as a PARTICIPANTID because it is not disambiguated across networks. |
| DEATH | Reported mortality information for patients. | - One patient may potentially have multiple records in this table because their death may be reported by different sources. - Deaths represented in the ENCOUNTER.DISCHARGE_DISPOSITION and ENCOUNTER.DISCHARGE_STATUS would generally be expected to be present in this table (see field-level guidance for DEATH.DEATH_SOURCE). |
| DEATH_CAUSE | The individual causes associated with a reported death. | - When legacy data have conflicting reports, please make a local determination as to which to use. There is typically a 1-2 year lag in death registry data. |
| MED_ADMIN | Records of medications administered to patients by healthcare providers. These administrations may take place in any setting, including inpatient, outpatient or home health encounters. | - If a medication cannot be mapped to RxNorm or NDC, it should still be present and RAW_MEDADMIN_NAME should be populated. - Only include administrations that were actually delivered to the patient, if that level of specificity is available in the source system. - Patient-reported medication administrations are not within the scope of this table. - See Reference Table 4 for the ordering strategy for RxNorm Term Types. - Do not populate PCORnet^®^ CDM fields with information derived from the RXCUI (e.g., MEDADMIN_DOSE_ADMIN). Populate fields only if data are captured in the source system as a discrete value. - Populate records with the RXCUI as it existed at the time the order was entered, even if the RXCUI is no longer active. Do not attempt to update inactive RXCUIs with a more recent value. - If a medication mixture contains multiple RXCUIs (e.g., inpatient mixture), each individual medication from the order set should be included as an individual record with a unique MEDADMINID. Each individual medication is expected to have a unique dose. - ENCOUNTERID is expected to be present for records in the MED_ADMIN table. - For administrations where the amount ordered is listed as a rate (e.g., infusions), if the DOSE/DOSE_UNIT values are specified as a rate, and those fields are stored discretely in your EHR, populate the relevant PCORnet^®^ CDM fields. Assuming that START_DATE/START_TIME and STOP_DATE/STOP_TIME are also populated, it will be possible to compute the rate analytically. Otherwise, leave blank. |
| PROVIDER | Data about the providers who are involved in the care processes documented in the PCORnet^®^ CDM. | - Include one record per provider. - When populating provider specialty, if multiple values are available, use the specialty believed to be primary. |
| OBS_CLIN | Standardized qualitative and quantitative clinical observations about a patient, including vital signs. Some observations may also be represented in the VITAL table. | - The OBS_CLIN table is intended to store standardized clinical observations that have been recorded about a patient. - Examples of the types of observations that can be stored in this table include pulmonary function test results (e.g., FEV1, FVC, FEV1/FVC), echocardiogram results (e.g., left ventricle ejection fraction) and vital signs (e.g., temperature). - Vital signs that can be stored in the VITAL table may also be stored in this table (e.g., systolic blood pressure), though VITAL should continue to be populated. The Coordinating Center maintains a list of LOINC codes that can be used to represent these observations. - Decisions on what to include in this table and how to prioritize the population of those records are expected to be driven primarily by potential funding opportunities. - This table provides a generalized structure for storing observations and is not optimized for analytical efficiency. As elements from this table are used in studies and/or distributed queries, additional representations of those data elements (i.e., new table structures) may be required to better support those activities. - If partners are populating pain scores (not pain-related PRO surveys) captured in an inpatient or surgical setting, these values would be expected to be present in this table, not PRO_CM. - If an observation has a value set that includes an option of “not documented or assessed” (or similar), these values should be included in the PCORnet^®^ CDM if they are present in the source system. Do not derive if an observation is missing. - If a partner has access to vital signs that are sourced from a device feed, they should make an assessment about data volume before including these measures, particularly if multiple readings per day are present for a large percentage of their population. Measures should not be averaged or aggregated. - For healthcare device data sources: If multiple readings are available and the volume of data is judged by the data partner to be too burdensome for inclusion, using the set of values that were recorded directly in the medical record is preferred over any algorithmic selection process. - For personal device data sources: If multiple readings are available and the volume of data is judged by the data partner to be too high for inclusion, the project/study leadership should define a method for selecting individual measurements and this logic should be documented in the ETL ADD. - Observations recorded together (e.g., diastolic and systolic blood pressure) should have the same date(s) and time(s). |
| OBS_GEN | Table to store everything else. | - Partners may use this table to store network- or study-specific data elements. - Records in this table are not expected to be used in queries distributed by the Coordinating Center. - This table provides a generalized structure for storing observations and is not optimized for analytical efficiency. As elements from this table are used in studies and/or distributed queries, additional representations of those data elements (i.e., new table structures) may be required to better support those activities. |
| HASH_TOKEN | Encrypted hash tokens that are used to match patient records across DataMarts using privacy-preserving record linkage methods. | - Every patient in the DEMOGRAPHIC table is expected to have one record in the HASH_TOKEN table for each TOKEN_ENCRYPTION_KEY. - Tokens are generated from personally-identifiable information (PII). This information can be stored in each partner’s PRIVATE_DEMOGRAPHIC table and PRIVATE_ADDRESS_HISTORY table. The PII is used as input to the Datavant DeId/tokenization module. Tokens should not be placed into the PCORnet^®^ CDM until they have been transformed into Site-PCORnet transit tokens using the Datavant Link/transform-tokens module. - If PII fields are populated with dummy values by default (e.g., 999-99-9999 for phone number or SSN), these values should be removed before running the Datavant DeID/tokenization module. If PII is not available within the source system or if local restrictions prevent its use, leave the input field blank. - Tokens are generated based on data availability. If input data is not present for a given token strategy (e.g., combination of PII elements), no token will be generated and an error code will be produced instead. These token error codes should be loaded into the HASH_TOKEN table (i.e., there should not be any null values). Do not suppress the error codes in the output of the Datavant software. - Each successfully generated token has a fixed length of 44 characters. Do not enforce a 44-character constraint, however, to accommodate the error codes generated in the case of tokenization failure. - Tokens should be generated as part of every refresh. Partners can choose to generate tokens for all patients, or only for those patients who were added between refreshes or had updates to their PII. - Select tokens generated using the Datavant DeID/tokenization module are certified as de-identified data via the HIPAA Expert Determination method in accordance with the HIPAA Privacy Rule (45 CFR parts 160 and 164). All tokens in the HASH_TOKEN table satisfy this criteria and are controlled via the ~~Datavant DeID~~ PCORnet configuration settings within the Datavant software. - Additional token strategies are available and can be implemented as needed on a per-study basis based on the study-specific data dictionary. - See the Supplemental Guide on Privacy-Preserving Record Linkage for additional implementation details and guidance (separate document). - Partners should include the TOKEN_ENCRYPTION_KEY string that is output from the Datavant Link software. Please ensure that this table includes tokens encrypted using the PCORnet key (includes “pcornet” or similar in the output string). If partners are encrypting their tokens using multiple keys, it is acceptable to include those additional records in this table. |
| LDS_ADDRESS_HISTORY | Longitudinal record of a patient’s address that adheres to the requirements of a Limited Data Set. | - Expect multiple records per individual - This table is currently limited to addresses in the United States. - Partners can limit records in this table to validated addresses if known. - If partners have difficulty constructing a longitudinal address history for patients within their DataMart, they should prioritize populating the current address for each patient. |
| IMMUNIZATION | Records of immunizations that have been delivered within the health system as well as reports of those administered elsewhere. | - Do not include study vaccines. |
| HARVEST | Attributes associated with the specific PCORnet datamart implementation, including data refreshes. | - If partners need to impute date values, whether for a portion of the date (e.g., month) or the entire string, a value of “02” should be chosen for the relevant DATE_MGMT field(s). - If partners must impute the entire date for a field, this should only be done for those dates that are required. Optional fields should be left blank in these situations. - Partners should refrain from obfuscating dates within the PCORnet^®^ CDM, with the possible exception of BIRTH_DATE (see General Guidance #2). |
| LAB_HISTORY | Table for storing historical information about units of measure and reference ranges for laboratory test results. | - This table is intended to serve as a resource for partners as they develop their extract-transform-load (ETL) procedures to populate LAB_RESULT_CM. It is designed to store details related to units of measure and normal ranges for laboratory results that do not include such values at the record level. Partners can use this table as a reference during their ETL to look up and populate the relevant fields in LAB_RESULT_CM for those records. - It is expected that partners will be able to find information on units of measure and normal range via reference material maintained by their clinical labs (electronic documents or online catalogs). It is not necessary to derive this metadata from individual lab results. DO NOT DERIVE THESE DATA FROM LOINC RESOURCES. - While values for this table may need to be entered manually, a relatively small number of tests (~150) typically cover the vast majority of testing volume (>85%), which should minimize any data entry burden. - Partners may not need to populate this table, particularly if they are already able to meet the network lab data quality metrics. - Partners do not need to create records in this table for results that come from external reference labs (e.g., Quest, LabCorp). The Coordinating Center can provide this information on request. - The Coordinating Center may reference this table if labs are needed as part of a particular study or analysis and the units and/or reference range is missing from the result(s). Partners may be asked to populate records in this table for those corresponding tests. - Every record in this table should be unique. |
| PAT_RELATIONSHIP | This table is used to store information about the relationships between patients within a given PCORnet^®^ Common Data Model. | - For the RELATIONSHIP_TYPE field, the ordering of the terms indicates the directionality of the relationship. - The RELATIONSHIP_START and RELATIONSHIP_END fields are intended for use with non-biological relationship types where a start and end date may be present (e.g., spouse, legal guardian-child). These fields are not required and health systems may not have the information necessary to populate them, but remain available for use. |
| EXTERNAL_MEDS | Medications external to the originating health system. Examples include medications as reported by the patient and those obtained from external health systems. | - This table is intended to store medication orders / prescriptions from external health systems as well as patient-reported medications. Dispensing information from external prescriptions should be stored in the DISPENSING table. - The format of the EXTERNAL_MEDS table mimics that of PRESCRIBING, though some of the metadata fields (e.g., FREQUENCY) that are expected to be poorly populated have been removed. - The EXTERNAL_MEDS table uses RxNORM CUIs to represent records. Certain dietary supplements that are not FDA-approved may not have an associated CUI. The RXCUI field for these records should be left blank. |
